# Glycosylation Weakens Skp1 Homodimerization in Toxoplasma gondii by Interrupting a Fuzzy Interaction

**DOI:** 10.1021/acs.biochem.4c00859

**Published:** 2025-04-29

**Authors:** Donovan A. Cantrell, Ramona J. Bieber Urbauer, Hyun W. Kim, Robert J. Woods, Jeffrey L. Urbauer, Zachary A. Wood, Christopher M. West

**Affiliations:** †Department of Biochemistry and Molecular Biology, ‡Department of Chemistry, §Complex Carbohydrate Research Center, ∥Center for Tropical and Emerging Diseases, ⊥Center for Molecular Medicine, 1355 University of Georgia, Athens, Georgia 30602, United States

## Abstract

Skp1/Cullin-1/F-Box protein (SCF) complexes represent a major class of E3 ubiquitin ligases responsible for proteomic control throughout eukaryotes. Target specificity is mediated by a large set of F-box proteins (FBPs) whose F-box domains interact with Skp1 in a conserved, well-organized fashion. In the social amoeba *Dictyostelium*, Skp1 is regulated by oxygen-dependent glycosylation which alters Skp1’s FBP interactome and inhibits homodimerization that is mediated in part by an ordered interface which overlaps with that of FBPs. Based on sedimentation velocity experiments, Skp1 from the intracellular pathogen Toxoplasma gondii exhibits a homodimerization *K*_d_ comparable to that of a previously measured FBP/Skp1 interaction. Glycosylation of Skp1’s disordered C-terminal region (CTR) distal to the ordered homodimer interface significantly weakens Skp1 homodimerization, an effect reproduced by CTR deletion. Replacement with a randomized CTR sequence retains high affinity excluding an extension of the ordered dimer interface. Substitution by poly serine weakens the homodimer to a degree equal to its deletion, indicating a composition dependent effect. The contribution of the CTR to Skp1 homodimerization is canceled by high salt consistent with an electrostatic mechanism. All-atom molecular dynamics simulations suggest that the CTR promotes homodimerization via charge cluster interactions. Taken together, the data indicate that glycosylation weakens homodimerization by disrupting a C-terminal fuzzy interaction that functions in tandem with the ordered dimer interface, thereby freeing Skp1 for FBP binding. Thus, the CTR contributes to Skp1/Skp1 and Skp1/FBP interactions via independent mechanisms that are each influenced by O_2_, indicating multiple constraints on the evolution of its sequence.

## Graphical abstract

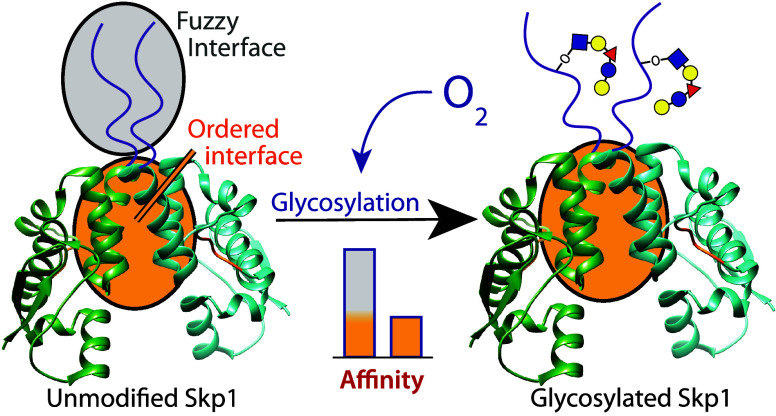

## Introduction

The local concentrations of O_2_ that surround cells provide important information regarding metabolic potential, oxidative risks, and positional information.^1^ In particular, the sensing of O_2_ gradients can assist free-living protists to seek optimal niches, pathogenic protists to home to favorable locations, and individual cells of multicellular organisms to form patterns.^2^ As a specific example, the apicomplexan pathogen Toxoplasma gondii, upon oral ingestion, navigates a complex trajectory from its entry via the alimentary canal to achieve chronic and latent infections in distant target tissues, and gradients of O_2_ concentrations are likely to provide guidance. T. gondii is an obligate intracellular parasite and the causative agent for Toxoplasmosis.^3^ Infection poses a major threat to the fetus of previously unexposed pregnant women, and for immune compromised individuals poses a serious risk of debilitating injury of the brain and eyes. Following progression from an initial acute infection, healthy individuals progress to a chronic phase owing to immune control. An estimated 11% of the United States population over the age of 6 is thought to be latently infected with T. gondii, and the frequency approaches 80% in some Western countries. Persistent latent infections mean that once individuals become infected, they are at risk of significant life-threatening disease if they become immune compromised later in life. This threat is exacerbated by limited treatment options for clearing persistent *Toxoplasma* infections forcing immune compromised individuals to undergo treatment for life.^3^

A conserved prolyl hydroxylase, from which human PHD2 likely evolved,^4^ has been implicated in O_2_-sensing in the social soil amoeba *Dictyostelium discoideum*, an unrelated protist that is more closely related to metazoans. By using O_2_ as a cosubstrate, PhyA regulates the O_2_ checkpoint for a process called culmination, when multicellular *Dictyostelium* slugs commit to converting into sessile fruiting bodies after migration to the O_2_-rich surface of the soil.^2,5,6^ The *Toxoplasma* ortholog regulates fitness in cell culture^7^ and dissemination from the gut in a mouse infection model.^8^ In both organisms, PhyA specifically modifies a conserved Pro residue of Skp1, a subunit of the SCF (Skp1/cullin-1/F-box protein/Rbx1) class of E3 ubiquitin ligases that generate poly ubiquitin tags that target proteins for proteasomal degradation. Target protein specificity is mediated by dozens (or more) of F-box proteins (FBPs) serving as substrate receptors and, in some cases, the generation of degron-inducing posttranslational modifications of the protein target.^9^

The mechanism of PhyA’s action is unclear, but several clues have emerged. In both organisms, the enzyme modulates the FBP interactome of Skp1 ^10–12^ and is associated with differential abundance of select FBPs by a mechanism that appears to involve their degradation.^12^ Another clue is that hydroxylation inhibits the homodimerization of *Dictyostelium* Skp1,^13^ which is competitive with FBP-Skp1 interactions.^14^ Other evidence for the significance of dimerization comes from recent studies in nematode worms, where Skp1 paralogs are specialized for an essential non-SCF role in meiotic synaptonemic complexes that require their homodimerization.^15,16^ The action of PhyA enables the posttranslational assembly on the resulting hydroxyproline (Hyp) of a defined linear pentasaccharide of almost identical sequence on Skp1 from both organisms as a result in part of convergent evolution.^2^ Genetic evidence indicates that glycosylation mediates the action of PhyA,^10,11^ leading us to further investigate the mechanism by which the oligosaccharide influences the structural and biochemical properties of Skp1.

Skp1 is highly conserved throughout eukaryotic phylogeny, but its posttranslational hydroxylation and glycosylation appears to be confined to the protist domain of life.^17^ Crystallographic evidence from complexes with FBPs from animals, yeast and plants reveals four C-terminal α-helices that comprise subsite-1 and subsite-2 of the interface with F-box domains,^18^ and an upstream N-terminal region that docks to the cullin-1 scaffold (Figure 1A). Cul1 in turn links to the Ub-charged E2 via the RING-protein Rbx1 ^19^ to complete the horseshoe-shaped SCF Ub-ligase with the target docked at its open end. The Hyp-linked glycan, which is located at the start of the final C-terminal helix-8 that comprises subsite-2, is expected to reside on the backside of the F-box interface. In its free form, NMR and AUC studies show that both mammalian and *Dictyostelium* Skp1 exist as a homodimer with a *K*_d_ in the 1–2.5 μM range.^14,20,21^ Detailed studies of DdSkp1 reveal that its individual monomers fold in a similar manner to that seen in complexes with FBPs up through α-helix-6. However, the downstream region that can fold as α-helices-7 and −8 with FBPs is partially disordered, and the organization of this C-terminal region (CTR) is influenced by O_2_-dependent glycosylation.^13,14,21^ A truncated version lacking the CTR retains the ability to homodimerize, with an interface that involves α-helices-5 and −6 and therefore competes with F-box binding. The semiparallel orientation of the dimer mates would orient the missing C-termini in juxtaposition with one another. Because of its intrinsic disorder in the homodimer, we hypothesized that the CTR contributes to homodimerization by a mechanism different from that used to bind FBPs and distinctly modulated by glycosylation.

**Figure 1:**
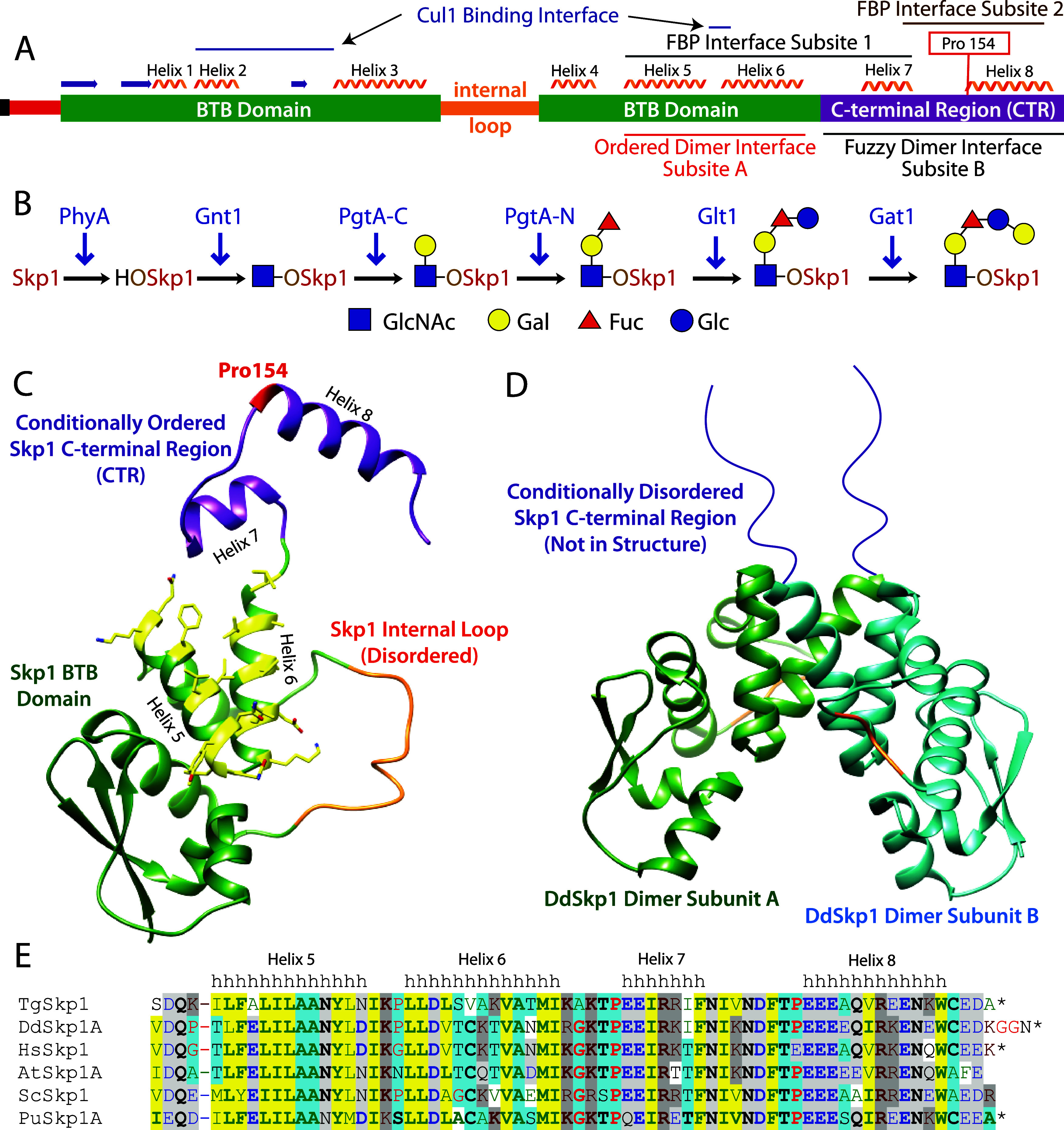
Structure models of *Toxoplasma* Skp1. (A) Domain diagram for TgSkp1 (UniProt A0A0F7UNE6). Secondary structures, intermolecular contact sites, and the glycosylation site at Pro154 are indicated above. Skp1 consists of an N-terminal SerMet (black) extension remaining from removal of a His_6_-tag, a disordered segment of N-terminal amino acids (red), a globular BTB domain (green), an intervening disordered internal loop (orange), and a C-terminal region (CTR) which is disordered when not bound to an FBP (purple). The homodimer subsites are indicated below. (B) Glycosylation pathway responsible for the canonical, linear glycosylation of TgSkp1 initiated by the O_2_-dependent prolyl-4 hydroxylase PhyA. (C) Homology model for TgSkp1 bound to an FBP. Color coding is as in panel A. α-helixes-5, −6, and −7, which contribute to subsite-1, and α-helix-8 which contributes to subsite-2, are depicted. Amino acids at the homodimer interface are shown in yellow as sticks. (D) Previously determined structure of the truncated DdSkp1 homodimer (PDB ID: 6V88), with the location of the missing CTR arbitrarily represented in extended form. (E) Alignment of the C-terminal TgSkp1 sequence associated with FBP and self-binding (i.e., contributing to helixes 5–8 in complexes with FBPs) with corresponding regions from Skp1s across phylogeny. Amino acids are colored according to their chemical class, in bold if extensively conserved, and highlighted if the chemical class is highly conserved across phylogeny. Key to species: Hs, Homo sapiens; At, Arabidopsis thaliana; Sc, Saccharomyces cerevisiae; Pu, Pythium ultimum.

To confirm Skp1’s homodimerization and the influence of its glycan, we turned our attention from *Dictyostelium* to *Toxoplasma* Skp1 and at the same time quantified the role of the CTR and its glycosylation. Like DdSkp1, truncated TgSkp1 lacking the CTR homodimerizes with a μM *K*_d_. Significantly, the presence of the CTR contributes over an estimated order of magnitude decrease in the *K*_d_ rendering it potentially competitive with FBP binding.^22^ Our further biochemical studies characterized a contribution of the CTR to homodimerization based on criteria predicted for a charge-based fuzzy interaction,^23–25^ which was supported by all-atom molecular dynamics (MD) simulations, and we quantified the inhibitory role of the glycan. We propose that O_2_-dependent glycosylation of Skp1 interferes with the charge-based fuzzy interaction thereby providing a facile mechanism to increase the availability of monomeric Skp1 for its physiological role in SCF complex formation.

## Materials and Methods

### Generation of Skp1 Variants

Full-length His_6_-TgSkp1 (UniProt A0A0F7UNE6) was expressed from pNIC28-HisTEVTgSkp1 as previously described.^26^ Staggered primers were designed to delete the CTR or replace the internal loop (Figure S19) using PCR reactions with GXL-Prime STAR polymerase (TaKaRa) or SuperFi Platinum polymerase (Thermo-Fisher). Clones were screened by colony PCR utilizing primers specific for the pNIC28 plasmid (Figure S19). Positive clones were sequenced utilizing full plasmid nanopore sequencing or Sanger Sequencing initiating from both directions.

The sequence for TgSkp1’s C-terminal region (CTR) was randomized 6 times using the Hanging Hyena word scrambler.^27^ Scrambled sequences were appended to the TgSkp1-ΔCTR sequence and analyzed at the NetSurfP-2.0 server to evaluate propensity to form ordered secondary structure.^28^ Two sequences least likely to form ordered secondary structure were codon optimized and synthesized by GenScript in the pNIC28 plasmid (Figure S20).^29^ Similarly, the poly-Ser TgSkp1 variant was created by replacing the CTR coding region with Ser codons.

### Purification of Skp1 Constructs

The Skp1 expression constructs were transformed into ER2566 strain Escherichia coli for protein production. Clones were grown in Terrific Broth at 37 °C in the presence of 50 μg/mL kanamycin.^29^ After reaching an OD_600_ of 1.0, cultures were shifted to 18 °C for 30 min and then induced by addition of IPTG to 500 μM for 16–18 h. Cells were recovered by centrifugation and frozen at −80 °C.

Pellets were resuspended in Lysis Buffer (50 mM Na/K phosphate (pH 7.4), 300 mM NaCl, 5 mM benzamidine, 10 μg/mL leupeptin, 10 μg/mL aprotinin, and 0.5 μg/mL pepstatin A) or Super Lysis Buffer (100 mM Na/K phosphate (pH 7.4), 150 mM NaCl, 0.5 M D-sorbitol, 1 M glycine betaine, 0.7 M trehalose, 5 mM benzamidine, 10 μg/mL leupeptin, 10 μg/mL aprotinin, and 0.5 μg/mL pepstatin A). Suspended pellets were lysed using 3 passes through a French Pressure cell before centrifugation at 27,000 × *g* for 40 min. Supernatants were further clarified by spinning at 100,000*g* for 70 min before loading onto a 5 mL Talon column (TaKaRa). The column was washed with 50 mL each of Wash Buffer (50 mM Na/K phosphate (pH 7.4), 300 mM NaCl), High Salt Buffer (50 mM Na/K phosphate (pH 7.4), 1 M NaCl), Glycerol Buffer (50 mM Na/K phosphate (pH 7.4), 300 mM NaCl, 10% (v/v) glycerol), and Wash Buffer. Bound protein was then eluted with Elution Buffer (50 mM Na/K phosphate (pH 7.4), 300 mM NaCl, 300 mM imidazole (pH 7.4)). Eluted proteins were immediately dialyzed into 50 mM Tris-HCl (pH 7.4), 150 mM NaCl, 0.1 mM EDTA (pH 8.0) before being dialyzed into 50 mM Tris-HCl (pH 7.4), 150 mM NaCl, 1 mM DTT. TEV protease cleavage was performed as previously described,^26^ and after 2 consecutive dialysis steps against Wash Buffer, samples were passed over a second 5 mL Talon Column, concentrated to 50 mg/mL by centrifugal ultrafiltration, divided into 0.5 mL aliquots, and frozen in liquid nitrogen for later use. Protein properties including extinction coefficients used in quantifying the constructs were calculated using the ProtParam ExPASy server.^30^ Skp1 constructs prepared in this way yielded greater than 20 mg/L.

### Purification of Skp1 Glycosyltransferases

TgGlt1 and DdPgtA were purified as previously described.^31,32^ TgGat1 was expressed with an autoinduction method utilizing lactose allowing for slow expression.^33,34^ E. coli Gold cells containing pET15-His-TEV-GT8a­(Gat1)^33,34^ were grown to confluence in 200 mL of Luria Broth starter culture supplemented with 100 μg/mL ampicillin for ∼16 h. 50 mL of the starter culture was used to inoculate 1 l of Autoinduction medium (3 g/L KH_2_PO_4_, 6 g/L Na_2_HPO_4_ (pH 7.2), 20 g/L Tryptone, 5 g/L Bacto yeast extract, 5 g/L NaCl, 100 μg/mL ampicillin, 0.52 g/L d-glucose, 2.1 g/L lactose, 0.6% (v/v) glycerol). Cultures were grown at 37 °C for 2 h before shifting to 18 °C for 46 additional h. Cells were pelleted by centrifugation at 5000*g* for 10 min and frozen at −80 °C until purification as described above for Skp1. TgGat1 was typically recovered at >20 mg/L of culture.

### Modification of TgSkp1

The pNIC28-HisTEVTgSkp1 plasmid for expressing His-tagged/TEV cleavable TgSkp1 was transformed into E. coli strain ER2566 together with pETDuet-DdPhyA-Gnt1Chim^35^ for expression of untagged DdPhyA and chimeric Gnt1 for the hydroxylation and GlcNAcylation of TgSkp1 (Figure 2D). Gn-TgSkp1 was purified as above, and its modification confirmed using MALDI-TOF MS. For analysis, samples were desalted, spotted on a plate with sinapinic acid using the sandwich method, air-dried, and analyzed in an ABI MALDI-TOF MS operated in linear positive ion mode. Gn-TgSkp1 (0.12 mg/mL) was incubated for 16 h at room temperature with 5 μg/mL DdPgtA in 24 μM UDP-Gal (4-fold excess over TgSkp1), 42 μM GDP-Fuc (7-fold excess over TgSkp1), 10 μg/mL leupeptin, 10 μg/mL aprotinin, 10 mM MgCl_2_, 2 mM MnCl_2_, 0.1% (v/v) Tween-20, 50 mM Tris-HCl (pH 7.4), 5 mM DTT, 120 mM NaCl, and 0.12 U/mL Shrimp alkaline phosphatase, with modification monitored using MALDI-TOF-MS. The reaction mixture was loaded onto a 1-ml Ni-Sepharose column (GE Healthcare), which was then washed with 50 mM Na/K phosphate (pH 7.5), 300 mM NaCl, 20 mM imidazole, and eluted with 50 mM Na/K phosphate (pH 7.5), 300 mM NaCl, 500 mM imidazole. Skp1 was concentrated to 3 mg/mL and buffer exchanged into 50 mM HEPES-NaOH (pH 8.0) using a 10k-Da cutoff centrifugal ultrafiltration device. FGGn-TgSkp1 (0.77 mg/mL) was incubated for 16 h at 37 °C with 0.05 mg/mL TgGlt1, 280 μM UDP-Glc (8-fold excess over Skp1), 10 μg/mL leupeptin, 10 μg/mL aprotinin, 2 mM MgCl_2_, 2 mM MnCl_2_, 50 mM HEPES-NaOH (pH 8.0), 5 mM DTT, and 2.4 U/mL Shrimp Alkaline Phosphatase. After confirmation of conversion to GlFGGn-TgSkp1 by MALDI-TOF-MS, the sample was buffer exchanged using a 10-kDa cutoff centrifugal ultrafiltration device to 50 mM HEPES-NaOH (pH 7.0), concentrated to 2 mg/mL, incubated at 35 μM (0.7 mg/mL) with 1.75 μM (0.07 mg/mL) TgGat1, 140 μM UDP-Gal (4-fold excess over Skp1), 10 μg/mL aprotinin, 10 μg/mL leupeptin, 2 mM MnCl_2_, 2 mM MgCl_2_, 0.2% Tween-20, 50 mM HEPES-NaOH (pH 7.0), 5 mM DTT, and 0.1 U/mL Shrimp Alkaline Phosphatase for 16 h at 37 °C, and checked using MALDI-TOF-MS to confirm complete conversion to GGlFGGn-TgSkp1. TEV cleavage occurred concurrently with the TgGlt1 and TgGat1 reactions on account of inadvertent contamination from TEV-treatment of Glt1. The sample was then purified over a 5 ml HiTrap Q HP ion exchange column (Cytiva) followed by a Superdex 75 (16/600) preparative gel filtration column (Cytiva) into 50 mM K phosphate (pH 7.4), 25 mM KCl, and checked for purity using MALDI-TOF-MS and SDS-PAGE (Figure 2B,C). SDS-PAGE was performed using InVitrogen NuPage preformed 4–12% SDS-PAGE gels in MES buffer, and gels were stained in Coomassie Brilliant Blue.

**Figure 2:**
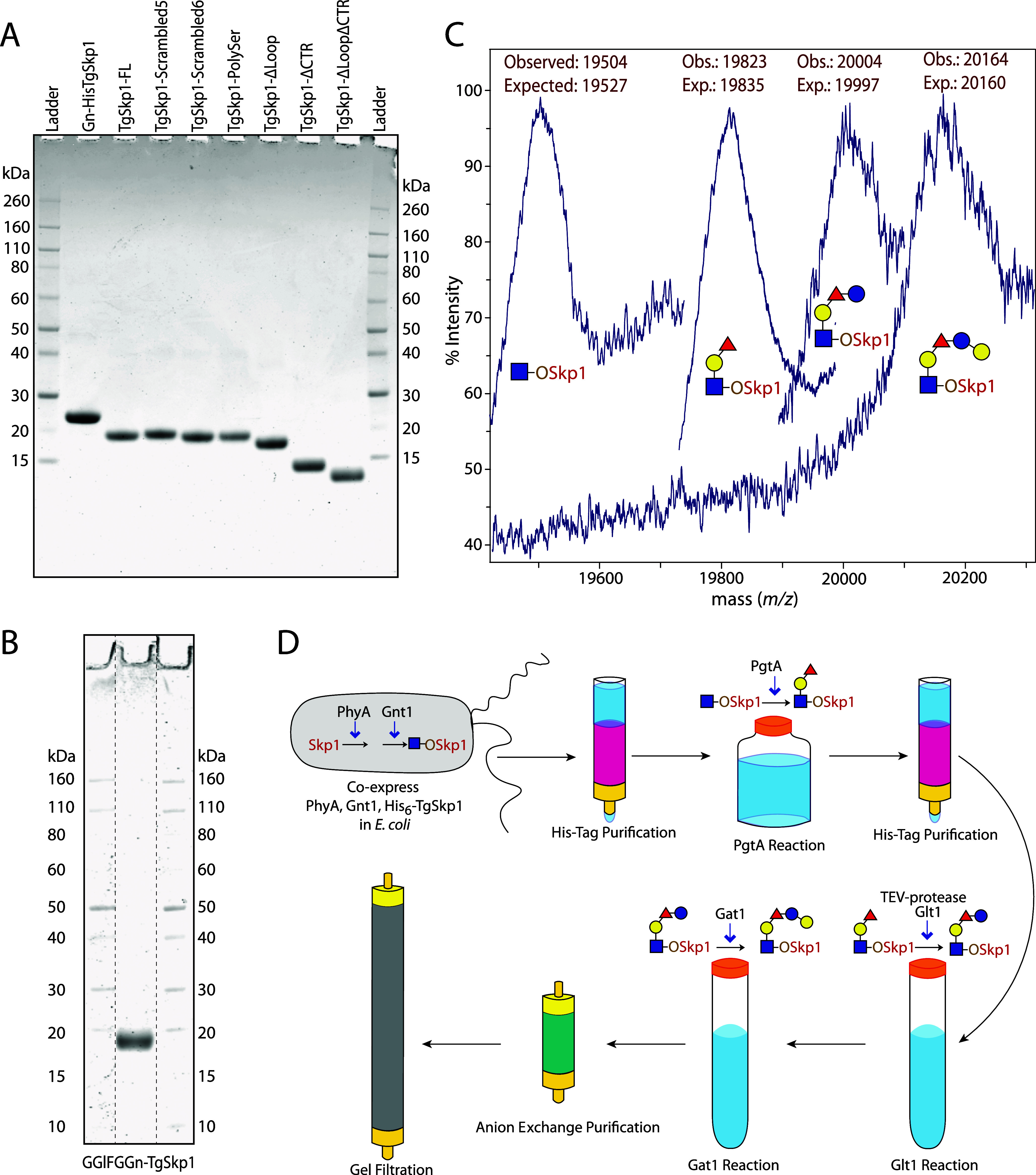
TgSkp1 isoforms and preparation of glycosylated TgSkp1. (A) Coomassie Brilliant Blue gel stained SDS-PAGE gels loaded with 3 μg protein each. (B) Same for fully glycosylated TgSkp1. Lanes excerpted from the same gel are denoted by dashed lines. (C) Alignment of MALDI-TOF mass spectra demonstrating sequential conversion between each glycoform in untagged TgSkp1. (D) Schematic for generating glycosylated TgSkp1.

### Sedimentation Velocity Experiments

Skp1 aliquots were thawed at room temperature, supplemented with 1 mM Na EDTA (pH 8.0), and 450 μL was rerun on a Superdex 75 (16/600) column in freshly degassed 50 mM K phosphate (pH 7.4), 25 mM KCl. The peak fraction was serially diluted in the same buffer, and aliquots were loaded into 12 mm double-sector Epon centerpieces with quartz windows before equilibration for 2 h at 20 °C. Samples were centrifuged on a Beckman Optima XLA Analytical Ultracentrifuge at 50,000 rpm at 20 °C, and absorbance measurements were collected at 215, 230, 280, or 295 nm (depending on the concentration) with a 0.003 cm step size. Buffer and protein parameters were calculated using SenNTerp and c(s) distributions were modeled using SedFit.^36,37^ Data were modeled as a continuous c(s) distribution and fitted using baseline, meniscus, frictional coefficient, and noise parameters. Distributions were overlaid in Gussi and the region corresponding to the monomer and dimer species were integrated to determine the average *S*_w_ position of each distribution.^38^ These values were plotted in SedPhat where a Monomer–Dimer self-association model was utilized to derive the affinity of each Skp1 construct.^39^ Predicted *S*_w_ values for the monomer and dimer species were obtained using HYDROPRO^40^ using models generated using Modeler,^41^ SwissModel,^42^ or MD simulations utilizing DdSkp1ΔloopΔCTR (PDB: 6V88) as the template.

### Circular Dichroism and Thermal Melts

Skp1 was dialyzed overnight against degassed 20 mM K phosphate (pH 7.5), 10 mM KCl, 1 mM DTT, and diluted to 5–13 μM based on *A*_280_ in a UV–vis spectrophotometer. Thermal stabilities were assessed by measuring temperature induced unfolding. Unfolding was followed using circular dichroism (CD) by monitoring the ellipticity at 222 nm at 1 °C intervals as the proteins were heated from 20 to 90 °C at a rate of 1 °C per min. Refolding (90 to 20 °C) was followed in the same manner. Far UV CD spectra were acquired at 20 °C before unfolding, at 90 °C, and again at 20 °C upon refolding. Data were collected from 260 to 190 nm in 1 nm increments with a scan rate of 100 nm/min, with four spectra averaged to give a final spectrum. CD measurements were performed using a Jasco J-715 spectropolarimeter with a Peltier temperature controller and a cuvette with a 1 mm path length. Thermal unfolding experiments were performed 2–3 times for each of the five protein constructs. As described previously,^43^ the fractions of folded and unfolded states were determined at each temperature and the Gibbs–Helmholtz equation was used to describe the thermodynamics as a function of temperature. For all proteins the heat capacity was assumed to be equal to zero. Data fitting employed the Levenberg–Marquardt algorithm as implemented in the Kaleidagraph^44^ general curve fit utility.

### Analysis of the TgSkp1 Dimer using All-Atom MD

Starting molecular coordinates for the TgSkp1 dimers were generated using the Modeler homology modeling software.^41^ The lowest energy conformer of the solution NMR structure of DdSkp1AΔloopΔCTR (PDB: 6V88) was used as a template. Due to this construct lacking either the internal loop or CTR, these segments were modeled in the extended conformation. Six homology models were generated with their disordered segments in an extended conformation, and three were chosen based on their stability during preliminary MD simulations for further analysis. MD simulations were performed with the pmemd.cuda version of AMBER22.3.^45^ Amino acid residues were parametrized with the ff14SB force field.^46^ The system was built in a truncated octahedral box with a 15 Å distance from the solute to the end of the unit cell, neutralized with Na^+^, and solvated using TIP3P water.^47,48^ Electrostatic interactions were simulated using the particle mesh-Ewald algorithm with a nonbonded cutoff of 8 Å.^49^ SHAKE was used to constrain hydrogen-containing bonds allowing for a step size of 2 fs. Solvent minimization was conducted over 100,000 cycles with steepest descent being used for 500 cycles with conjugate gradient being used for the remaining cycles with solute being restrained with 100 kcal/mol Å^2^. Total system minimization was conducted over 100,000 cycles with steepest descent being used for 500 cycles with conjugate gradient being used for the remaining cycles. The system was heated to 300 K under NVT (constant particle number, volume, and temperature) conditions over 60 ns before being held steady at 300 K for an additional 10 ns. Conditions were then changed to NPT (constant particle number, pressure, and temperature) and the system was equilibrated for 140 ns before a 1 μs production phase.

Every 0.5 ns frame of the production phase was utilized for MMGBSA calculations with the mmpbsa.py script.^50^ Chain A was treated as the receptor and Chain B as the ligand. An igb of 2 was utilized while an idecomp of 3 was used to generate pairwise interaction energies and an idecomp of 1 was used to generate per residue total interaction energies. Total per residue interaction energies were averaged across all three simulations and between dimer mates for a total of six molecules. Pairwise interaction energies were organized into matrixes and the portion of the matrix corresponding to the interaction of Chain A with Chain B was extracted. Interaction energies associated with each interaction (BTB/BTB; BTB/C-term; C-term/C-term) were added using the matrix and the total value was divided by two to normalize for each dimermate. The interactions between oppositely charged CTR residues showing interaction energies greater than −1 kCal/mol in magnitude were further analyzed to observe association and dissociation of charged groups. Atoms used to represent these groups were CZ for Arg, NZ for Lys, CD for Glu, and CG for Asp.

Cluster analysis was carried out using the MD Ensemble Cluster analysis tool in UCSF Chimera^51^ which groups frames in the production run into groups of similar structures or ensemble clusters and selects a representative structure as previously described.^52^ Three 1 μs simulations were compiled and one frame every 10 ns was loaded for analysis. This resulted in 300 total frames being analyzed with the identification of 25 total clusters. The representative structure/frame from each cluster was then analyzed using HYDROPRO^40^ and the s-value was obtained by a weighted average based on the number of frames in each cluster.

## Results

### TgSkp1 is a Sub-Micromolar Affinity Homodimer

Throughout eukaryotic phylogeny, Skp1 consists of a globular BTB/POZ (BTB for BR-C, ttk and bab or POZ for Pox virus and Zinc finger) domain with an internal loop, and a C-terminal region (CTR) where, in many Protists, Pro hydroxylation and glycosylation occurs (Figure 1A).^14,17^ In 20 structures of Skp1 with FBPs from humans, plants, and yeast,^53–55^ Skp1 adopts the fold represented in Figure 1C, in which the CTR typically organizes as a pair of α-helices, helix-7 and helix-8 separated by a loop. In only one known FBP-Skp1 complex (Fbs1) does the prospective α-helix-8 appear to fail to fold in this way.^56^ α-helices-5–7 form a bundle that represents the core interface with the F-box, referred to as subsite-1, and α-helix-8 contributes the so-called variable interface^18^ referred to here as subsite-2 (Figure 1A,C). In contrast, free mammalian and *Dictyostelium* Skp1s form soluble homodimers.^14,20^ The NMR structure of a truncated version of DdSkp1 lacking the CTR and having its internal loop replaced with GGSG also formed a stable homodimer.^14^ The BTB/POZ domain was organized essentially identically to its fold in complexes with FBPs (Figure 1D). The interface involved α-helices-5 and −6, which also contribute to the subsite-1 contact surface with FBPs. This predominantly hydrophobic interface is referred to as the ordered interface or subsite-A with respect to self-dimerization. The CTRs were found to be disordered,^21^ and Figure 1D illustrates the juxtaposition of the CTRs, which were absent from the construct, as imposed by the ordered homodimer interface. The high level of sequence conservation of TgSkp1 (Figure 1E) indicates that it will adopt similar conformations as for these examples. Under normoxic conditions, PhyA hydroxylates Skp1 on a conserved proline (Pro154 in TgSkp1), which anchors the canonical O-linked glycan (Figure 1B).^57^ The contribution of the CTR to homodimerization and the influence of glycosylation are the subject of this report.

TgSkp1 was recombinantly expressed in E. coli and purified essentially to homogeneity based on SDS-PAGE (Figure 2A). The recombinant protein lacked detectable posttranslational modifications based on intact protein mass spectrometry but was extended by a SerMet-stub preceding the normally processed Ser N-terminus after proteolytic removal of the His_6_-tag. Using sedimentation velocity analysis in an analytical ultracentrifuge to assess self-interaction (Figure S1), 50 μM full-length TgSkp1 (TgSkp1-FL) exhibited a sedimentation coefficient (S) of 2.7, in good agreement with the predicted value using cluster analysis of MD simulations (Figure 3A, Table 1). The conclusion that this peak represents a dimer species is reinforced by a matching dimer position with the similarly sized and structured DdSkp1 and TgSkp1 constructs where a monomer–dimer equilibrium was observable (Table 1).^14^ At the lowest concentration that could be accurately monitored by absorbance at 215 nm, 300 nM TgSkp1-FL remained largely dimeric (Figure 3A). Peak broadening and a slightly reduced s-value (to 2.5) indicated partial but apparent rapidly equilibrating dissociation. Modeling the sedimentation of the monomer subunit using Hydropro yields a sedimentation value of 1.4–1.5, though comparison with other full-length variants indicates a sedimentation coefficient of 1.6. By estimating the degree of dissociation at 300 nM to be ∼ 18% based on these values and assuming noncooperative binding, a *K*_d_ of approximately 50 nM is inferred.

**Figure 3:**
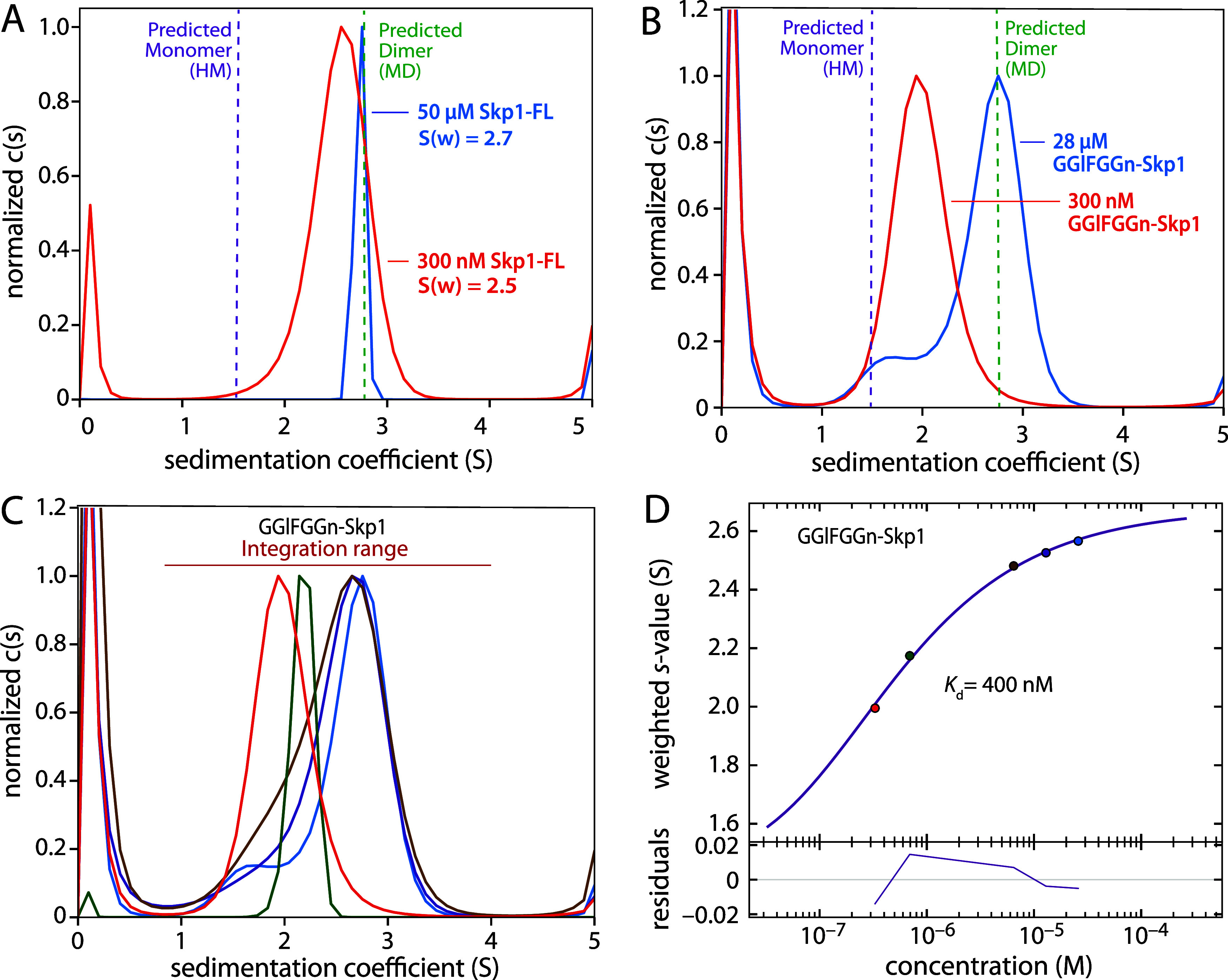
Dimerization of native and glycosylated Skp1. (A) c(s) distributions from sedimentation velocity AUC experiments with TgSkp1-FL at 50 μM (blue) and 300 nM (orange). The dashed lines represent the predicted s-values for monomeric TgSkp1-FL as predicted by modeler (HM, purple) and the dimer as predicted by MD cluster analysis (green). (B) c(s) distributions of GGlFGGn-TgSkp1. (C) c(s) distributions across a concentration range (color coordinated with panel D values) of GGlFGGn-TgSkp1. (D) A *S*_w_ isotherm derived from integration of the color coordinated data in panel C was used to derive the homodimerization *K*_d_ value for GGlFGGn-TgSkp1. Deviations of measured values from the fitted curve are plotted in the bottom panel as residuals. Supporting c(s) distributions are in Figures S1, S2.

**Table 1:** Predicted and Observed Sedimentation Values for the TgSkp1 Monomer and Dimer Species^a^

**expected from hydropro**	**S, monomer**	**S, dimer**
TgSkp1-FL (MD)	NA	2.7
TgSkp1-FL (HM)	1.4–1.5	2.1–2.3
TgSkp1ΔCTR (HM)	1.5–1.6	2.3–2.5
**experimental/observed**		
TgSkp1-FL	NA	2.7
TgSkp1ΔCTR	1.4	2.6
TgSkp1-poly-Ser	1.6	2.8
TgSkp1-Scrambled5	1.6	2.8
TgSkp1-Scrambled6	NA	2.7
TgSkp1Δloop	2	2.6
TgSkp1Δloop, high salt	2.3	2.9
TgSkp1ΔloopΔCTR	1.5	2.4
TgSkp1ΔloopΔCTR, high salt	1.8	2.4
GGlFGGn-TgSkp1	1.5	2.8

a Expected sedimentation values were predicted using Hydropro and either homology models (HM) for TgSkp1ΔCTR, TgSkp1-FL, or representative MD structures of TgSkp1-FL. Observed sedimentation values were taken from the maximum and minimum positions of each c(s) isotherm or from the dimer positions of the highest concentration (TgSkp1-FL, TgSkp1-Scrambled6).

### Glycosylation Inhibits Self-Association

To characterize the effect of the glycan, TgSkp1-FL was coexpressed in E. coli with TgPhyA and DdGnt1 which resulted in a near homogeneous preparation of Gn-TgSkp1-FL (Figure 2A,C). The glycan was completed *ex vivo* with recombinant DdPgtA, TgGlt1, and TgGat1, resulting in highly purified GGlFGGn-TgSkp1 modified with the full-length linear pentasaccharide as assessed by MALDI-TOF mass spectrometry (Figure 2A–D). 300 nM GGlFGGn-TgSkp1 sedimented with an s-value of 1.9 (Figure 3B), midway between the predicted s-values of the monomer and dimer states, suggesting that at this concentration GGlFGGn-TgSkp1 exists in a rapid equilibrium on the time scale of the experiment. The s-value gradually increased at higher concentrations reaching 2.7 at 28 μM indicative of the dimer species, with only a minor peak observed at an s-value of 1.7, slightly higher than expected for the monomer state (Figure 3B, Table 1). A concentration series of c(s) distributions (Figure S2) were deconvolved into monomer and dimer contributions and plotted as an isotherm to determine the *K*_d_ (Figure 3C). The calculated *K*_d_ of 400 nM (Figure 3D) indicates a substantial weakening of homodimerization due to glycosylation, as summarized in Table 2. Given the location of the glycan near the middle of the CTR, we next turned to examining its role in dimerization.

**Table 2:** Calculated Affinities of TgSkp1 Isoforms^a^

**construct**	***K***_**d**_
TgSkp1-FL	∼50 nM*
TgSkp1ΔCTR	900 nM
TgSkp1-poly-Ser	900 nM
TgSkp1-Scrambled5	200 nM*
TgSkp1-Scrambled6	∼50 nM*
TgSkp1Δloop	200 nM*
TgSkp1Δloop, high salt	8500 nM
TgSkp1ΔloopΔCTR	2400 nM
TgSkp1ΔloopΔCTR, high salt	1900 nM
GGlFGGn-TgSkp1	400 nM

a *indicates calculation by extrapolation.

### TgSkp1’s C-Terminal Region (CTR) Contributes to Dimerization

NMR studies previously indicated that the CTR of free dimeric DdSkp1 exists in a partially disordered state,^21^ which was consistent with ITC studies suggesting that complexing of human Skp1 with an FBP caused a relatively large disorder to order transition.^22^ The role of the CTR was first assessed by its truncation at 3 residues past the C-terminus of α-helix-6 (Figure 4A), by analogy to that of DdSkp1ΔCTR. The similar circular dichroism spectra recorded for the FL and ΔCTR variants (Figure 4B) are consistent with a lack of stable secondary structure in Skp1’s CTR, and the decreased % unordered content (Figure S4K) supports this interpretation. In addition, thermal denaturation experiments indicated a single transition in which the Skp1 constructs had minimal differences in *T*_m_, 61.3 ± 0.4 °C for TgSkp1-FL and 60.2 ± 0.2 °C for TgSkp1ΔCTR (Figure S4A–D), consistent with TgSkp1ΔCTR being properly folded. 300 nM TgSkp1ΔCTR sedimented with a predominant species at S = 1.5, close to the monomer position predicted by Hydropro (Figure 4C, Table 1). At the highest concentration tested (220 μM), TgSkp1ΔCTR sedimented at 2.4, closely matching the *S* = 2.5 value predicted for the TgSkp1ΔCTR dimer (Figure 4C,E). An isotherm generated using the weighted s-values for the monomer and dimer positions yielded a *K*_d_ of 900 nM for TgSkp1ΔCTR (Figures 4D,E, S3). This represents an 18-fold decrease in affinity relative to the estimated *K*_d_ for TgSkp1-FL, or an expected energetic cost of ∼ 1.7 kcal/mol. This is unlikely to be due to an entropic effect of the loss of the 34 amino acids *per se*, because replacement with an uncharged poly-Ser chain failed to strengthen the homodimer (Figures 5E, S8, Table 1). The simplest interpretation for the inhibitory effect of glycosylation is that it disrupts the contribution of the CTR to homodimerization.

**Figure 4:**
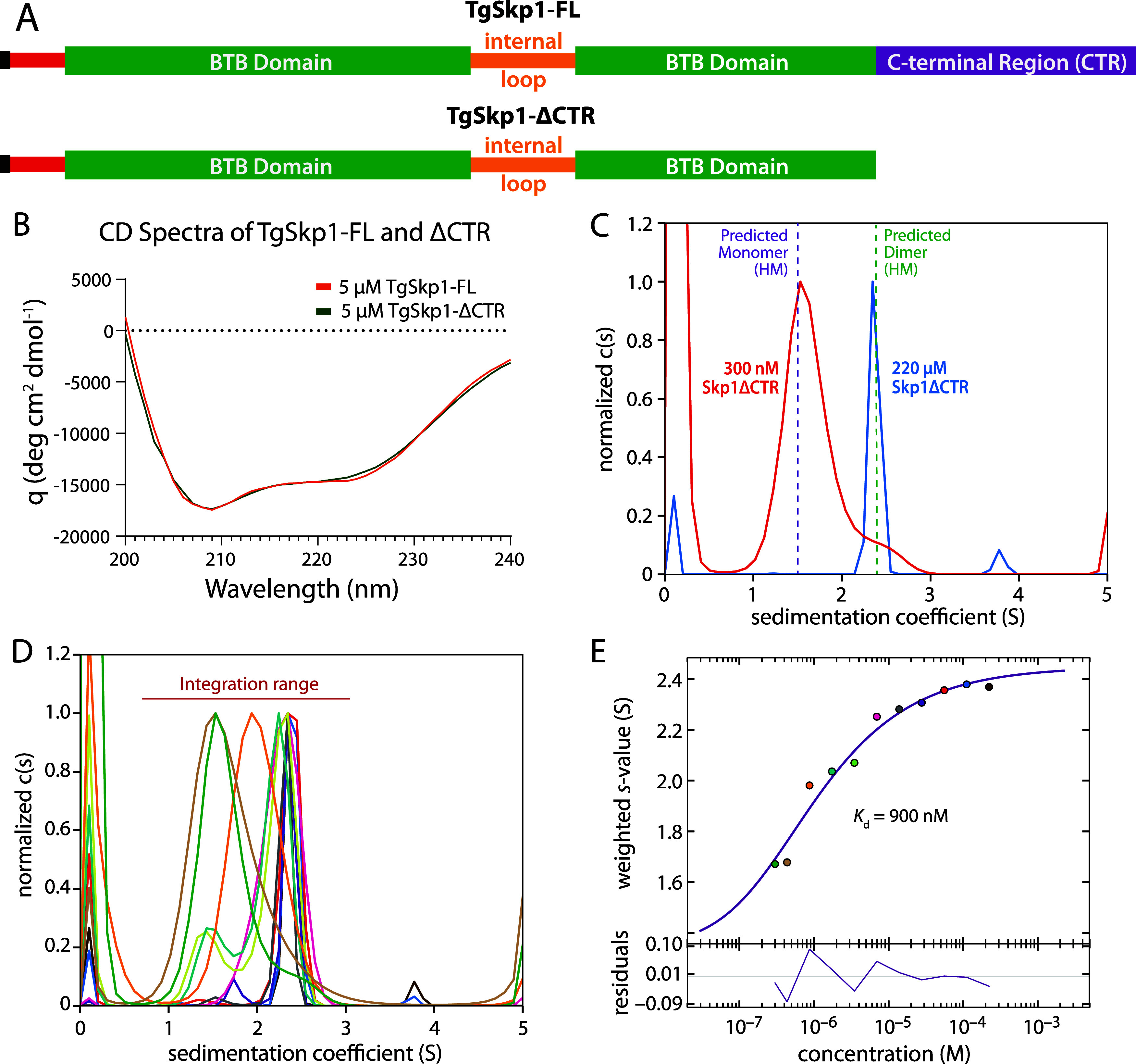
Contribution of the C-terminal region (CTR) to Skp1 homodimerization. (A) Domain diagrams of TgSkp1-FL and the TgSkp1-ΔCTR variants. (B) Circular dichroism spectra of the TgSkp1-FL and TgSkp1ΔCTR constructs. (C) c(s) distributions from sedimentation velocity experiments of TgSkp1-ΔCTR at two concentrations, as in Figure 3A,B. (D) c(s) distributions over a range of concentrations to generate the *S*_w_ isotherm in panel E. (E) *S*_w_ isotherm with residuals, as in Figure 3D. Supporting c(s) distributions are in Figure S3.

**Figure 5:**
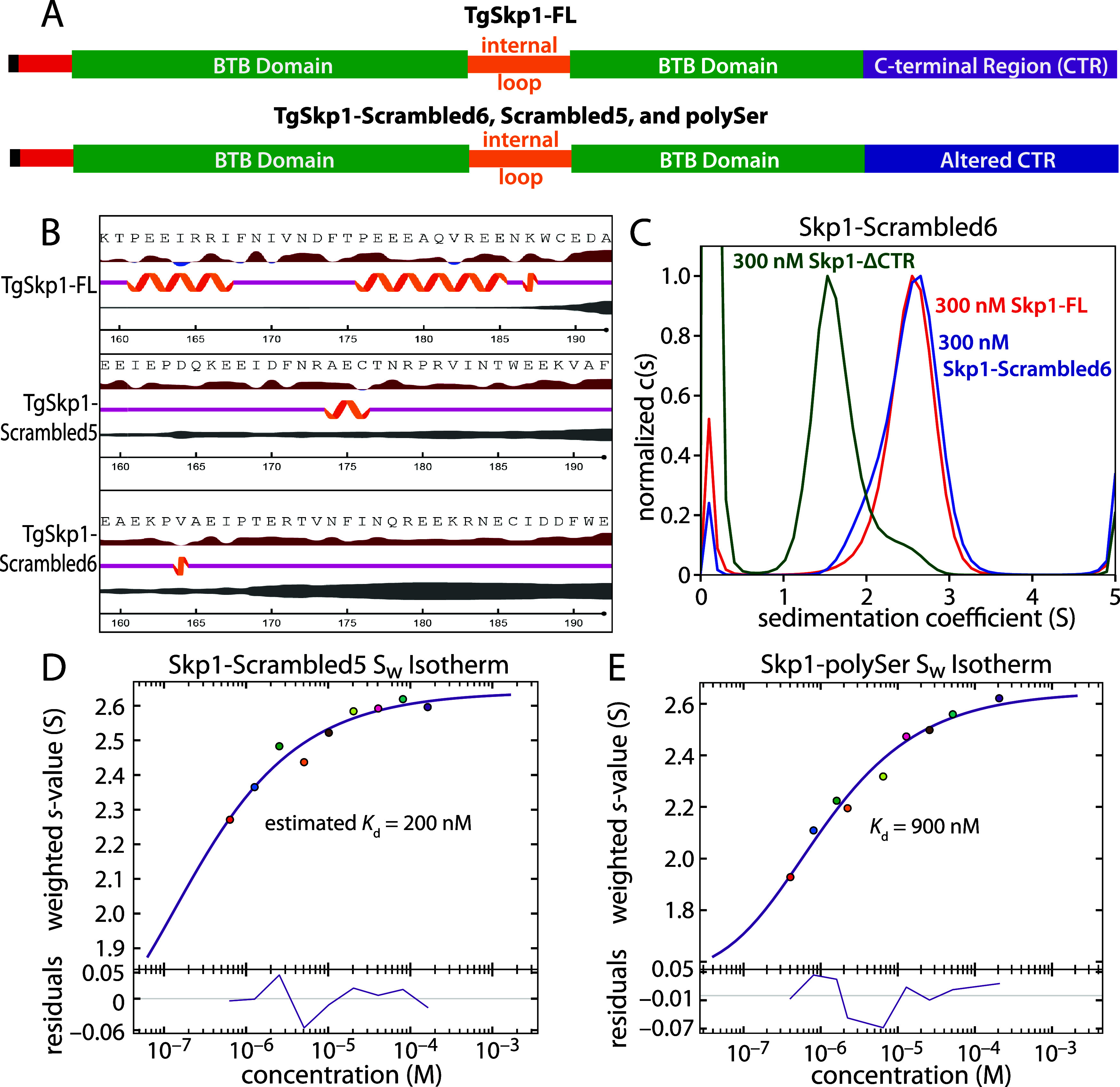
Sequence and composition dependence of the CTR contribution to dimerization. (A) Domain diagrams for TgSkp1-FL and CTR sequence variants. (B) Native and scrambled CTR sequences are shown with NetSurfP-2.0 predictions for solvent exposure (red = likely, blue = unlikely), secondary structure (orange), and disorder (gray bar thickness). (C) c(s) distributions for TgSkp1-FL (orange), TgSkp1-ΔCTR (green), and TgSkp1-Scrambled6 (blue). (D) *S*_w_ isotherm for TgSkp1-Scrambled5, with residuals at the bottom. (E) *S*_w_ isotherm for TgSkp1-poly-Ser, as in panel D. See Figures S5–S8 for supporting c(s) distributions.

### Amino Acid Composition of the Skp1 C-Terminal Region is More Important than its Sequence

To probe the mechanism by which TgSkp1’s CTR could drive dimerization, its sequence was first scrambled (Figure 5A). A series of scrambled sequences appended to the C-terminus of Skp1’s BTB domain were submitted to NetSurfP-2.0 to predict their propensity to form disordered coils or secondary structure (Figure 5B). Two top sequences were chosen based on minimal predicted secondary structure and high likelihood for disorder. As for the TgSkp1-FL and ΔCTR constructs, thermal denaturation experiments with these scrambled variants as well as the poly-Ser construct showed a single transition with minimal differences in thermal stability (Figure S4). Sedimentation velocity experiments on The TgSkp1-Scrambled6 variant had % unordered content comparable to TgSkp1-FL based on CD (Figure S4K), and formed a high affinity homodimer with an apparent affinity similar to the native sequence (Figures 5C, S6). The lack of sequence specificity suggested that the CTR drives dimerization while in a disordered state. This was reinforced by nearly identical sedimentation velocities of the native and Scrambled6 proteins consistent with similar overall structures (Table 1). A degree of sequence dependence was however observed for the TgSkp1-Scrambled5 variant. A *S*_w_ isotherm modeled a *K*_d_ of 200 nM (Figures 5D, S5A, S7), intermediate between values for TgSkp1-FL and TgSkp1ΔCTR. A mild to moderate sequence dependence has been observed elsewhere for the electrostatic association of proteins,^58–62^ indicating that charge distribution can play a major role in association and, as discussed below, the Scrambled5 sequence lacks directly paired positively charged residues found in the FL and Scrambled6 sequences. The nearly identical sedimentation velocities of the native, the two scrambled, and the poly-Ser dimer constructs (Table 1) largely exclude a difference in 3-dimensional structure, and their near identical *T*_m_ values indicate minimal disruption of structure or stability (Figure S4). However, CD studies noted increased % unordered in the Scrambled5 and poly-Ser constructs at the expense of α-helix content which, interpreted against all the other data, might reflect challenges faced in modeling CD data of proteins with unique structural properties.^63^ These findings, paired with the negligible contribution of a poly-Ser replacement (Figure 5E, Table 1), are inconsistent with a length dependent mechanism, which excludes an entropic tuning model^64^ for how Skp1’s CTR drives dimerization. Taken together, these data indicate that the CTR drives dimerization through an amino acid composition dependent mechanism which, considering the high proportion of charged (44%) and polar (18%) amino acids and low proportion of hydrophobic amino acids (26%), might involve an electrostatically driven interaction.

### Deleting TgSkp1’s Internal Disordered Loop Weakens Dimerization

DdSkp1 contains an internal intrinsically disordered loop based on sequence prediction and NMR analysis.^21^ Twelve residues of this 16-amino acid loop have been often replaced by a short linker peptide (GGSG) to facilitate crystallization of complexes with FBPs,^14,18,55,65^ and was similarly replaced in the DdSkp1 homodimer NMR structure study to reduce size. However, preliminary studies suggested that restoration of this internal loop promoted dimerization (data not shown) of the F97E mutant of DdSkp1.^14^ Thus the significance of the internal loop for TgSkp1 was tested by replacement with GGSG (Figure 6A). Although TgSkp1-Δloop still formed a relatively tight homodimer, its *K*_d_ increased to 200 nM, near the lower limit of detection with our system (Figures 6B, S5C, S9).

**Figure 6:**
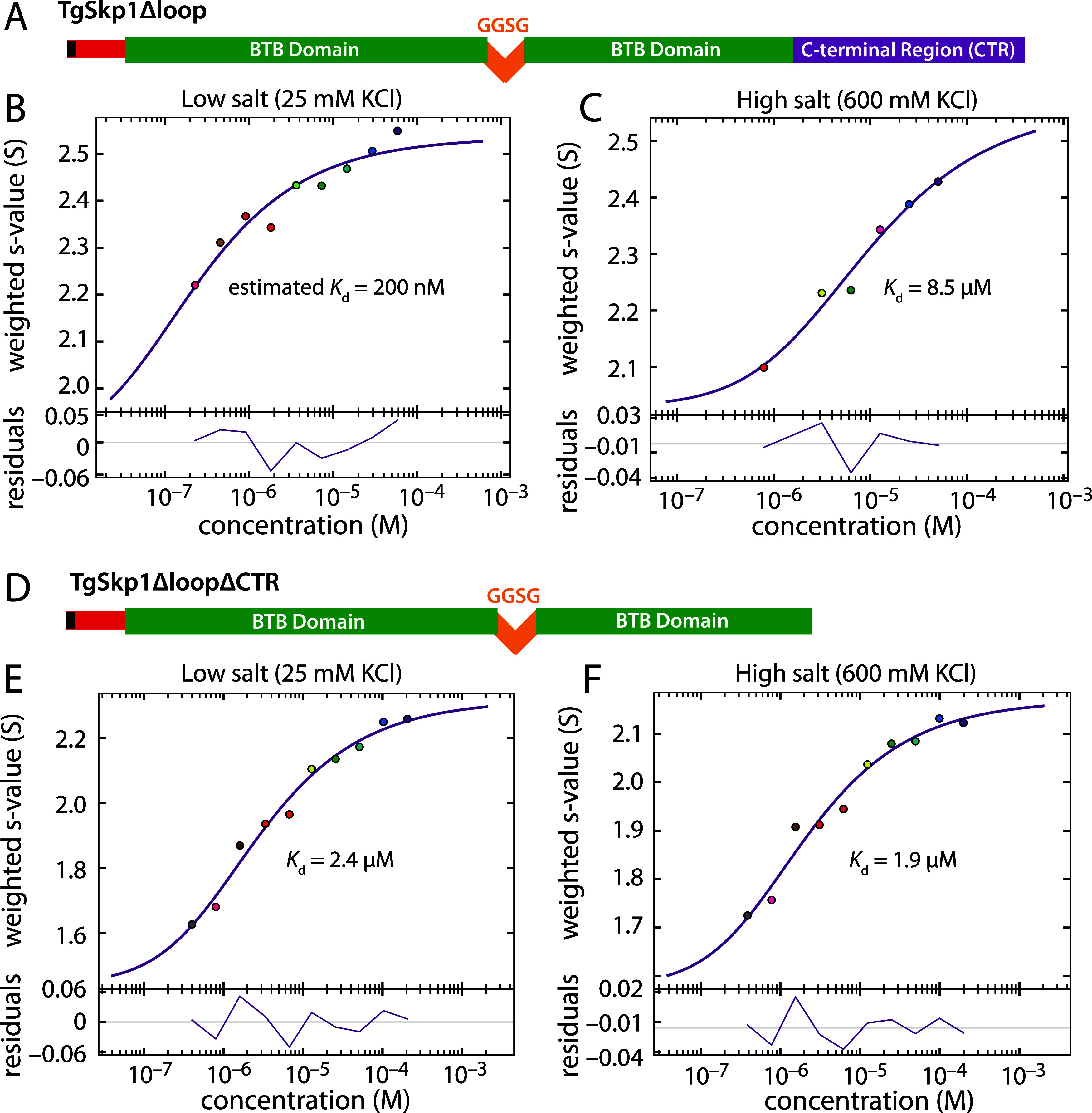
Salt sensitivity of the CTR contribution. (A) Domain diagram for TgSkp1Δloop. (B) *S*_w_ isotherm was used to determine the homodimerization affinity using the standard buffer (25 mM KCl, 50 mM K/H PO_4_ (pH 7.4)). The *K*_d_ was estimated using an isotherm modeled by integrating the monomer and dimer positions of Skp1 based on data shown in Figure S5. (C) Same as panel B except for the presence of 600 mM KCl (high salt). (D) Domain diagram for TgSkp1ΔloopΔCTR. (E, F) *S*_w_ isotherms used to determine the homodimerization affinity in standard buffer (E) or high salt buffer (F) for TgSkp1ΔLoopΔCTR. Deviations of measured values from the fitted curves are plotted in the bottom panels as residuals. See Figure S5, 9–12 for supporting c(s) distributions.

Deletion of Skp1’s CTR against the Δloop background revealed, as expected, a weakened affinity compared to TgSkp1Δloop (Figure 6D,E). Integration of the c(s) distributions yielded a *K*_d_ of 2.4 μM (Figures 6E, S5E, S10 **)**, or about a 10-fold loss of affinity relative to TgSkp1ΔloopΔCTR, indicating an energetic contribution from the CTR of about 1.36 kCal/mol that was comparable to the difference between the TgSkp1-FL and TgSkp1ΔCTR homodimers. Shortening the loop enabled comparison of affinities without the extrapolations required for the higher affinity interactions.

### Skp1’s C-terminal Region Drives Dimerization via a Salt-Sensitive Mechanism

We then examined a potential role for electrostatics in the positive contribution of Skp1’s CTR to dimerization by raising the ionic strength of the solution to a value shown to interfere with electrostatic interactions in other examples.^58,59,66,67^ AUC experiments up to this point had utilized a buffer containing 50 mM K/Phosphate and 25 mM KCl at pH 7.4. This yields an ionic strength of about 140 mM within the physiological range expected for mammalian cells.^68^ At 600 mM KCl (high salt; ionic strength of 710 mM), a sedimentation velocity isotherm for TgSkp1Δloop showed a substantial increase in *K*_d_ from ∼200 nM to 8.5 μM (Figures 6B,C, S5D, S11). In contrast, the affinity of TgSkp1ΔloopΔCTR dimerization was hardly affected (Figures 6E,F, S12, S5F), changing from a *K*_d_ of 2.4 μM at normal ionic strength to 1.9 μM at high salt, indicating a minimal electrostatic contribution from the remaining subsite-A. This was not unexpected as TgSkp1’s ordered dimer interface, whose sequence is similar to that of DdSkp1 (Figure 1),^14^ has a primarily hydrophobic character. An effort to test the effect of high salt on TgSkp1-FL was confounded by higher order aggregation at an s-value of 3.8–3.9 that was only observed at ≥100 μM for TgSkp1-FL, preventing assessment of CTR-Loop interactions to homodimerization. Inhibition of TgSkp1Δloop homodimerization by high salt can be attributed to an effect on the CTR and applies another constraint on the nature of the interaction: that it is dominated by polar and electrostatic forces in addition to being dynamic.

### Skp1’s C-Terminal Region Participates in a Fuzzy Self-Association

To investigate how high salt inhibits dimerization, we utilized all-atom MD simulations with explicit water. The Modeler homology modeling software was used to generate full-length models of the TgSkp1 dimer using the DdSkp1AΔloopΔCTR homodimer as the template. Unlike previous studies where the starting conformations were borrowed from the FBP bound state,^34,69^ separate extended conformations for the disordered protein segments based on more recent data^21^ were modeled to reduce bias. Over the course of the first 140 ns, the CTRs were found to entangle into varied and dynamic conformations that are referred to as collapsed. Following this transition, a 1-μs production run was used for energetic analysis through MMGBSA. Figure 7 summarizes results from the first of 3 simulations. Assessment of total per residue energetic contributions highlighted both the ordered dimer interface and a second electrostatically driven interface, subsites-A and -B respectively (Figures 7A, S13, S14). The strong ordered dimer interface matches closely with that previously characterized for DdSkp1A by NMR, with major contributions from F108 (F97 in DdSkp1), I134 (I123), and L112 (L101).

**Figure 7:**
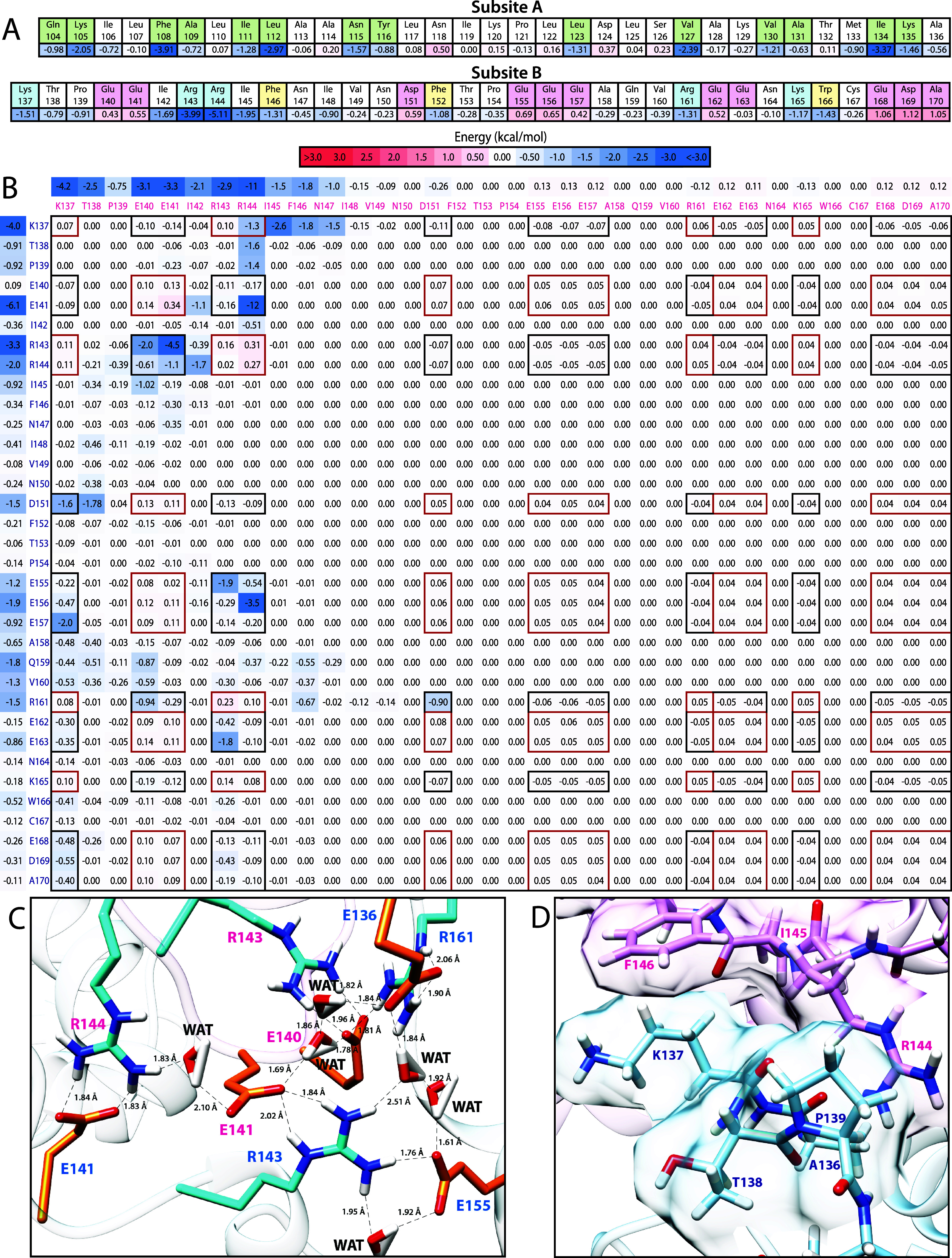
Skp1 CTRs self-associate through an electrostatically driven fuzzy interaction. (A) In tabular form, energetic contributions for residues corresponding to subsites A and B are shown. Residues previously shown as part of the dimer interface are highlighted in light green. In the CTR (subsite-B) positively charged residues are highlighted in light blue, negatively charged residues in light pink, and aromatic residues in yellow. (B) An interaction energy matrix or heat map for the pairwise interaction energies of each CTR residue from Simulation 1. Residue labels corresponding to Chain A are colored blue while residue labels corresponding to Chain B are colored magenta. Pairwise energies representing the interaction of oppositely charged residues are boxed in black while energies representing the interaction of identically charged residues are boxed in red. Individual residue contributions were calculated by adding all CTR pairwise interactions and dividing by two. (C) An example of direct and indirect interactions of oppositely charged residues, annotated with atom distances. Positive residues are colored in cyan while negative residues are colored in orange. Residue labels corresponding to Chain A are colored blue while residue labels corresponding to Chain B are colored magenta. Chain A is light blue while Chain B is plum. (D) Directly interacting residues are shown as sticks with their van der Waals surfaces, with chains colored as in panel C. See Figures S15, S16 for Simulations 2 and 3.

Each subunit’s CTR largely contributed to dimerization via direct interactions with the CTR of the opposite dimer subunit, however some contribution of CTR-BTB interactions were also observed (Table 3). Productive CTR-CTR contacts were observable across the length of all three simulations (Figure S17). The bound states for CTR interactions were conformationally diverse with residence times primarily in the sub-5 ns range (Figures 7B, S15A, S16A, S17). Qualitative analysis indicated contributions from both proximal and distal CTR-CTR conformations. Favorable interactions were largely centered around consecutive positively or negatively charged residues, or charge clusters. Interaction of opposite charges was observed through both direct residue–residue contacts and interaction of each residue’s hydration shell (Figure 7C, S15B, S16B). For comparison, these direct interactions and kinetics closely resemble the association of residues observed in all-atom MD simulations of ProTα and H1 phase separation events.^70^ Although all positively charged residues participated in favorable electrostatic interactions, the bulk of favorable interaction energy contributed by positively charged residues came from the paired R143-R144 charge cluster (Figures 7A, S14). This is in line with data from the scrambled variants as the weaker TgSkp1-Scrambled5 dimer lacks directly paired positively charged residues like the Scrambled6 and wild-type examples (Figures 5B, 1E). The majority of negatively charged residues rendered a net weakening effect on dimerization (Figures 7A, S14). This is likely due to the repulsive effects of the net negative charge of TgSkp1 and its CTR, where acidic outnumber basic residues by a 10:5 ratio resulting in fewer favorable binding states. The ability to form favorable binding states despite the identical charge of each subunit likely lies in the solvent’s electrostatic screening of distant charges (>8 Å) modeled here using the particle mesh-Ewald algorithm.^49^

**Table 3:** Energetic Components for the Interaction of Each TgSkp1 Protein Region^a^

	**electrostatic**	**polar solvation**	non-polar solvation	**van der Waals**	**total**	**average deviation**
**(CTR) (BTB)**	160	–160	–6.3	–8.4	–23	±4.9
**(CTR) (CTR)**	39	–51	–7.9	–10	–30	±8.0
**(BTB) (BTB)**	130	–140	–14	–21	–42	±2.5
				**total energy**	–96	±12

a Pairwise interaction energy components between TgSkp1 regions are derived from the MD simulations and given in kcal/mol. The association between each subunit’s BTB region is primarily contributed by Skp1’s ordered dimer interface (subsite-A). The association of each subunit’s CTR with both the BTB and CTR region of the opposite subunit indicates Skp1’s fuzzy interface. The CTR-CTR and BTB-BTB rows represent each respective single interaction. The CTR-BTB row represents the sum of intermolecular interactions between the CTRs and BTB domains of each subunit.

In addition to electrostatic effects, a positive neighborhood effect was observed for residues near interacting charge patches (Figures 7B, S15A, S16A). The neighborhood effect is largely attributed to nonpolar contacts occurring when charge patches bring residues in proximity to each other and limit the mobility of adjacent peptide chains (Figures 7D, S15C, S16C). This neighborhood effect is particularly pronounced in nonpolar residues near the R143-R144 charge patch and aromatic residues within the CTR (Figures 7A, S14). This effect may contribute to a relatively stable bound conformation as observed for the R144:E141 interaction of Simulation 1 compared to states limited to charge–charge interactions (Figure S17). This is in line with other studies that found residues nearby primary intrinsically disordered protein interactions can contribute to auxiliary interactions.^71^

## Discussion

Here we propose a role for a dynamic, charge based, fuzzy interaction between identical sequences, that acts in concert with a second ordered site to define the full homodimer interface of Skp1. The contribution of the disordered CTR to homodimer affinity was substantial in terms of affinity measured using AUC. A decrease in *K*_d_ from 900 nM for TgSkp1ΔCTR, which folds well (Figures 4B, S4), to an estimated 50 nM for full-length TgSkp1 was observed. Remarkably, a randomly scrambled sequence of the CTR served nearly as well, lowering the apparent affinity to a similar value as full-length TgSkp1. However, lesser success was achieved with another scrambled sequence and an equal length poly-Ser sequence rendered no improvement.

Studies on TgSkp1Δloop, which replaces 12 internal-disordered amino acids with a GGSG tetrapeptide, established a contribution by this region based on an increase of the *K*_d_ to 200 nM. Deletion of the CTR in this construct (TgSkp1ΔloopΔCTR) raised the *K*_d_ to 2400 nM. This confirms the contribution of the CTR where all *K*_d_ values rose to within the range that can be directly measured by absorbance in the AUC. The results also showed that the contribution of the CTR was at least partly independent of the internal disordered loop. By raising the ionic strength of the solution, the contribution of the CTR to affinity was abolished while having a minimal effect on the ordered interaction site. This is consistent with the predominantly hydrophobic nature of Skp1’s ordered homodimer interface (subsite-A) and is the basis for concluding that the CTR-CTR interaction serves as subsite-B (Figure 8).

**Figure 8:**
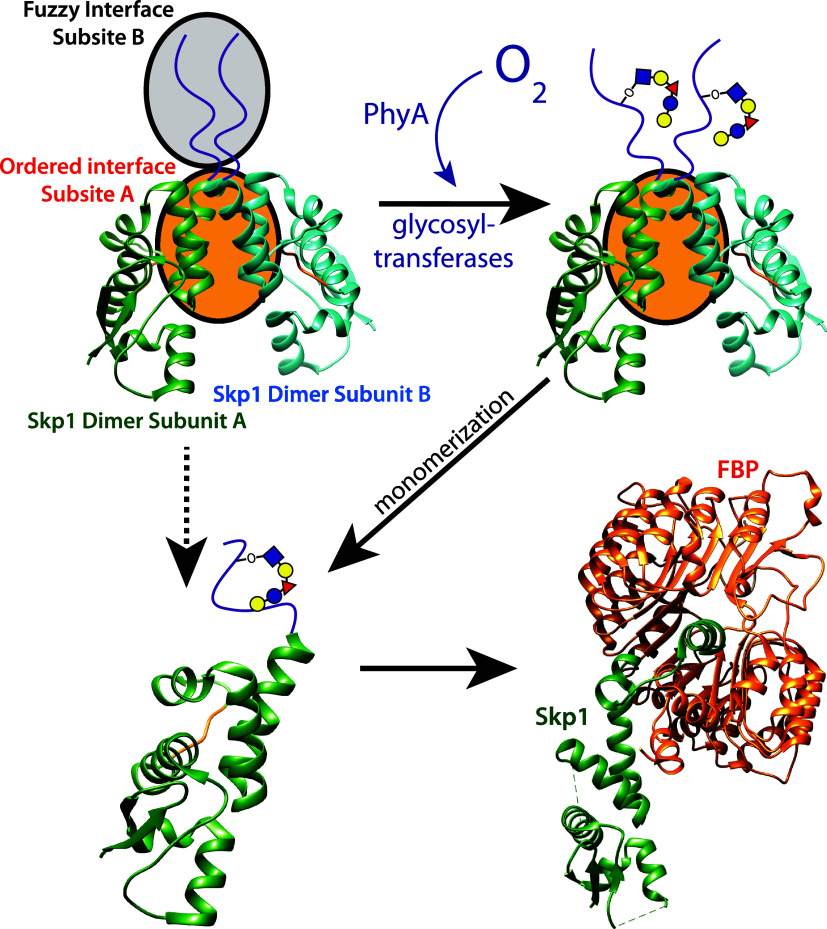
Evidence supports a bipartite mechanism for Skp1 homodimerization. Subsite-A includes the stably ordered α-helices-5 and -6, and subsite-B includes the intrinsically disordered CTR. Subsite A buries ∼750 Å^2^ of predominantly hydrophobic surface, whereas subsite-B involves a fuzzy interaction characterized primarily by dynamic electrostatic interactions that are hindered by the glycan. The dynamic nature of the CTR may facilitate access to the posttranslational modification enzymes and exchange of a Skp1 monomer for an FBP, and O_2_-dependent glycosylation is expected to promote accessibility of monomeric Skp1 to F-box proteins (FBPs), as represented by the solid vs dashed vertical or diagonal arrows. Individual DdSkp1 subunits are from PDB ID: 6V88 while the Skp1-FBP interaction is from PDB ID: 2P1O (Skp1-like protein 1A and Tir1 from A. thaliana).

Modeling with all-atom MD utilizing explicit water provided support for frequent, transient and varying interactions between oppositely charged residues across the two CTRs, whose proximity is dictated by the ordered interface. Energy calculations showed enhanced contributions between clustered charge regions and, in some instances, neighboring hydrophobic contacts. This corresponds to a definition of charge-based fuzzy protein interactions consisting of electrostatically facilitated protein–protein interactions mediated by an ensemble of bound conformations.^23–25^ This contrasts with hydrophobically facilitated fuzzy protein interactions such as those made by Gcn4 where many weak hydrophobic contacts mediate binding.^72^ The different contributions of the replacement CTR sequences to homodimer affinity is consistent with a fuzzy interaction. In contrast to the native and Scrambled6 sequences, the less effective Scarmbled5 sequence lacked directly paired positively charged residues, which have been shown to influence the properties of biomolecular condensates.^59,60,73,74^ The inertness of the poly-Ser replacement is consistent with the importance of Coulombic interactions rather than entropy being a driving force.

Charge-based fuzzy protein interactions are an interesting recent development in understanding the functions of intrinsically disordered proteins and protein segments, and can be mechanistically related to liquid–liquid phase separation.^66^ The paradigm of charge based fuzzy interactions of disordered proteins has mostly been applied to the association of two oppositely charged regions with lesser contributions from charge distribution.^58,66^ This can allow collapse of extended protein segments into a population of dynamically interconverting conformers, resulting in an increase in conformational entropy.^58^ Reliance of the conformational collapse on polar and ionic interactions makes these fuzzy protein interactions particularly sensitive to salt concentration, as observed here. Despite the term ‘fuzzy interaction’ being more typically applied to the interaction of dissimilar sequences, here we propose that this term can also apply to the homodimerization of two identical intrinsically disordered segments and not depend on net charge differences to be a primary driving factor.

The utilization of the same sequence for two independent binding mechanisms, i.e., to FBPs and to itself, imposes critical constraints on the nature and evolution of both. It is proposed here that the fuzzy component for homodimerization has characteristics that are highly suitable for this dual activity. The interface with FBPs is striking in its high expected affinity toward all of the dozens or more of predicted FBPs in organisms with a single Skp1, despite the low sequence identity of their F-box domains. Crystallographic studies highlight the involvement of α-helices 5–7 as part of the core interaction, termed subsite-1 here, and α-helix-8, as contributing a dispensable variable interaction, here termed subsite-2 (Figure 8). While subsite-1 is sufficient for binding, the inclusion of subsite-2 increases the half-life of the complex from hours to days for human Skp1.^18^ Whereas subsite-A of self-interaction involves α-helices 5–6 (which bear no resemblance to F-box sequences), subsite-B involves the CTR, which remains disordered as a fuzzy interaction. The relaxed sequence specificity of the fuzzy interaction is predicted to have been compatible with evolutionary optimization of binding of the CTR to F-box domains. Furthermore, the different binding mechanisms between FBPs and self-may allow for differential evolutionary tuning of the strength of the homodimer. For example, charged residues are important for fuzzy interactions but typically less conserved and less important for the formation of ordered dimer interfaces such as for FBP/Skp1 interactions. The identity of positive charge residues has also been observed to alter dynamic interactions with Arg residues contributing disproportionately to these interactions compared to Lys residues.^75–81^ Evolutionary variation of charged residues within the Skp1 CTR may therefore offer a selective way to influence the strength of the Skp1 homodimer. This may explain differences in Skp1 homodimerization affinities in different organisms^14,20^ that may be independent of FBP/Skp1 interactions. Additionally, alteration of charges through phosphorylation has been shown to alter the propensity for liquid–liquid phase separation.^74^ The previously observed phosphorylation of the CTR of yeast Skp1 ^82^ may, therefore, influence its homodimerization too.

The presence of a fuzzy component to the homodimer interface may facilitate kinetic exchange between the homodimer, FBPs, and Skp1’s modification enzymes. Charge-based fuzzy interactions can drive higher on and off rates for dimer subunits^25,66,83,84^ and, in the case of Skp1, may provide an opportunity for invasion of the F-box domain. Once bound, the F-box binding interface will involve α-helices-5 and −6 that contribute to the ordered homodimerization subsite-A. The CTR of the fuzzy homodimerization subsite-B almost always folds into an α-helix pair, with helix-8 overlapping with the previously described subsite-2 of the FBP interface.^18^ The fully ordered interaction of the CTR with FBPs may be less susceptible to dissociation. Fuzzy interactions are thought to be more receptive to posttranslational modifications,^25,66,83,84^ which in the case of Skp1 might facilitate O_2_-sensing via more ready access to the CTR by PhyA and the glycosyltransferases, whose actions are inhibited in complexes with FBPs.^35^

Glycosylation of Skp1’s CTR resulted in a significant weakening of the Skp1 homodimer by over an estimated order of magnitude, mirroring the effect of deleting Skp1’s CTR. The simplest interpretation is that glycosylation negates the CTR’s contribution to homodimerization. Previous studies of both TgSkp1 and DdSkp1 suggested that the glycan has a relatively ordered structure that associates with and therefore partially orders the region of α-helix-7 and the connecting loop to the start of the α-helix-8 region.^21,34,69^ This interaction would interfere with the dynamic contacts of charge clusters that form the basis of the fuzzy interaction, and deserves future analysis.

The estimated 50 nM *K*_d_ value that describes the homodimer interaction of unmodified Skp1 implies that the vast majority of Skp1, estimated in mammalian cells to be 1–2 μM,^85^ will be in the dimer state. However, a substantial fraction of Skp1 will be complexed at a 1:1 ratio with FBPs, which for a single example has been characterized to have a *K*_d_ of ∼ 25 nM.^22^ Since the interaction sites for FBPs and Cul1 overlap with the Skp1 homodimer interface (Figure S18), most free Skp1 will be sequestered from the SCF complex. Under normoxic conditions, hydroxylation and subsequent glycosylation of Skp1’s CTR will disrupt the fuzzy dimerization interface resulting in less sequestration of Skp1 into the homodimer species. Under hypoxic conditions the fuzzy interface will remain intact driving greater sequestration of Skp1 into the homodimer species (Figure 8). This would theoretically result in a reduced polyubiquitination activity for substrates of FBPs reliant on the SCF complex especially for lower affinity FBP/Skp1 species. This parallels the role of animal PHD2 where reduced polyubiquitination of HIF1α results in the activation of the hypoxic response.^86^

The varied sequences of FBPs that associate with the single Skp1 suggests the existence of an affinity hierarchy. A consequence of the destabilizing effect of glycosylation might be to reduce the significance of FBP preferences. For example, weakening of the Skp1 homodimer will exert a stronger associative effect on relatively weak FBP/Skp1 interactions while having a relatively minimal effect for high affinity FBPs that already had an advantage. This effect can also be extended to include a concentration hierarchy where higher abundance FBPs will be able to more effectively outcompete a high affinity Skp1 homodimer. This would allow variations in FBP transcription to influence the tuning of FBP/Skp1 interactions by homodimer sequestration that is in turn responsive to physiological O_2_ levels.

It has been previously estimated that 40% of proteins encoded in the human genome have an intrinsically disordered protein segment greater than 30 residues in length.^87–90^ Many of these participate in liquid–liquid phase separation,^71^ fuzzy protein interactions,^71^ chaperoning,^91^ altering internal protein dynamics,^92,93^ and tuning protein function through entropic force generation,^64^ though most have no known function. Here, we suggest that our interpretation that a fuzzy interface consisting of identical charge-rich sequences can promote homodimerization offers a new functional dimension for interactions of intrinsically disordered regions.

## Conclusions

Quantitative measurements of the homodimerization *K*_d_ of Skp1 from the protozoan parasite T. gondii, using analytical ultracentrifugation, emphasize its potential impact on the activities of E3­(SCF) ubiquitin ligases in cells. The relatively high (multi-nM) affinity of its self-interaction is likely to compete with the formation of Skp1/FBP subcomplexes by a sequestration mechanism. Both interfaces are bipartite consisting of a stably folded Skp1 domain and the CTR. Whereas the CTR folds into a stereotyped pair of α-helices when bound to FBPs, it participates in a largely charged-based fuzzy interaction involving ensembles of conformations in the homodimer. Fuzzy interactions have been implicated in formation of biomolecular condensates but have rarely been invoked for homodimerization. Thus, the CTR contributes substantial affinity to the homodimer but its intrinsically dynamic nature is predicted to also help enable exchange with FBPs. The weakening of the CTR/CTR interaction by O_2_-dependent glycosylation, which may impose local order to the CTR, suggests that sequestration of Skp1 from FBPs is regulated in cells. Previous studies suggested that the glycan also promotes favorable conformations for binding FBPs. The alternative organization of the CTR in the different complexes imposes unusual constraints on the evolution of its sequence, which is remarkably well conserved across eukaryotic evolution. Although glycosylation appears to be confined to the protist kingdom and pathogenic fungi, homodimerization of Skp1 and its regulation by other mechanisms is likely to be a widespread mechanism to influence the activities of the SCF family of E3 ubiquitin ligases.

## Supplementary Material



## Notes

Sedimentary velocity data, circular dichroism data, thermal melting data, molecular dynamics simulations, and sequence maps for generating protein variants (PDF)

## #

Helix BioStructures, Indianapolis, Indiana 46241, United States

## Abbreviations

AUC: analytical ultracentrifugation
CD: circular dichroism
CTR: C-terminal region
DdSkp1: Skp1 from Dictyostelium discoideum
GGlFGGn-Skp1: fully glycosylated Skp1, with a GalGlcFucGalGlcNAc-glycan at Hyp154
MALDI-TOF-MS: matrix assisted laser desorption ionization time-of-flight mass spectrometry
MD: molecular dynamics
Skp1-FL: full-length Skp1 with a Ser-Met extension N-terminal to the normal Ser N-terminus
TgSkp1: Skp1 from Toxoplasma gondii
