# Supplementary material for: Glycosylation Weakens Skp1 Homodimerization in Toxoplasma gondii by Interrupting a Fuzzy Interaction
Source: Biochemistry. 2025 Apr 29;64(10):2262–79. doi: 10.1021/acs.biochem.4c00859 (PMC12101541; doi:10.1021/acs.biochem.4c00859)
Supplement: Supplementary file 1 [file bi4c00859_si_001.pdf]

SUPPLEMENTARY INFORMATION

Glycosylation Weakens Skp1 Homodimerization in *Toxoplasma gondii* by Interrupting a Fuzzy Interaction

Donovan A. Cantrell[1], Ramona J. Bieber Urbauer[1,2], Hyun W. Kim[1,3], Robert J. Woods[1,3], Jeffrey L. Urbauer[1,2], Zachary A. Wood[1], Christopher M. West*[,1,2,4,5]

[1]Department of Biochemistry and Molecular Biology, [2]Department of Chemistry, [3]Complex Carbohydrate Research Center, [4]Center for Tropical and Emerging Diseases, [5]Center for Molecular Medicine, University of Georgia, Athens, GA 30602 USA

* To whom correspondence should be addressed: Email, westcm@uga.edu
# Present address for H.W.K.: Helix BioStructures, Indianapolis, IN 46241, United States

Table of Contents

The 20 supplementary figures include additional sedimentary velocity data, circular dichroism data, thermal melting data, molecular dynamics simulations, and sequence maps for generating protein variants.

Figure S1. c(s) distributions over a range of TgSkp1-FL concentrations
Figure S2. c(s) distributions over a range of GalGlcFucGalGlcNAc-TgSkp1 concentrations
Figure S3. c(s) distributions over a range of TgSkp1ΔCTR concentrations
Figure S4. Thermal melting studies of TgSkp1 variants
Figure S5. Integration ranges for TgSkp1-Scrambled5, TgSkp1-polySer, TgSkp1ΔLoop, and TgSkp1ΔLoopΔCTR
Figure S6. c(s) distributions over a range of TgSkp1-Scrambled6 concentrations
Figure S7. c(s) distributions over a range of TgSkp1-Scrambled5 concentrations
Figure S8. c(s) distributions over a range of TgSkp1-polySer concentrations
Figure S9. c(s) distributions over a range of TgSkp1ΔLoop concentrations
Figure S10. c(s) distributions over a range of TgSkp1ΔLoopΔCTR concentrations
Figure S11. c(s) distributions over a range of TgSkp1ΔLoop concentrations in high salt
Figure S12. c(s) distributions over a range of TgSkp1ΔLoopΔCTR concentrations in high salt
Figure S13. Total residue energy contributions across simulations mapped to TgSkp1 homology models
Figure S14. Total residue energy contributions averaged across all simulations
Figure S15. Fuzzy CTR interactions for Simulation 2
Figure S16. Fuzzy CTR interactions for Simulation 3
Figure S17. Distances between interacting charge clusters through the MD simulations
Figure S18. Overlaps between Skp1 homodimer and adjacent SCF components
Figure S19. Engineering TgSkp1 deletions
Figure S20. Sequences of chemically synthesized variants of TgSkp1 genes

Figure S1. c(s) Distributions over a range of TgSkp1-FL concentrations. Samples were monitored at the indicated wavelengths. Buffer components were identical for all standard runs (50 mM KPhosphate (pH 7.4), 25 mM KCl), with an ionic strength of ~150 mM. Predicted S values for the monomer as predicted by homology modeling (purple) and the dimer as predicted by MD Cluster analysis (brown) are shown. The data support Figure 3.

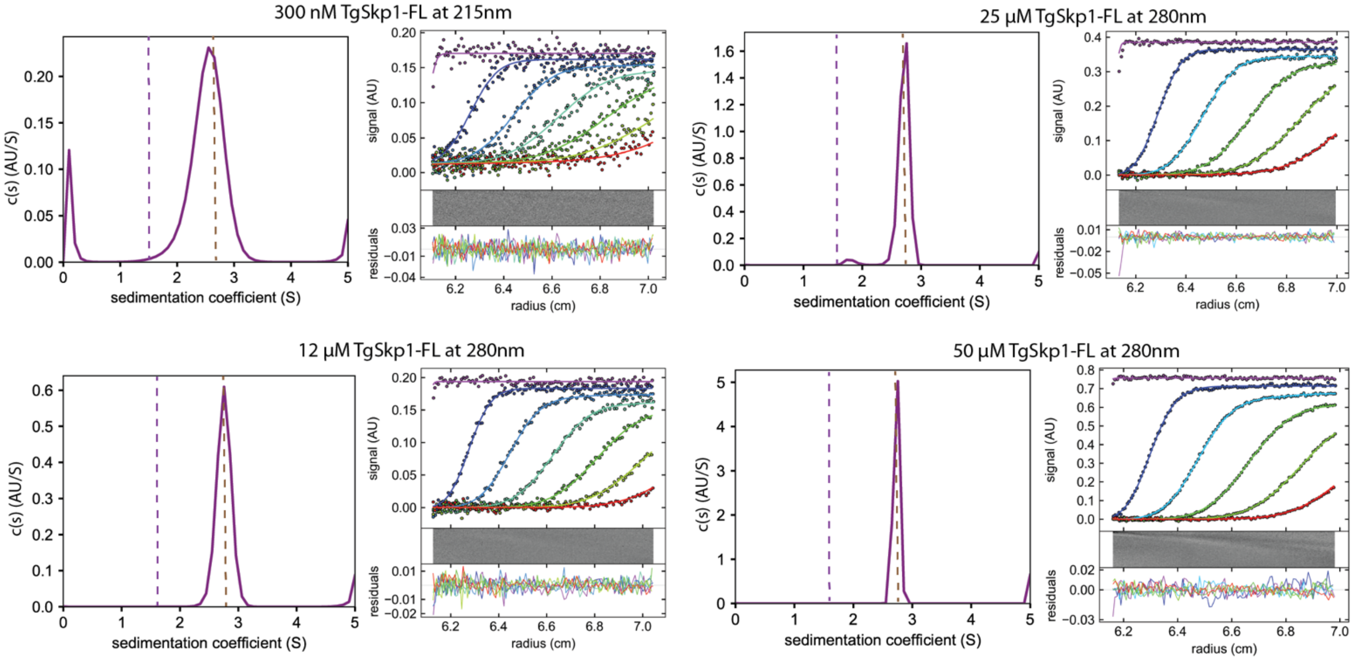

Figure S2. c(s) distributions over a range of GalGlcFucGalGlcNAc-TgSkp1 concentrations. Studies were performed as in Figure S1. The replicate distribution of 330 nM GalGlcFucGalGlcNAc-TgSkp1 boxed in red more clearly shows the monomer and dimer species but was not used in generating the S_w isotherm. The data support Figure 3.

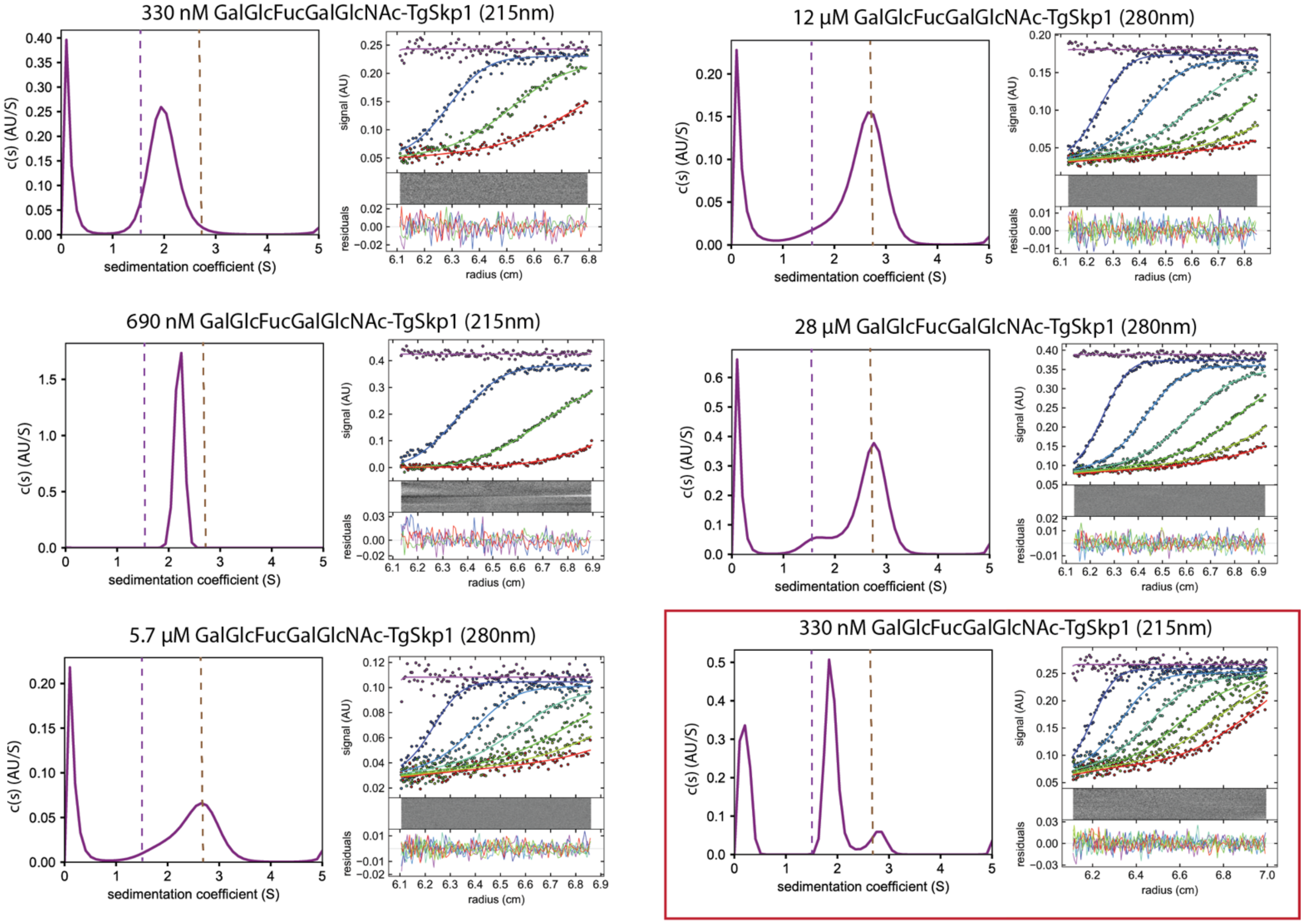

Figure S3. c(s) distributions over a range of TgSkp1ΔCTR concentrations. Studies were performed as in Figure S1 except that homology modeling was used to predict the monomer (purple) and dimer (green) S values. The data support Figure 4.

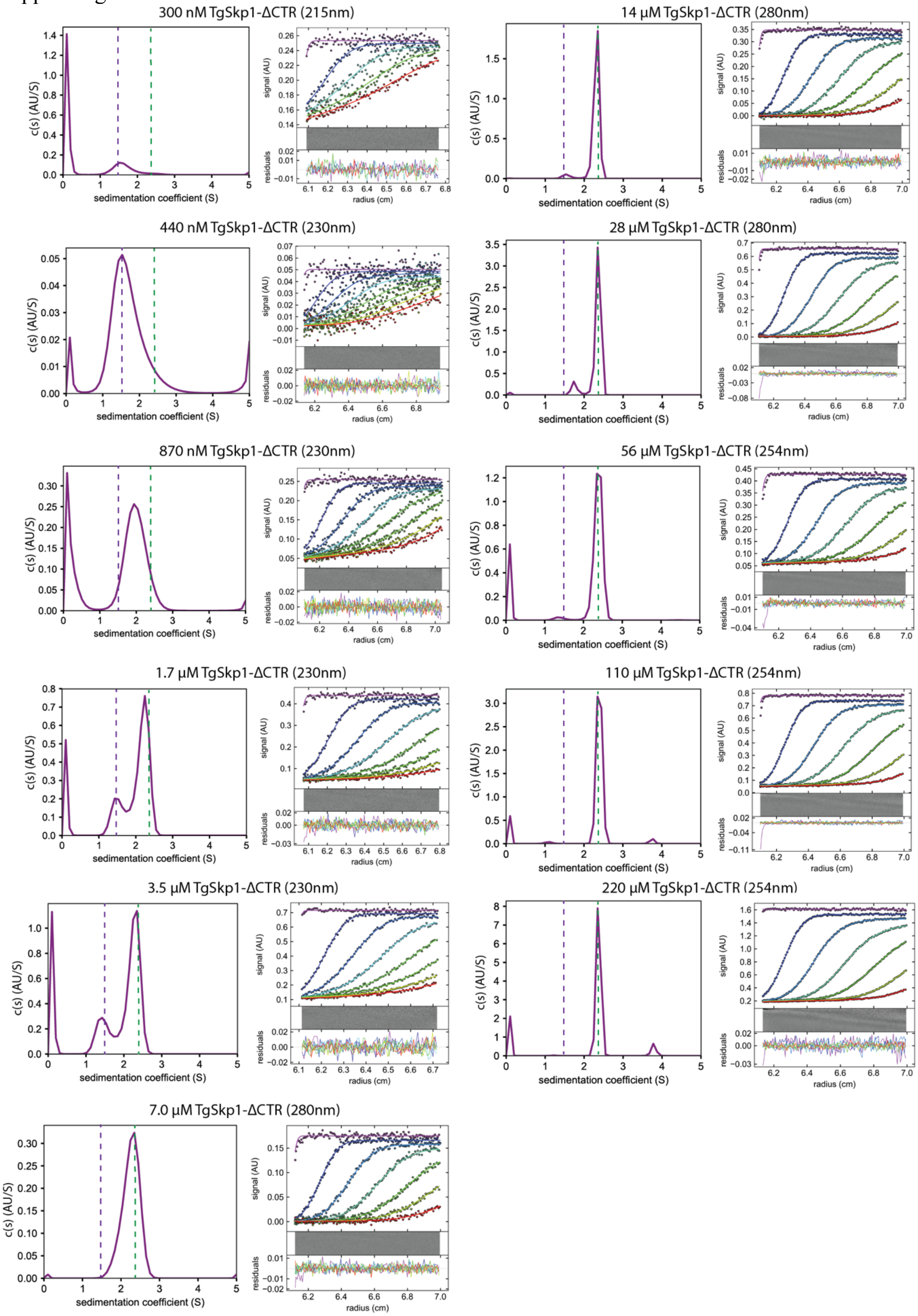

Figure S4. Thermal melting studies of TgSkp1 variants. Forward (black) and reverse (red) thermal melts for TgSkp1-FL (A), TgSkp1-ΔCTR (C), TgSkp1-Scrambled6 (E), TgSkp1-Scrambled5 (G), and TgSkp1-PolySer (I) were modeled to the Gibbs-Helmholtz equation, with T_m and ΔH values indicated. CD spectra for the beginning (black), max temperature (blue), and end (red) of each thermal melt for TgSkp1-FL (B), TgSkp1-ΔC-term (D), TgSkp1-Scrambled6 (F), TgSkp1-Scrambled5 (H), and TgSkp1-PolySer (J) are shown, and their secondary structure contents were modeled (K).

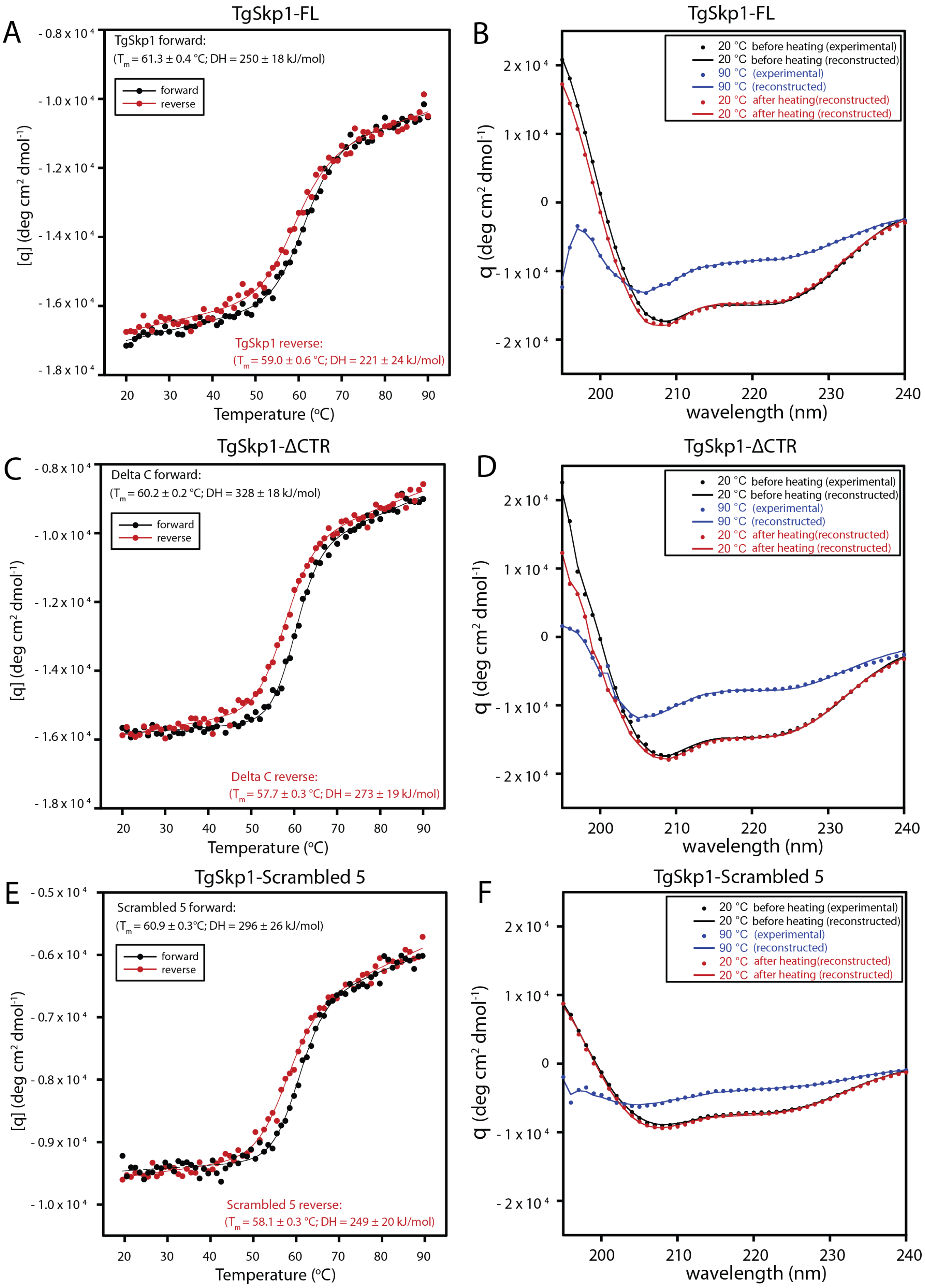

G

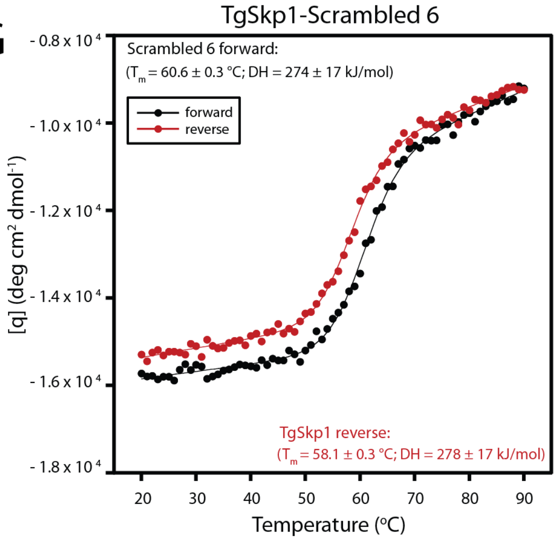

H

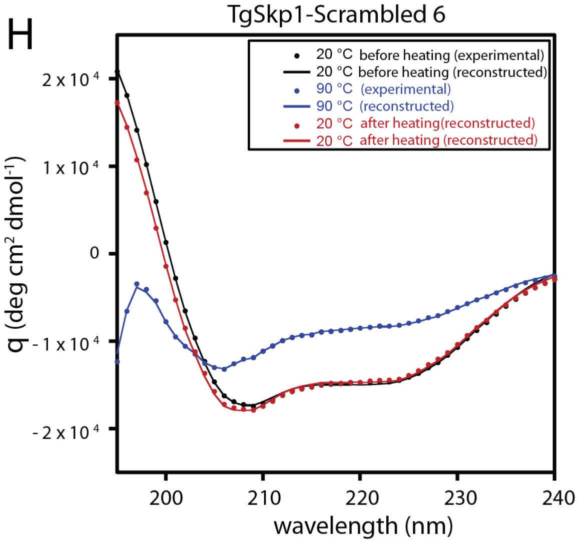

I

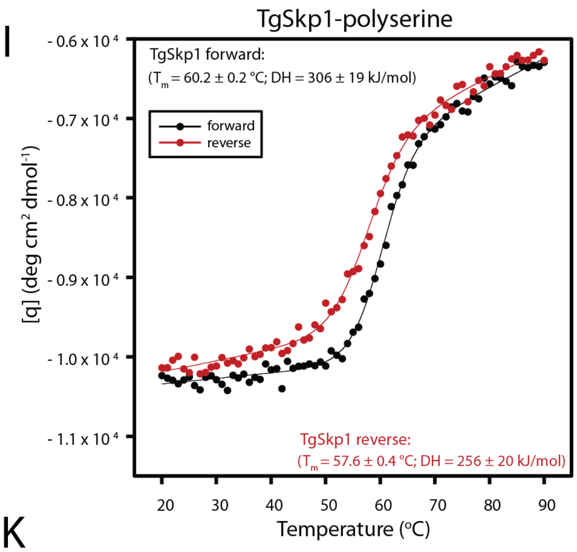

J

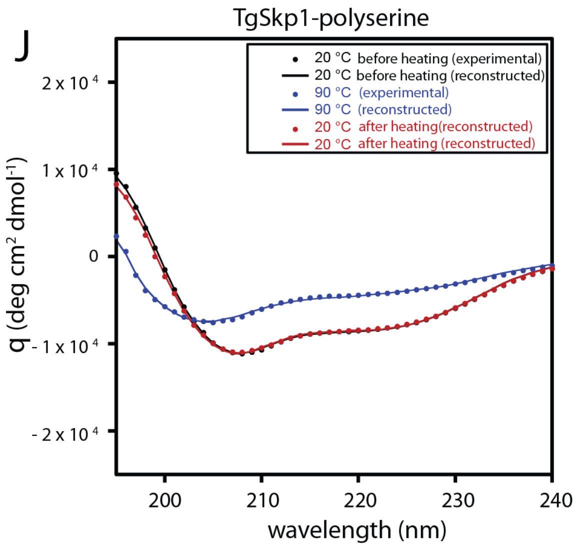

K

Sample	α-helix (%)	β-strand (%)	β-turns (%)	Unordered (%)
TgSkp1-FL (before 20 °C)	52	7	14	27
TgSkp1-FL (90 °C)	38	21	14	28
TgSkp1-FL (after 20 °C)	56	7	13	23
TgSkp1-ΔC terminus (before 20 °C)	57	10	12	19
TgSkp1-ΔC terminus (90 °C)	35	11	16	38
TgSkp1-ΔC terminus (after 20 °C)	56	10	14	21
TgSkp1-Scrambled 6 (before 20 °C)	49	10	14	28
TgSkp1-Scrambled 6 (90 °C)	16	13	13	58
TgSkp1-Scrambled 6 (after 20 °C)	50	9	17	24
TgSkp1-Scrambled 5 (before 20 °C)	22	23	20	35
TgSkp1-Scrambled 5 (90 °C)	5	28	18	47
TgSkp1-Scrambled 5 (after 20 °C)	25	19	28	36
TgSkp1-polyserine (before 20 °C)	32	13	18	37
TgSkp1-polyserine (90 °C)	7	29	27	46
TgSkp1-polyserine (after 20 °C)	32	11	16	41

Figure S5. Integration ranges used to generate c(s) isotherms. (A-F) Distributions and integration ranges are shown for the indicated constructs at the indicated salt concentrations.

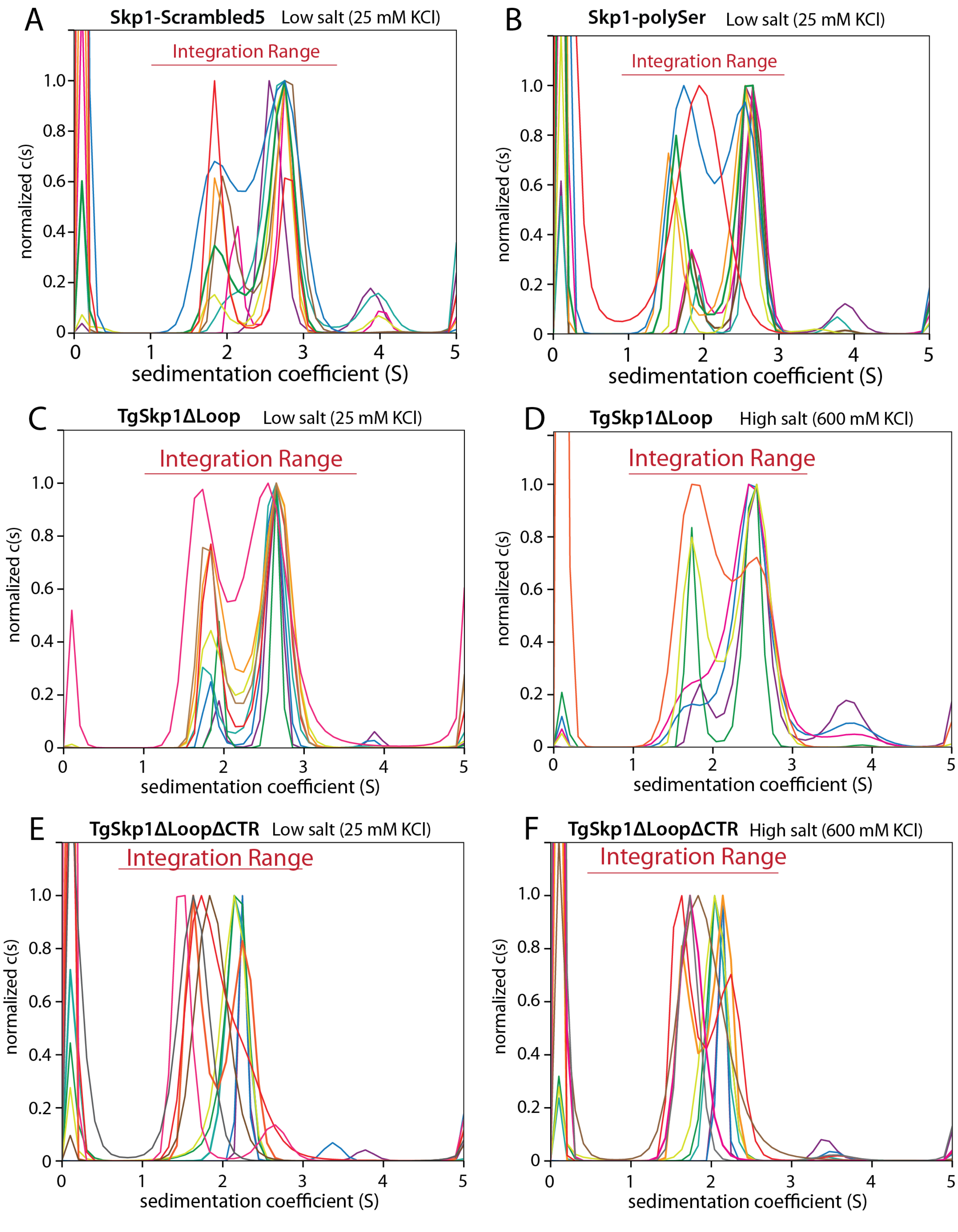

Figure S6. c(s) distributions over a range of TgSkp1-Scrambled6 concentrations. Studies were performed as described in Figure S1. The data support Figure 5.

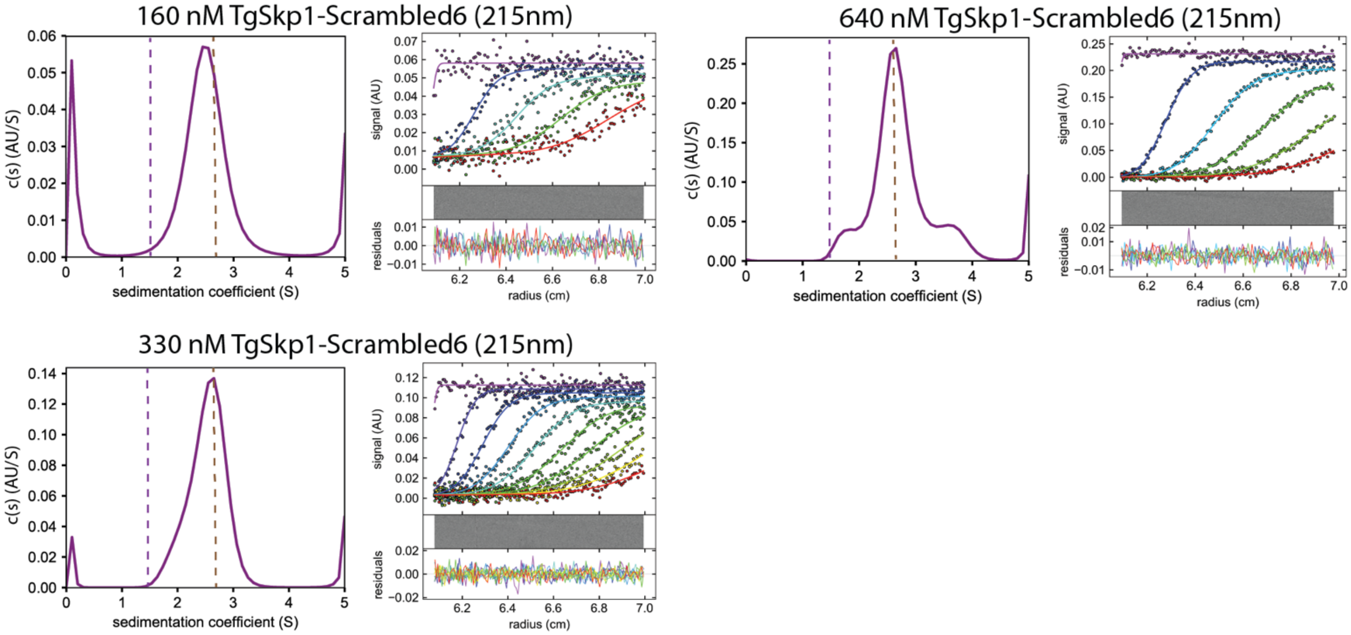

Figure S7. c(s) distributions over a range of TgSkp1-Scrambled5 concentrations. Studies were performed as in Figure S1. The data support Figure 5.

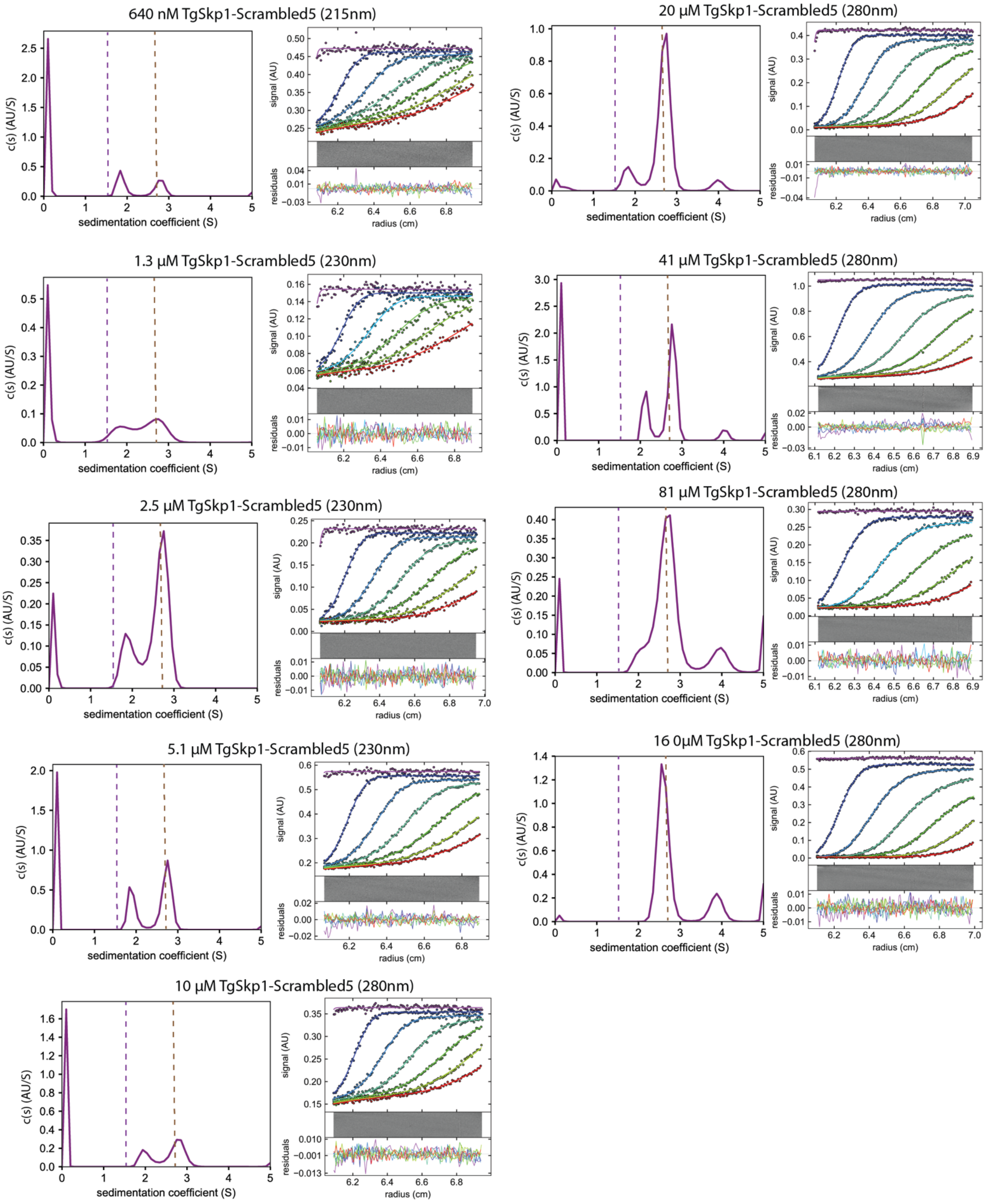

Figure S8. c(s) distributions over a range of TgSkp1-polySer concentrations. Studies were performed as in Figure S1. The data support Figure 5.

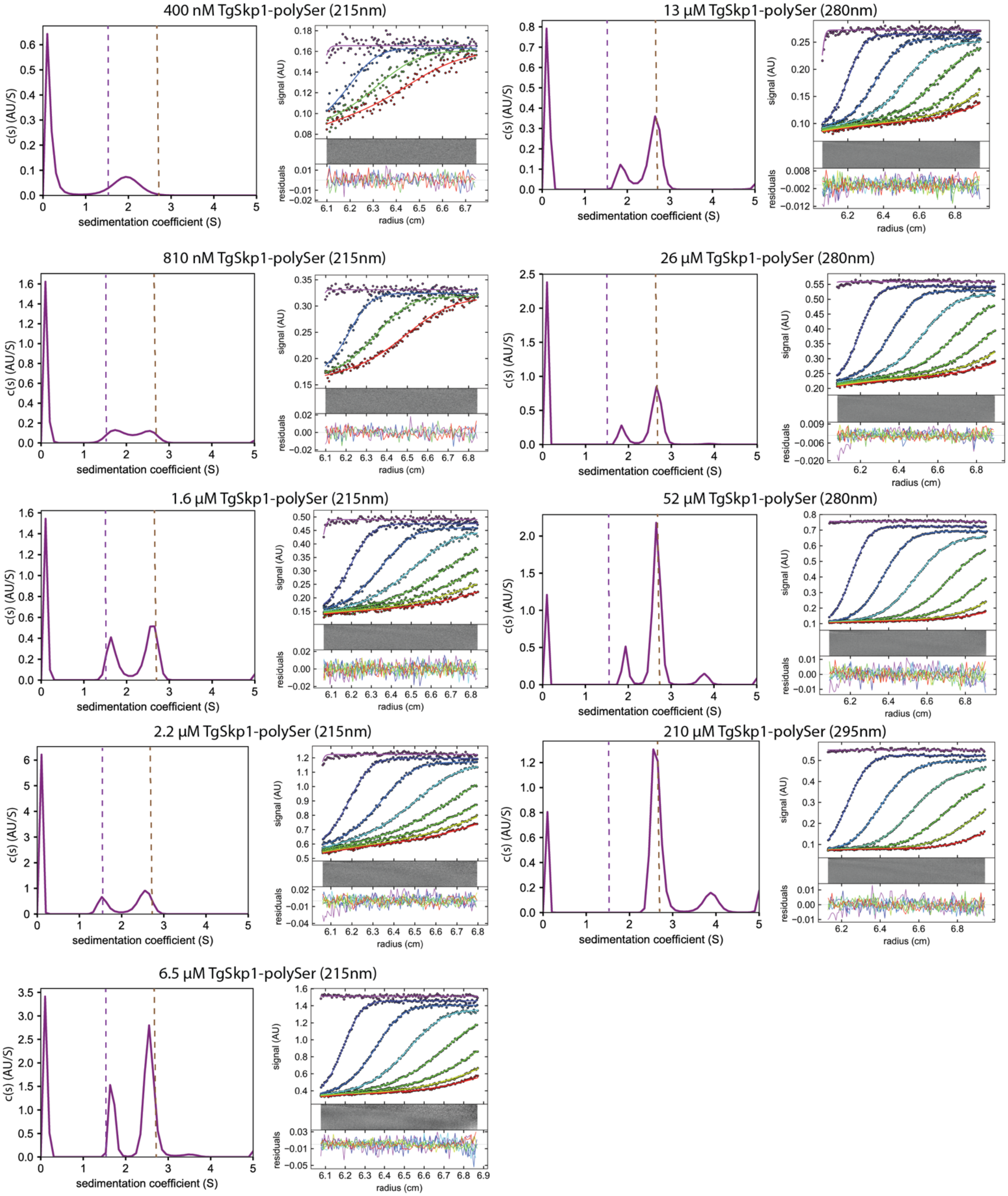

Figure S9. c(s) distributions over a range of TgSkp1ΔLoop concentrations. Studies were performed as in Figure S1. The data support Figure 6.

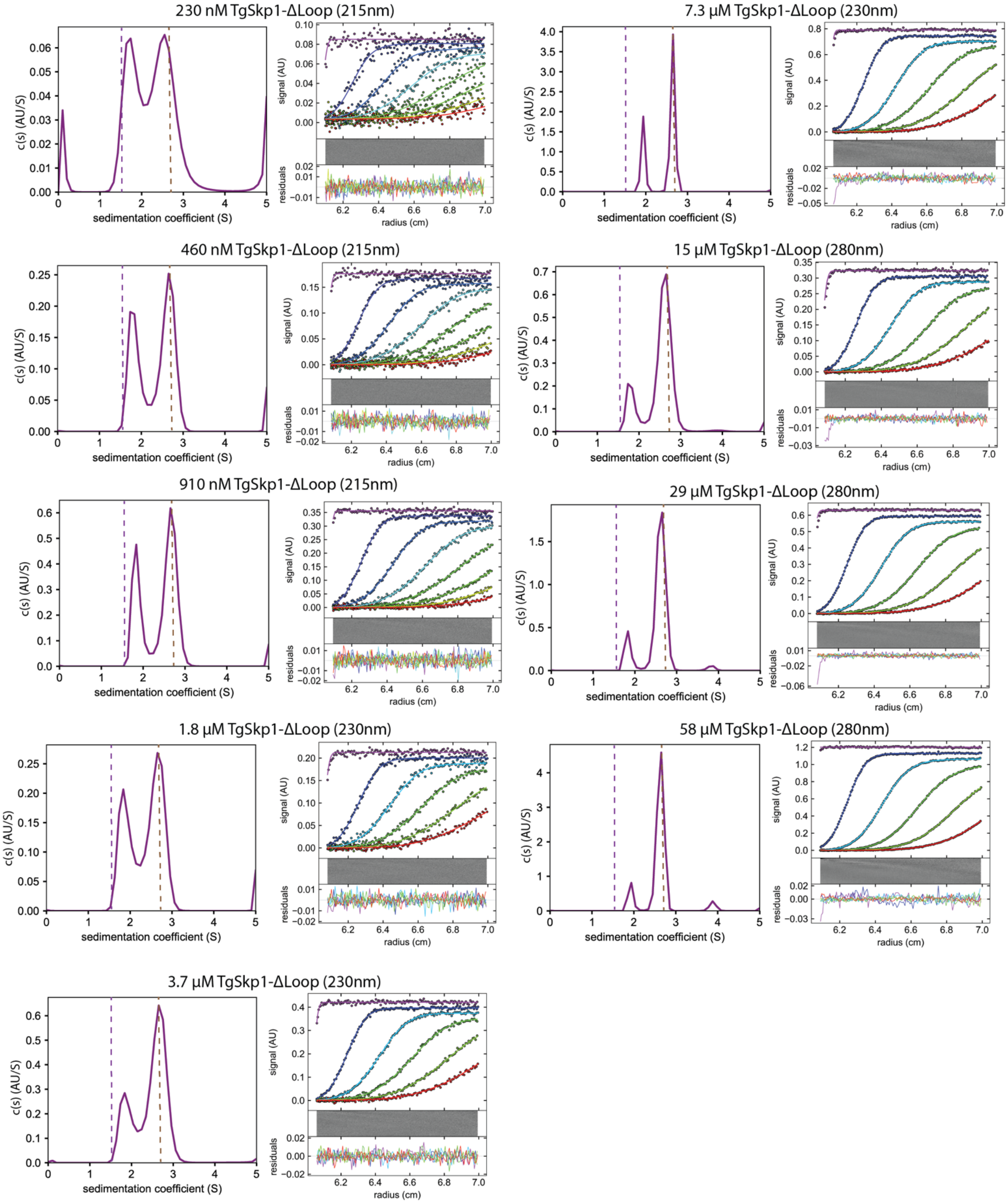

Figure S10. c(s) distributions over a range of TgSkp1ΔLoopΔCTR concentrations. Studies were performed as in Figure S3. The data support Figure 6.

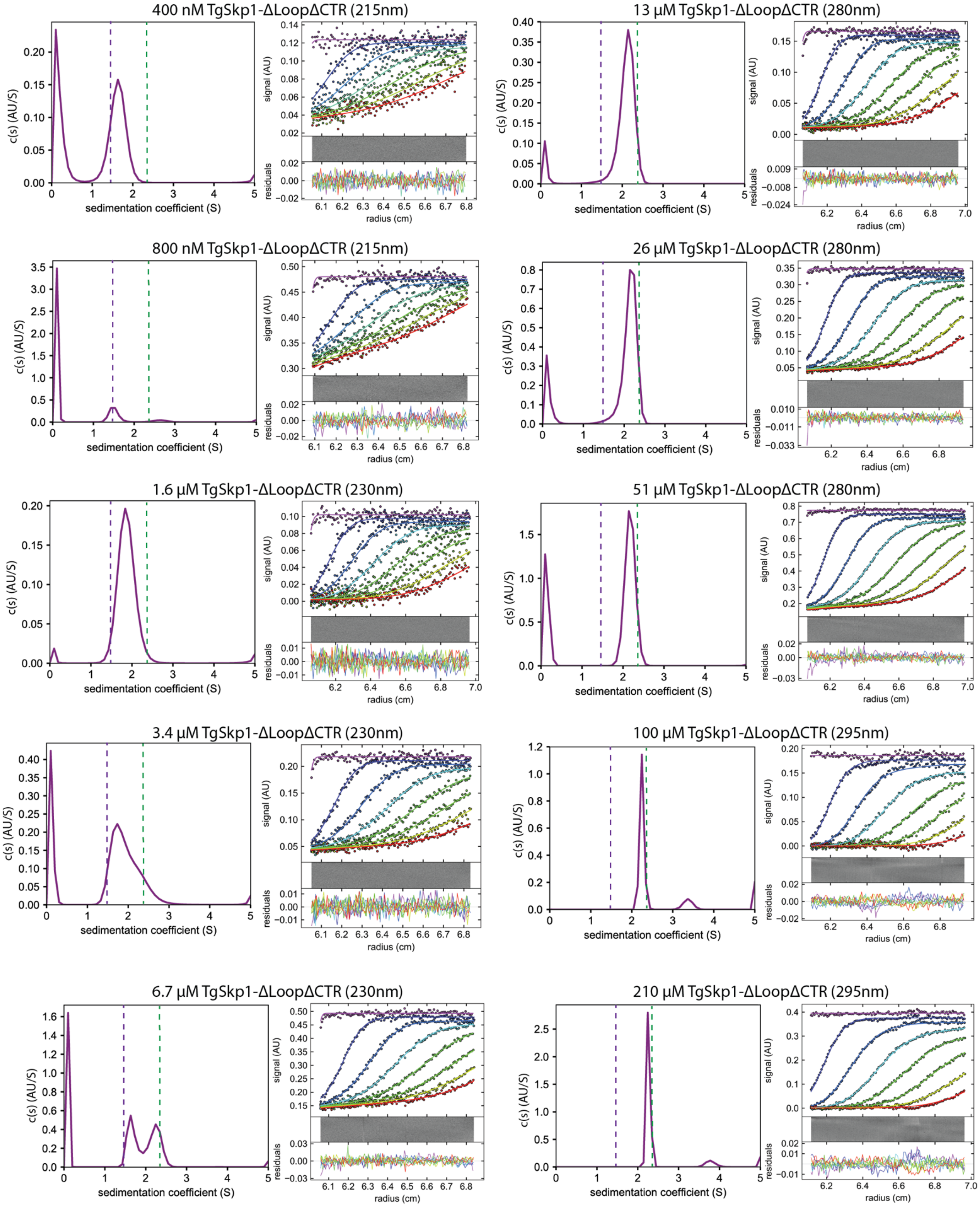

Figure S11. c(s) distributions over a range of TgSkp1ΔLoop concentrations in high salt. Studies were performed as in Figure S1, except that buffer components were 50 mM K phosphate (pH 7.4), 600 mM KCl, with an ionic strength of ~730 mM. The data support Figure 6.

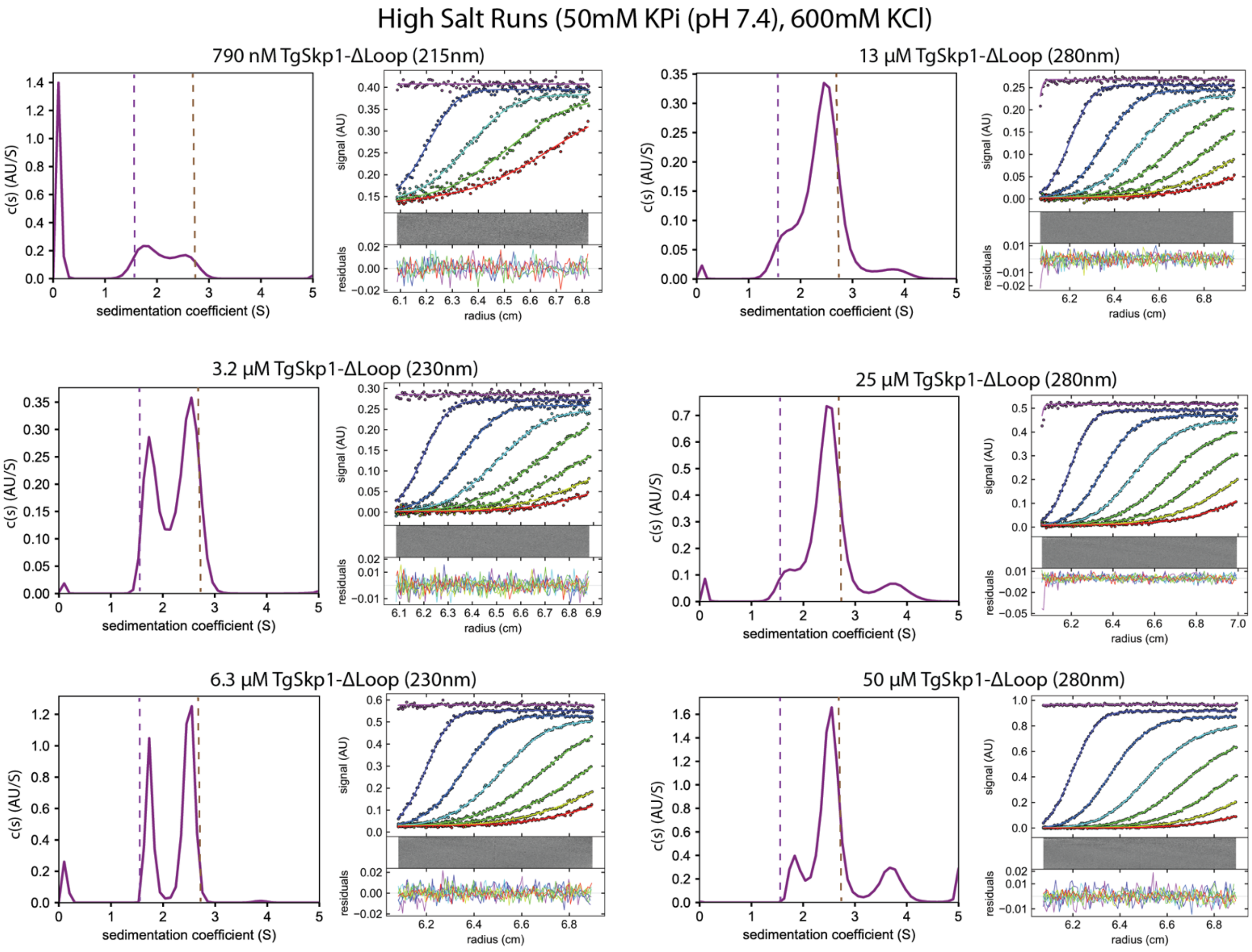

Figure S12. c(s) distributions over a range of TgSkp1ΔLoopΔCTR concentrations in high salt. Studies were performed as in Figure S11, except that homology modeling was used to predict the monomer (purple) and dimer (green) S values. The data support Figure 6.

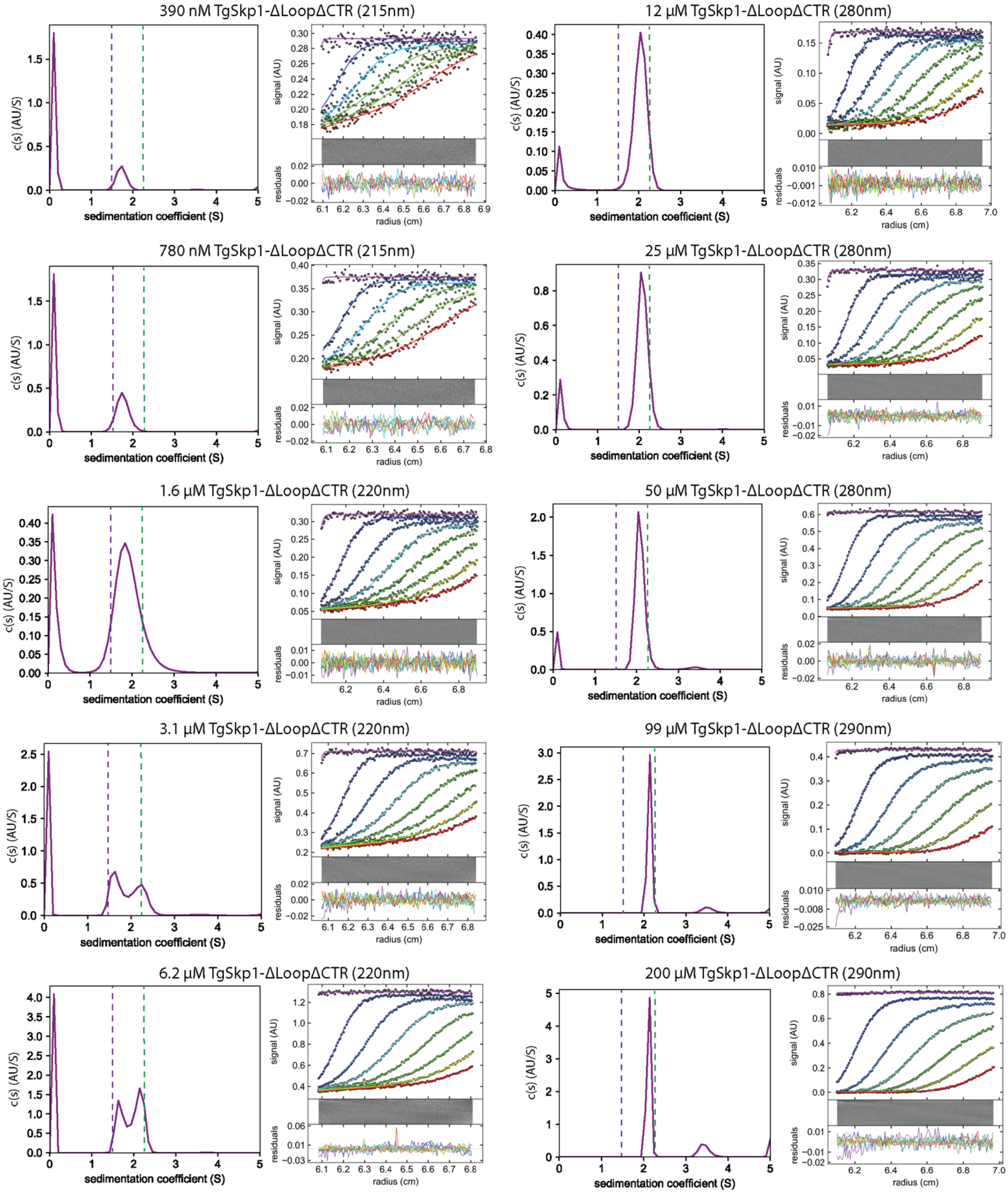

Figure S13. Total residue energy contributions across simulations mapped to TgSkp1 homology models. Total per residue energies for each simulation were mapped to their respective homology models. These energies were averaged together to yield the averaged per residue interaction energies and mapped to the starting homology model for simulation 1.

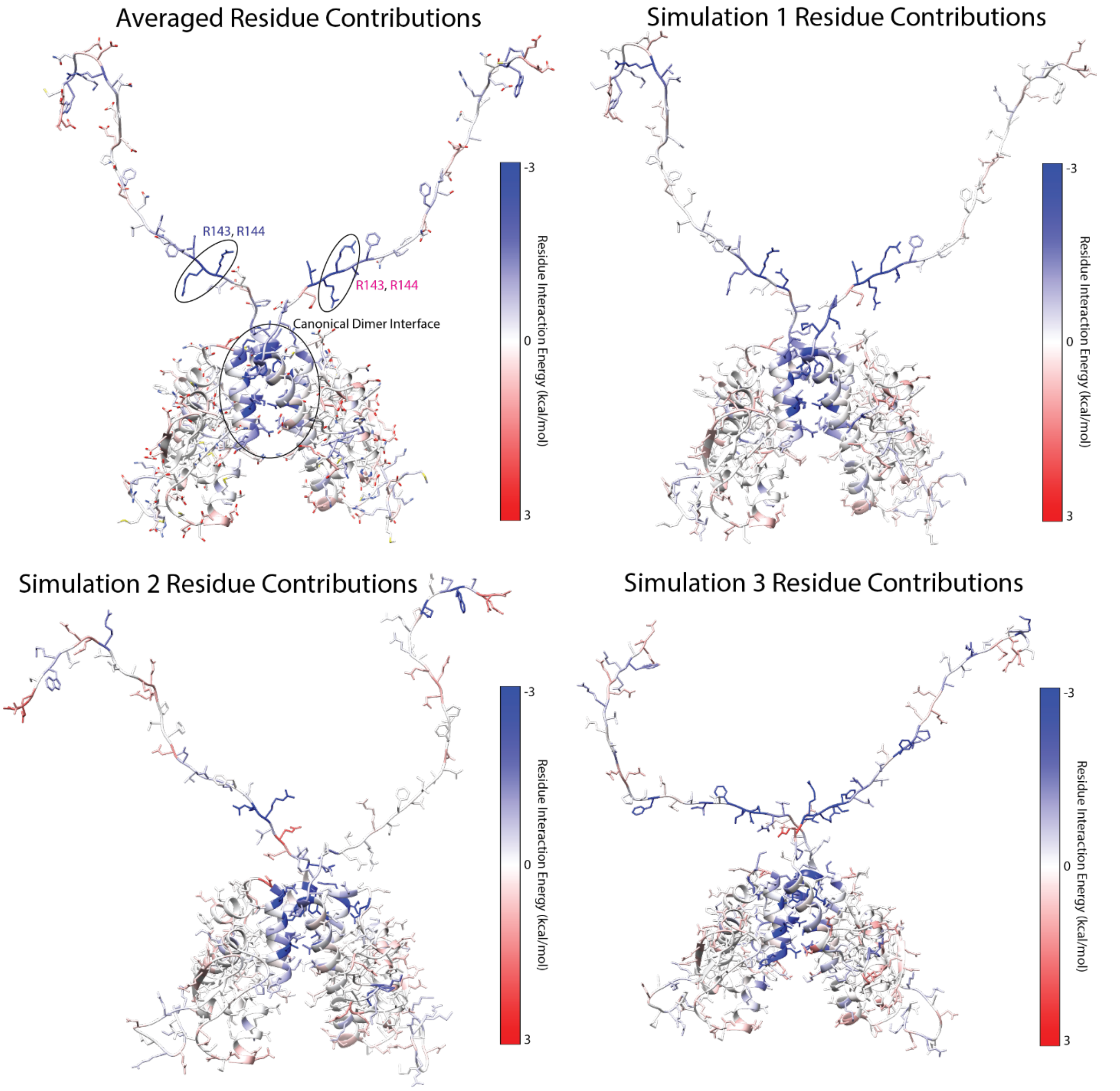

Figure S14. Total residue energy contributions averaged across all simulations. In tabular form, residues corresponding to subsite-A of the dimer interface are highlighted in light green. In the CTR, subsite B, positively charged residues are highlighted in light blue, negatively charged residues in light pink, and aromatic residues in yellow. These findings relate to Figures 7, S15, S16.

Dimer interface subsite-A

CTR Basic Residues

CTR Acidic Residues

CTR Aromatic Residues

Residue	Energy	Residue	Energy	Residue	Energy	Residue	Energy
M1	-0.63	D44	0.62	E88	0.89	A131	-0.63
S2	0.00	C45	0.00	V89	-0.22	T132	0.11
K3	-0.62	Q46	0.01	V90	-0.09	M133	-0.90
E4	0.61	E47	0.66	S91	-0.06	I134	-3.4
R5	-0.64	S48	0.03	E92	0.76	K135	-1.5
M6	0.00	I49	-0.05	W93	0.01	A136	-0.56
G7	0.00	P50	-0.02	D94	0.87	K137	-1.5
D8	0.61	L51	0.07	Y95	-0.05	T138	-0.79
A9	0.00	P52	-0.33	Q96	0.06	P139	-0.91
R10	-0.62	N53	-0.09	F97	0.01	E140	0.43
K11	-0.63	V54	0.01	I98	0.00	E141	0.55
V12	0.01	D55	0.59	N99	0.11	I142	-1.7
T13	0.01	T56	-0.01	E100	-0.40	R143	-4.0
L14	0.02	C57	-0.07	N101	0.17	R144	-5.1
V15	0.02	I58	-0.03	S102	-0.21	I145	-1.9
S16	0.05	L59	-0.01	D103	0.82	F146	-1.3
Q17	0.11	K60	-0.61	Q104	-0.98	N147	-0.45
E18	0.76	K61	-0.36	K105	-2.1	I148	-0.90
G19	0.01	I62	0.02	I106	-0.72	V149	-0.24
D20	0.71	I63	-0.01	L107	-0.10	N150	-0.23
E21	0.65	E64	0.75	F108	-3.9	D151	0.59
F22	-0.01	Y65	-0.01	A109	-0.72	F152	-1.1
D23	0.64	C66	-0.01	L110	0.07	T153	-0.28
V24	0.00	E67	0.68	I111	-1.3	P154	-0.35
D25	0.67	H68	0.01	L112	-3.0	E155	0.69
I26	0.01	H69	0.03	A113	-0.06	E156	0.65
E27	0.74	H70	0.01	A114	0.20	E157	0.42
V28	0.03	N71	0.00	N115	-1.6	A158	-0.29
A29	0.03	N72	0.02	Y116	-0.88	Q159	-0.23
S30	0.06	P73	0.02	L117	0.08	V160	-0.39
M31	0.07	P74	0.00	N118	0.50	R161	-1.3
S32	-0.01	E75	0.92	I119	0.00	E162	0.52
A33	0.03	E76	1.0	K120	0.15	E163	-0.03
L34	-0.32	P78	0.00	P121	-0.13	N164	-0.10
I35	-0.02	K79	-0.82	L122	0.16	K165	-1.2
K36	-0.70	P80	-0.67	L123	-1.3	W166	-1.4
T37	0.03	L81	-0.98	D124	0.37	C167	-0.26
M38	-0.11	K82	-1.0	L125	0.04	E168	1.1
V39	0.00	S83	-0.05	S126	0.23	D169	1.1
E40	0.77	S84	-0.19	V127	-2	A170	1.0
E41	0.75	N85	-0.44	A128	-0.17		
D42	0.67	L86	-1.1	K129	-0.27		
S43	0.00	A87	-0.47	V130	-1.2		

Figure S15. Fuzzy CTR interactions for Simulation 2. (A) Interaction energy matrix showing the pairwise interaction energies for each Skp1 CTR residue. (B) Examples of direct and indirect interactions of oppositely charged residues shown through atom distances. (C) Directly interacting residues are shown as sticks with their Van der Waals surfaces. See Figure 7 for explanation.

Energy (kcal/mol)

>3.0	3.0	2.5	2.0	1.5	1.0	0.50	0.00	-0.50	-1.0	-1.5	-2.0	-2.5	-3.0	<-3.0

A

		K137	T138	P139	E140	E141	I142	R143	R144	I145	F146	N147	I148	V149	N150	D151	F152	T153	P154	E155	E156	E157	A158	Q159	V160	R161	E162	E163	N164	K165	W166	C167	E168	D169	A170
		-0.12	0.00	0.00	0.11	0.13	-0.01	-0.16	-0.09	-0.03	0.00	0.00	0.00	0.00	0.00	0.13	0.00	-0.01	-0.01	0.09	0.12	0.16	-0.08	-0.64	-0.11	0.40	-2.2	-4.8	-0.17	-2.1	-4.5	-1.2	-0.86	-1.6	-0.90
-0.60	K137	0.07	0.00	0.00	-0.11	-0.11	0.00	0.13	0.14	0.00	0.00	0.00	0.00	0.00	0.00	-0.07	0.00	0.00	0.00	-0.08	-0.07	-0.09	0.00	0.00	0.00	0.13	-0.12	-0.17	-0.01	0.11	-0.06	0.00	-0.49	-0.26	-0.12
-0.76	T138	0.00	0.00	0.00	0.00	0.00	0.00	0.00	0.00	0.00	0.00	0.00	0.00	0.00	0.00	0.00	0.00	0.00	0.00	0.00	0.00	0.00	0.00	0.00	0.00	0.00	0.00	-0.02	0.00	0.00	-0.72	-0.02	-0.71	-0.03	-0.01
-0.46	P139	0.00	0.00	0.00	0.00	0.00	0.00	-0.02	0.00	0.00	0.00	0.00	0.00	0.00	0.00	0.00	0.00	0.00	0.00	0.00	0.00	0.00	0.00	0.00	0.00	0.00	-0.01	-0.03	0.00	-0.01	-0.82	-0.02	0.01	0.00	0.00
0.32	E140	-0.07	0.00	0.00	0.10	0.10	0.00	-0.16	-0.13	0.00	0.00	-0.01	0.00	0.00	0.00	0.08	0.00	0.00	0.00	0.09	0.08	0.11	0.00	0.00	0.00	-0.31	0.04	0.50	0.02	-0.16	0.01	0.00	0.13	0.11	0.10
0.22	E141	-0.06	0.00	0.00	0.08	0.08	0.00	-0.11	-0.09	0.00	0.00	0.00	0.00	0.00	0.00	0.06	0.00	0.00	0.00	0.07	0.07	0.08	0.00	0.00	0.00	-0.11	0.11	0.12	0.00	-0.11	-0.04	0.00	0.10	0.09	0.09
-0.69	I142	0.00	0.00	0.00	0.00	0.00	0.00	0.01	0.00	0.00	0.00	0.00	0.00	0.00	0.00	0.00	0.00	0.00	0.00	0.00	0.00	0.00	0.00	0.00	0.00	0.01	-0.01	-0.02	0.00	0.01	-1.3	-0.02	-0.02	-0.01	0.00
-3.3	R143	0.08	0.00	0.00	-0.11	-0.11	0.00	0.27	0.20	-0.01	-0.01	0.01	0.00	0.00	0.00	-0.09	0.00	0.00	-0.01	-0.10	-0.09	-0.11	-0.01	0.00	0.00	0.35	-1.0	-2.2	-0.16	0.12	-2.9	-0.09	-0.26	-0.16	-0.12
-5.4	R144	0.07	0.00	0.00	-0.09	-0.09	0.00	0.13	0.12	0.00	0.00	0.01	0.00	0.00	0.00	-0.08	0.00	0.00	0.00	-0.09	-0.09	-0.12	-0.01	-0.01	0.00	1.3	-3.2	-8.2	-0.05	0.11	-0.07	-0.01	-0.12	-0.11	-0.10
-0.23	I145	0.00	0.00	0.00	0.00	0.00	0.00	0.00	0.00	0.00	0.00	0.00	0.00	0.00	0.00	0.00	0.00	0.00	0.00	0.00	0.00	0.00	0.00	0.00	0.00	-0.01	-0.19	-0.01	0.00	-0.10	-0.11	-0.03	-0.01	0.00	0.00
-0.31	F146	0.00	0.00	0.00	0.00	0.00	0.00	0.00	0.00	0.00	0.00	0.00	0.00	0.00	0.00	0.00	0.00	0.00	0.00	0.00	0.00	0.00	0.00	0.00	-0.01	-0.01	-0.07	0.00	0.00	-0.05	-0.45	-0.02	-0.01	0.00	0.00
-2.0	N147	0.00	0.00	0.00	0.00	0.00	0.00	-0.01	0.00	0.00	0.00	0.00	0.00	0.00	0.00	0.00	0.00	0.00	0.00	0.00	0.00	0.00	0.00	0.00	0.00	0.00	-0.42	-0.04	0.01	-1.05	-1.51	-0.81	-0.07	-0.10	-0.02
-1.1	I148	0.00	0.00	0.00	0.00	0.00	0.00	-0.01	0.00	0.00	0.00	0.00	0.00	0.00	0.00	0.00	0.00	0.00	0.00	0.00	0.00	0.00	0.00	0.00	0.00	0.00	-0.01	0.00	0.00	-0.14	-0.84	-0.80	-0.04	-0.10	-0.19
-0.47	V149	0.00	0.00	0.00	0.00	0.00	0.00	0.00	0.00	0.00	0.00	0.00	0.00	0.00	0.00	0.00	0.00	0.00	0.00	0.00	0.00	0.00	0.00	-0.02	0.00	0.00	-0.01	0.00	0.00	-0.12	-0.03	-0.14	-0.12	-0.37	-0.13
-1.1	N150	0.00	0.00	0.00	0.00	0.00	0.00	0.00	0.00	0.00	0.00	0.00	0.00	0.00	0.00	0.00	0.00	0.00	0.00	0.00	0.00	0.00	0.00	-0.02	0.00	0.00	0.00	0.00	0.00	-0.04	-0.01	-0.32	-0.15	-1.2	-0.43
0.10	D151	-0.05	0.00	0.00	0.07	0.08	0.00	-0.10	-0.07	0.00	0.00	0.00	0.00	0.00	0.00	0.06	0.00	0.00	0.00	0.08	0.08	0.08	0.00	0.00	0.00	-0.08	0.10	0.09	0.00	-0.23	-0.01	-0.05	0.11	0.06	-0.01
-0.11	F152	0.00	0.00	0.00	0.00	0.00	0.00	0.00	0.00	0.00	0.00	0.00	0.00	0.00	0.00	0.00	0.00	0.00	0.00	0.00	0.00	0.00	0.00	-0.01	-0.01	0.00	0.00	0.00	0.00	-0.01	-0.02	-0.05	-0.01	-0.02	-0.10
-0.04	T153	0.00	0.00	0.00	0.00	0.00	0.00	0.00	0.00	0.00	0.00	0.00	0.00	0.00	0.00	0.00	0.00	0.00	0.00	0.00	0.00	0.00	0.00	-0.01	0.00	0.00	0.00	0.00	0.00	-0.01	0.00	0.00	0.00	-0.03	-0.02
-0.01	P154	0.00	0.00	0.00	0.00	0.00	0.00	0.00	0.00	0.00	0.00	0.00	0.00	0.00	0.00	0.00	0.00	0.00	0.00	0.00	0.00	0.00	0.00	-0.01	0.00	0.00	0.00	0.00	0.00	0.00	0.00	0.00	0.00	0.00	0.00
0.12	E155	-0.05	0.00	0.00	0.05	0.06	0.00	-0.08	-0.06	0.00	0.00	0.00	0.00	0.00	0.00	0.05	0.00	0.00	0.00	0.07	0.07	0.07	0.00	-0.07	-0.02	-0.07	0.09	0.08	0.00	-0.22	0.00	0.00	0.09	0.09	0.09
0.01	E156	-0.04	0.00	0.00	0.05	0.06	0.00	-0.07	-0.06	0.00	0.00	0.00	0.00	0.00	0.00	0.05	0.00	0.00	0.00	0.07	0.07	0.06	0.00	0.00	0.00	-0.07	0.08	0.07	0.00	-0.50	0.00	0.00	0.08	0.09	0.08
0.22	E157	-0.05	0.00	0.00	0.05	0.06	0.00	-0.07	-0.06	0.00	0.00	0.00	0.00	0.00	0.00	0.05	0.00	0.00	0.00	0.07	0.07	0.06	0.00	0.00	0.00	-0.07	0.08	0.07	0.00	-0.10	0.00	0.00	0.09	0.09	0.10
-0.03	A158	0.00	0.00	0.00	0.00	0.00	0.00	0.00	0.00	0.00	0.00	0.00	0.00	0.00	0.00	0.00	0.00	0.00	0.00	0.00	0.00	0.00	0.00	-0.03	-0.02	0.00	0.00	0.00	0.00	0.00	0.00	0.00	0.00	-0.01	0.00
-0.13	Q159	0.00	0.00	0.00	0.00	0.00	0.00	0.00	0.00	0.00	0.00	0.00	0.00	0.00	0.00	0.00	0.00	0.00	0.00	0.00	0.00	0.00	0.00	-0.02	-0.05	0.00	-0.08	0.00	0.00	-0.09	0.00	0.00	0.00	0.00	0.00
-0.07	V160	0.00	0.00	0.00	0.00	0.00	0.00	0.00	0.00	0.00	0.00	0.00	0.00	0.00	0.00	0.00	0.00	0.00	0.00	0.00	0.00	0.00	0.00	0.00	0.00	0.00	0.00	0.00	0.00	0.00	0.00	0.00	0.00	-0.09	-0.04
-1.1	R161	0.05	0.00	0.00	-0.06	-0.06	0.00	0.09	0.06	0.00	0.00	0.00	0.00	0.00	0.00	-0.05	0.00	0.00	0.00	-0.08	-0.08	-0.07	0.00	-0.01	0.00	0.07	-0.10	-0.08	0.00	0.04	0.03	0.04	-0.62	-0.93	-0.45
0.19	E162	-0.04	0.00	0.00	0.05	0.05	0.00	-0.07	-0.05	0.00	0.00	0.00	0.00	0.00	0.00	0.05	0.00	0.00	0.00	0.07	0.08	0.07	0.00	-0.01	-0.02	-0.07	0.08	0.07	0.00	-0.13	0.00	0.00	0.08	0.09	0.09
0.18	E163	-0.04	0.00	0.00	0.05	0.05	0.00	-0.06	-0.05	0.00	0.00	0.00	0.00	0.00	0.00	0.05	0.00	0.00	0.00	0.07	0.07	0.07	0.00	0.00	-0.01	-0.06	0.09	0.07	0.00	-0.14	0.00	0.00	0.08	0.07	0.07
-0.31	N164	0.00	0.00	0.00	0.00	0.00	0.00	0.00	0.00	0.00	0.00	0.00	0.00	0.00	0.00	0.00	0.00	0.00	0.00	-0.01	0.00	0.00	0.00	-0.12	0.00	0.00	0.00	0.00	0.00	0.00	0.00	0.00	0.00	-0.20	-0.28
-0.60	K165	0.05	0.00	0.00	-0.05	-0.05	0.00	0.07	0.05	0.00	0.00	0.00	0.00	0.00	0.00	-0.05	0.00	0.00	0.00	-0.26	-0.31	-0.08	-0.01	-0.23	-0.01	0.06	-0.08	-0.07	0.00	0.05	0.00	0.00	-0.09	-0.09	-0.09
-1.2	W166	0.00	0.00	0.00	0.00	0.00	0.00	-0.02	0.00	-0.04	0.00	0.00	0.00	0.00	0.00	0.00	0.00	0.00	0.00	-0.04	0.00	-0.01	-0.12	-0.47	-0.01	-0.01	-0.01	-0.01	-0.11	-0.38	-0.01	-0.02	-0.39	-0.20	-0.54
-0.10	C167	0.00	0.00	0.00	0.00	0.00	0.00	0.00	0.00	0.00	0.00	0.00	0.00	0.00	0.00	0.00	0.00	0.00	0.00	-0.01	0.00	0.00	0.00	-0.06	0.00	0.00	0.00	0.00	0.00	-0.04	0.00	0.00	0.00	-0.02	-0.05
-0.01	E168	-0.05	0.00	0.00	0.05	0.05	0.00	-0.08	-0.06	0.00	0.00	0.00	0.00	0.00	0.00	0.05	0.00	0.00	0.00	0.07	0.10	0.08	0.00	-0.14	-0.02	-0.07	0.10	0.07	0.00	-0.42	0.00	0.00	0.12	0.07	0.06
0.15	D169	-0.05	0.00	0.00	0.05	0.05	0.00	-0.08	-0.06	0.00	0.00	0.00	0.00	0.00	0.00	0.05	0.00	0.00	0.00	0.10	0.10	0.08	0.00	-0.03	0.00	-0.07	0.09	0.07	0.00	-0.48	0.00	0.00	0.30	0.07	0.11
0.20	A170	-0.05	0.00	0.00	0.05	0.05	0.00	-0.08	-0.06	0.00	0.00	0.00	0.00	0.00	0.00	0.05	0.00	0.00	0.00	0.08	0.12	0.07	0.00	0.00	0.00	-0.07	0.10	0.07	0.00	-0.14	0.00	-0.01	0.21	-0.14	0.13

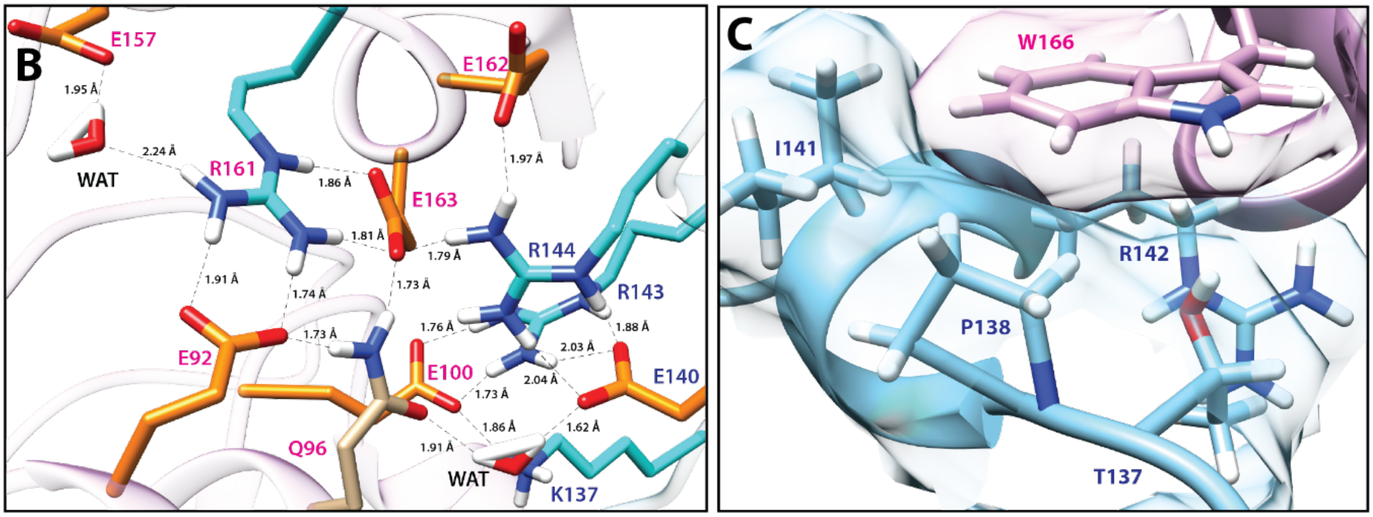

Figure S16. Fuzzy CTR interactions for Simulation 3. (A) Interaction energy matrix showing the pairwise interaction energies for each Skp1 CTR residue. (B) Examples of direct and indirect interactions of oppositely charged residues shown through atom distances. (C) Directly interacting residues are shown as sticks with their Van der Waals surfaces. See Figure 7 for explanation.

Energy (kcal/mol)

>3.0	3.0	2.5	2.0	1.5	1.0	0.50	0.00	-0.50	-1.0	-1.5	-2.0	-2.5	-3.0	<-3.0

A

		K137	T138	P139	E140	E141	I142	R143	R144	I145	F146	N147	I148	V149	N150	D151	F152	T153	P154	E155	E156	E157	A158	Q159	V160	R161	E162	E163	N164	K165	W166	C167	E168	D169	A170
		-0.22	-0.01	-0.02	-0.03	0.10	-0.68	-4.7	-6.0	-2.4	-4.0	-1.9	-1.3	-0.63	-1.8	-3.1	-4.0	-3.8	-1.1	-1.5	0.02	-1.3	-0.50	-0.15	-0.11	-0.25	0.10	0.14	-0.01	-0.21	-0.04	-0.01	0.16	0.16	0.16
-1.3	K137	0.12	-0.01	-0.01	-0.72	-0.59	-0.34	-0.22	0.04	-0.18	-0.01	-0.19	-0.01	-0.03	-0.05	-0.13	-0.01	0.00	0.00	-0.08	-0.07	-0.08	0.00	0.00	0.00	0.07	-0.07	-0.06	0.00	0.07	0.00	0.00	-0.06	-0.05	-0.05
-0.65	T138	0.00	0.00	0.00	-0.02	-0.08	-0.48	-0.15	-0.49	-0.03	-0.01	-0.01	0.00	-0.01	-0.01	0.00	-0.01	0.00	0.00	0.00	0.00	0.00	0.00	0.00	0.00	0.00	0.00	0.00	0.00	0.00	0.00	0.00	0.00	0.00	0.00
-0.45	P139	0.00	0.00	0.00	-0.01	-0.01	-0.02	-0.01	-0.39	-0.04	-0.02	-0.04	0.00	-0.13	-0.12	-0.01	-0.10	0.00	0.00	0.00	0.00	0.00	0.00	0.00	0.00	0.00	0.00	0.00	0.00	0.00	0.00	0.00	0.00	0.00	0.00
-0.52	E140	-0.07	0.00	0.00	0.10	0.11	-0.05	-0.13	-1.31	-0.02	-0.06	-0.02	0.00	-0.05	-0.12	0.14	-0.05	0.00	0.00	0.11	0.09	0.11	0.00	0.00	0.00	-0.10	0.10	0.09	0.00	-0.10	0.00	0.00	0.07	0.07	0.07
-3.1	E141	-0.09	0.00	0.00	0.13	0.14	-0.03	-0.21	-4.5	-0.42	-0.21	-0.69	-0.01	-0.15	-0.81	0.14	-0.01	0.00	0.00	0.10	0.09	0.10	0.00	0.00	0.00	-0.08	0.08	0.07	0.00	-0.09	0.00	0.00	0.06	0.06	0.06
-1.3	I142	0.00	0.00	0.00	-0.01	-0.04	-0.19	-0.05	-0.70	-0.07	-0.06	-0.17	-0.01	-0.45	-0.60	-0.09	-0.06	0.00	-0.01	0.00	0.00	0.00	0.00	0.00	0.00	0.00	0.00	0.00	0.00	0.00	0.00	0.00	0.00	0.00	0.00
-1.5	R143	0.07	0.00	0.00	-0.09	-0.11	-0.01	0.10	-0.65	-0.01	-0.05	-0.02	-0.01	-0.05	-0.96	-0.42	-0.09	-0.05	-0.03	-0.13	-0.12	-0.16	0.00	0.00	0.00	0.11	-0.14	-0.10	0.00	0.14	0.00	0.00	-0.08	-0.08	-0.07
-8.4	R144	0.06	0.00	0.00	-0.08	-0.09	0.00	0.09	0.03	0.00	-0.07	-0.03	-0.01	-0.02	-0.16	-5.0	-1.9	-2.4	-1.2	-2.9	-0.34	-2.3	-0.04	-0.01	-0.01	0.13	-0.14	-0.11	0.00	0.11	0.00	0.00	-0.08	-0.08	-0.07
-4.9	I145	0.00	0.00	0.00	0.00	0.00	0.00	0.00	-0.44	-0.09	-1.5	-0.26	-0.04	-0.02	-0.63	-1.4	-2.9	-2.3	-0.16	0.00	0.00	-0.01	-0.01	-0.01	0.00	0.00	0.00	0.00	0.00	0.01	0.00	0.00	0.00	0.00	0.00
-3.0	F146	0.00	0.00	0.00	0.00	0.00	0.00	0.00	-0.04	-0.01	-1.1	-0.03	-0.13	-0.01	-0.05	0.01	-0.61	-1.9	-0.62	-0.03	-0.05	-0.63	-0.42	-0.05	-0.17	-0.07	-0.07	0.00	-0.01	-0.01	0.00	0.00	0.00	0.00	0.00
-0.81	N147	0.00	0.00	0.00	0.00	0.00	0.00	0.00	-0.01	-0.03	-0.42	-0.01	-0.01	0.00	-0.01	-0.01	-0.54	-0.37	-0.03	-0.02	-0.01	-0.01	-0.08	-0.02	-0.01	-0.01	-0.01	0.00	0.00	-0.01	0.00	0.00	0.00	0.00	0.00
-2.1	I148	0.00	0.00	0.00	0.00	0.00	0.00	-0.01	-0.02	-0.38	-0.94	-0.10	-0.17	-0.01	-0.01	-0.01	-0.77	-0.41	-0.12	-0.62	-0.03	-0.02	-0.42	-0.19	-0.01	0.00	0.00	0.00	0.00	0.00	0.00	0.00	0.00	0.00	0.00
-0.44	V149	0.00	0.00	0.00	0.00	0.00	0.00	0.00	-0.01	-0.03	-0.18	-0.01	-0.01	0.00	0.00	-0.01	-0.46	-0.04	-0.01	-0.07	0.00	-0.01	-0.01	-0.01	0.00	0.00	0.00	0.00	0.00	0.00	0.00	0.00	0.00	0.00	-0.01
-0.51	N150	0.00	0.00	0.00	0.00	0.00	0.00	0.00	-0.06	-0.21	-0.39	-0.01	-0.01	0.00	0.00	0.00	-0.28	-0.01	-0.01	-0.02	0.00	0.00	0.00	0.00	0.00	0.00	0.00	0.00	0.00	0.00	0.00	0.00	0.00	0.00	0.00
-0.79	D151	-0.08	0.00	0.00	0.10	0.16	-0.01	-0.70	-0.50	-0.73	-0.33	-0.01	-0.01	0.00	0.00	0.10	-0.02	0.00	0.00	0.11	0.09	0.09	0.00	0.00	0.00	-0.09	0.08	0.07	0.00	-0.09	0.00	0.00	0.07	0.07	0.07
-2.8	F152	0.00	0.00	0.00	0.00	-0.02	-0.14	-1.5	-2.3	-0.53	-0.88	-0.02	0.00	0.00	-0.02	-0.01	-0.14	-0.01	0.00	0.00	0.00	0.00	0.00	0.00	0.00	0.00	0.00	0.00	0.00	0.00	0.00	0.00	0.00	0.00	0.00
-0.60	T153	0.00	0.00	0.00	0.00	0.00	-0.02	-0.88	-0.06	-0.18	-0.04	0.00	0.00	0.00	0.00	0.00	0.00	0.00	0.00	0.00	0.00	0.00	0.00	0.00	0.00	0.00	0.00	0.00	0.00	0.00	0.00	0.00	0.00	0.00	0.00
-0.22	P154	0.00	0.00	0.00	0.00	0.01	-0.02	-0.40	-0.02	-0.01	0.00	0.00	0.00	0.00	0.00	0.00	0.00	0.00	0.00	0.00	0.00	0.00	0.00	0.00	0.00	0.00	0.00	0.00	0.00	0.00	0.00	0.00	0.00	0.00	0.00
0.15	E155	-0.07	0.00	0.00	0.08	0.12	0.00	-0.18	-0.12	-0.01	0.00	0.00	0.00	0.00	0.00	0.08	0.00	0.00	0.00	0.09	0.07	0.08	0.00	0.00	0.00	-0.08	0.07	0.06	0.00	-0.08	0.00	0.00	0.07	0.07	0.07
0.13	E156	-0.08	0.00	0.00	0.09	0.13	0.00	-0.17	-0.11	0.00	0.00	0.00	0.00	0.00	0.00	0.07	0.00	0.00	0.00	0.07	0.06	0.06	0.00	0.00	0.00	-0.06	0.06	0.05	0.00	-0.07	0.00	0.00	0.06	0.06	0.06
-2.1	E157	-0.10	-0.01	0.00	0.13	0.28	-0.02	-4.7	-0.13	-0.02	0.00	0.00	0.00	0.00	0.00	0.08	0.00	0.00	0.00	0.08	0.06	0.07	0.00	0.00	0.00	-0.06	0.06	0.05	0.00	-0.07	0.00	0.00	0.05	0.05	0.05
-0.04	A158	0.00	0.00	0.00	0.00	0.00	0.00	-0.03	0.00	-0.04	0.00	0.00	0.00	0.00	0.00	0.00	0.00	0.00	0.00	0.00	0.00	0.00	0.00	0.00	0.00	0.00	0.00	0.00	0.00	0.00	0.00	0.00	0.00	0.00	0.00
-0.01	Q159	0.00	0.00	0.00	0.00	0.00	0.00	0.00	0.00	-0.01	0.00	0.00	0.00	0.00	0.00	0.00	0.00	0.00	0.00	0.00	0.00	0.00	0.00	0.00	0.00	0.00	0.00	0.00	0.00	0.00	0.00	0.00	0.00	0.00	0.00
-0.34	V160	0.00	0.00	0.00	0.00	-0.03	0.00	-0.02	0.00	-0.53	-0.05	-0.02	-0.01	0.00	0.00	0.00	0.00	0.00	0.00	0.00	0.00	0.00	0.00	0.00	0.00	0.00	0.00	0.00	0.00	0.00	0.00	0.00	0.00	0.00	0.00
-0.67	R161	0.08	0.00	-0.02	-0.10	-0.15	0.00	0.16	0.11	-0.48	-0.37	-0.02	-0.02	0.00	0.00	-0.09	-0.01	0.00	0.00	-0.09	-0.08	-0.08	0.00	0.00	0.00	0.07	-0.07	-0.06	0.00	0.08	0.00	0.00	-0.06	-0.06	-0.06
0.15	E162	-0.06	0.00	0.00	0.08	0.10	0.00	-0.12	-0.10	-0.03	0.00	-0.01	-0.01	0.00	0.00	0.08	0.00	0.00	0.00	0.08	0.07	0.07	0.00	0.00	0.00	-0.07	0.06	0.06	0.00	-0.07	0.00	0.00	0.06	0.06	0.06
0.11	E163	-0.07	0.00	0.00	0.09	0.11	0.00	-0.12	-0.10	-0.02	-0.01	-0.04	-0.02	-0.01	0.00	0.08	0.00	0.00	0.00	0.07	0.06	0.07	0.00	0.00	0.00	-0.06	0.06	0.05	0.00	-0.06	0.00	0.00	0.05	0.05	0.05
-1.3	N164	0.00	0.00	0.00	0.00	-0.01	0.00	0.00	0.00	-0.56	-0.53	-1.2	-0.26	-0.02	-0.02	0.00	-0.01	0.00	0.00	0.00	0.00	0.00	0.00	0.00	0.00	0.00	0.00	0.00	0.00	0.00	-0.01	0.00	0.00	0.00	0.00
-0.35	K165	0.07	0.00	0.00	-0.08	-0.10	0.00	0.10	0.09	-0.02	-0.03	-0.10	-0.16	-0.02	0.00	-0.08	0.00	0.00	0.00	-0.07	-0.07	-0.07	0.00	0.00	0.00	0.07	-0.06	-0.06	0.00	0.07	0.00	0.00	-0.06	-0.06	-0.06
-1.6	W166	0.00	0.00	0.00	0.00	0.00	0.00	-0.01	-0.01	-0.09	-0.73	-0.60	-1.4	-0.10	-0.02	0.00	-0.02	-0.01	0.00	0.00	0.00	-0.01	0.00	0.00	-0.02	-0.01	0.00	0.00	-0.01	-0.02	-0.04	-0.01	0.00	0.00	0.00
-0.16	C167	0.00	0.00	0.00	0.00	0.00	0.00	0.00	0.00	0.00	-0.04	-0.05	-0.14	-0.06	0.00	0.00	-0.01	0.00	0.00	0.00	0.00	0.00	0.00	0.00	0.00	0.00	0.00	0.00	0.00	0.00	0.00	0.00	0.00	0.00	0.00
0.12	E168	-0.07	0.00	0.00	0.08	0.09	0.00	-0.10	-0.11	0.00	0.00	-0.02	-0.03	-0.02	0.00	0.08	0.00	0.00	0.00	0.08	0.07	0.08	0.00	0.00	0.00	-0.08	0.07	0.06	0.00	-0.07	0.00	0.00	0.06	0.06	0.06
0.12	D169	-0.07	0.00	0.00	0.08	0.09	0.00	-0.09	-0.08	0.00	0.00	-0.01	-0.08	-0.04	0.00	0.08	-0.01	0.00	0.00	0.08	0.07	0.08	0.00	0.00	0.00	-0.08	0.07	0.06	0.00	-0.07	0.00	0.00	0.06	0.06	0.06
0.09	A170	-0.06	0.00	0.00	0.07	0.08	0.00	-0.09	-0.08	0.00	0.00	0.00	-0.08	-0.04	0.00	0.08	-0.03	-0.01	-0.01	0.08	0.07	0.08	0.00	0.00	0.00	-0.09	0.07	0.06	0.00	-0.08	0.00	0.00	0.06	0.06	0.06

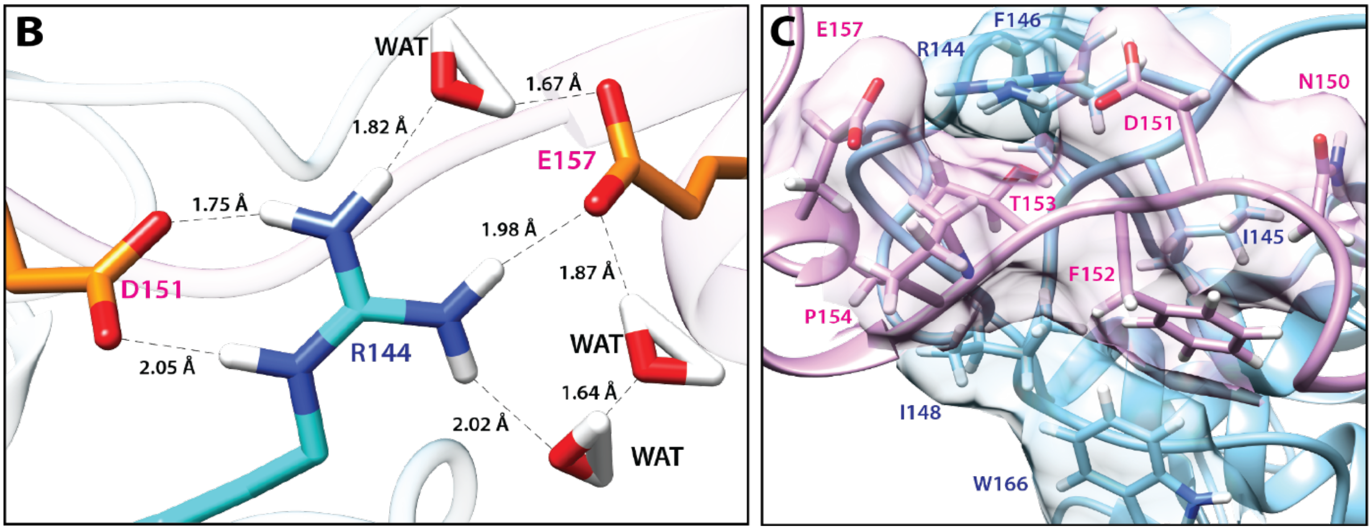

Figure S17. Distances between interacting charge clusters through the MD simulations. Distances are shown for the interactions of oppositely charged residues indicated to have an interaction energy of <–1 kcal/mol across all three simulations. Distances within 8 Å are highlighted in a yellow box. Residues are color coded as above.

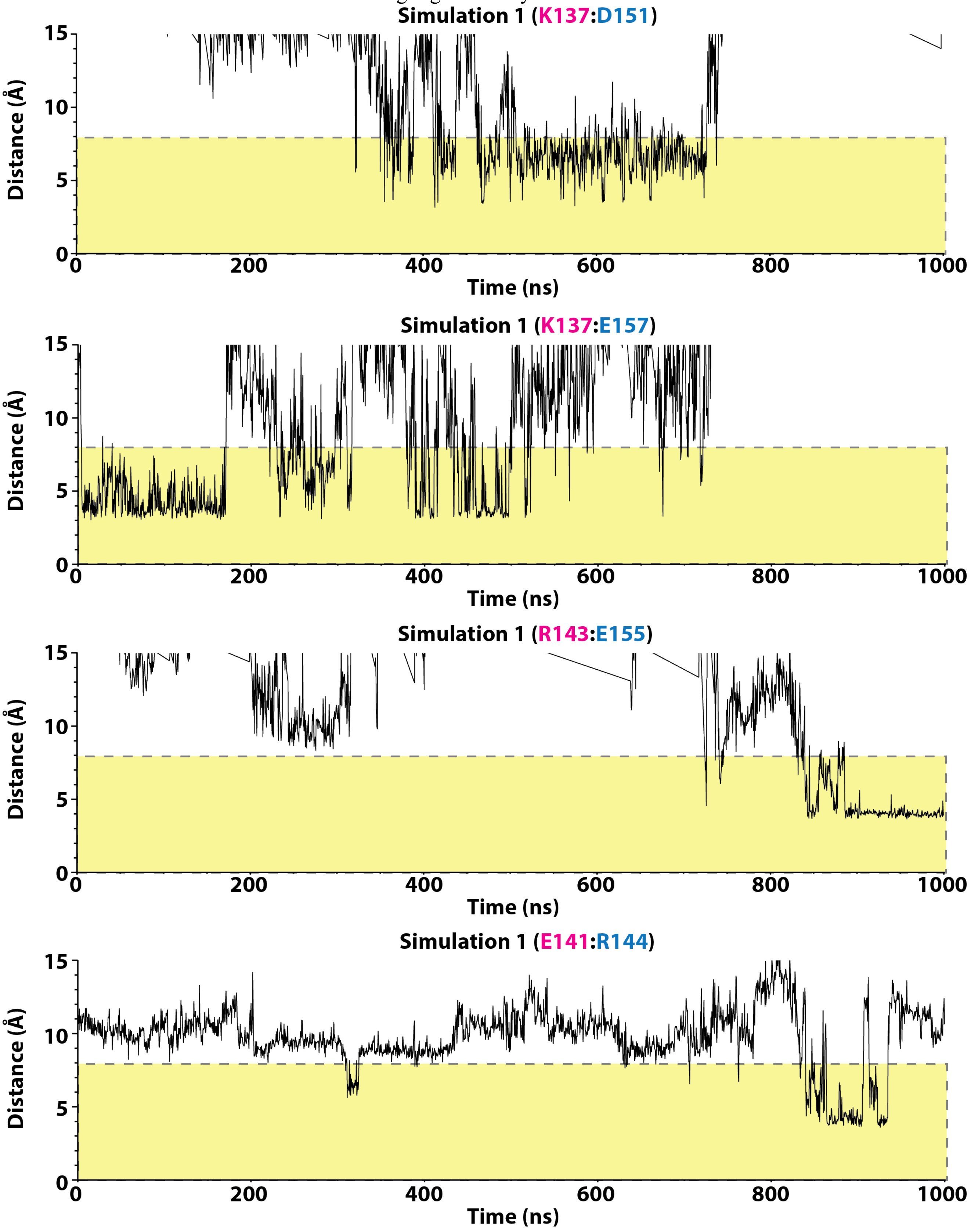

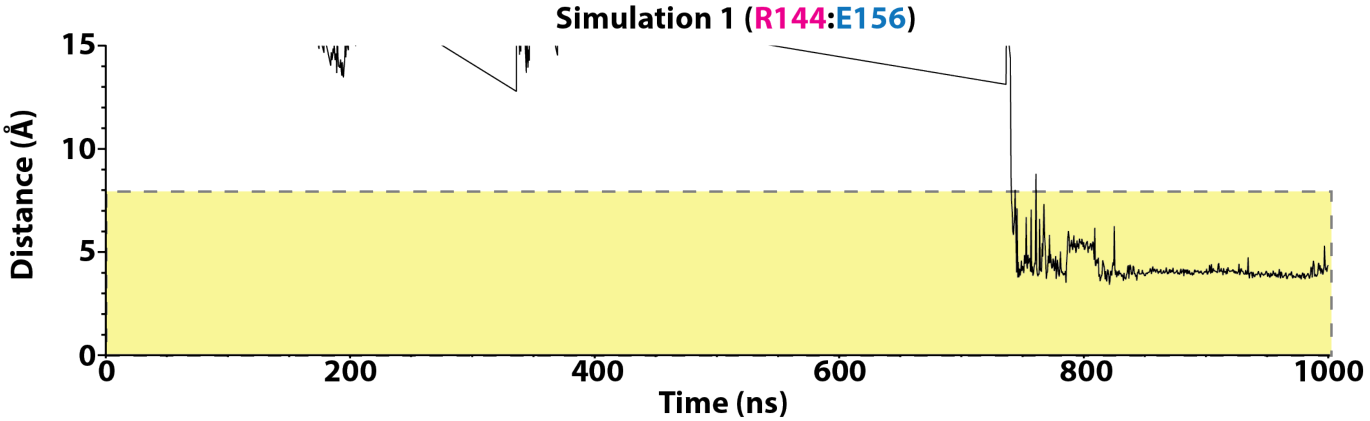
Simulation 1 (R144:E156)
Distance (Å)
Time (ns)
0
5
10
15
0
200
400
600
800
1000

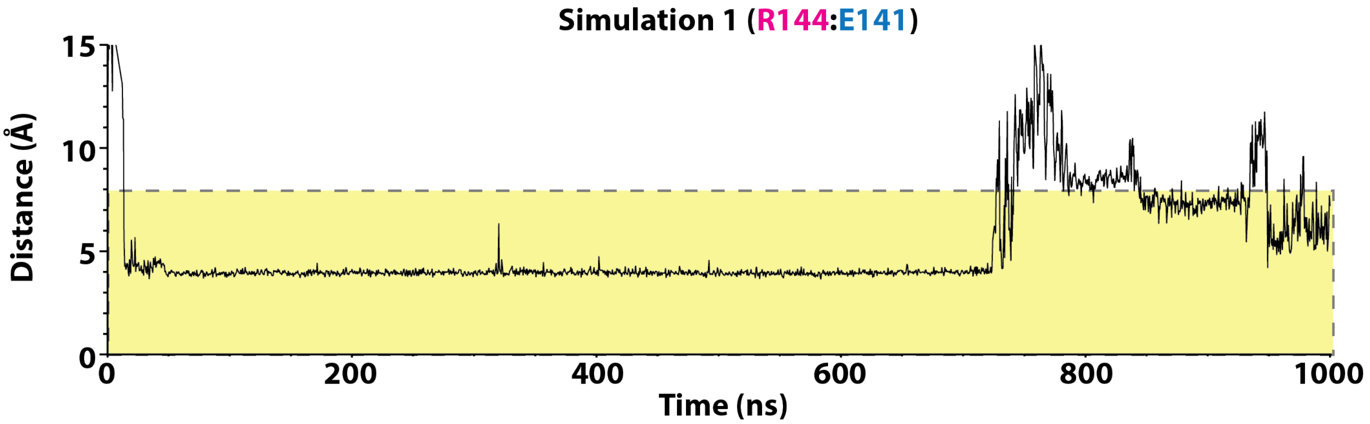
Simulation 1 (R144:E141)
Distance (Å)
Time (ns)
0
5
10
15
0
200
400
600
800
1000

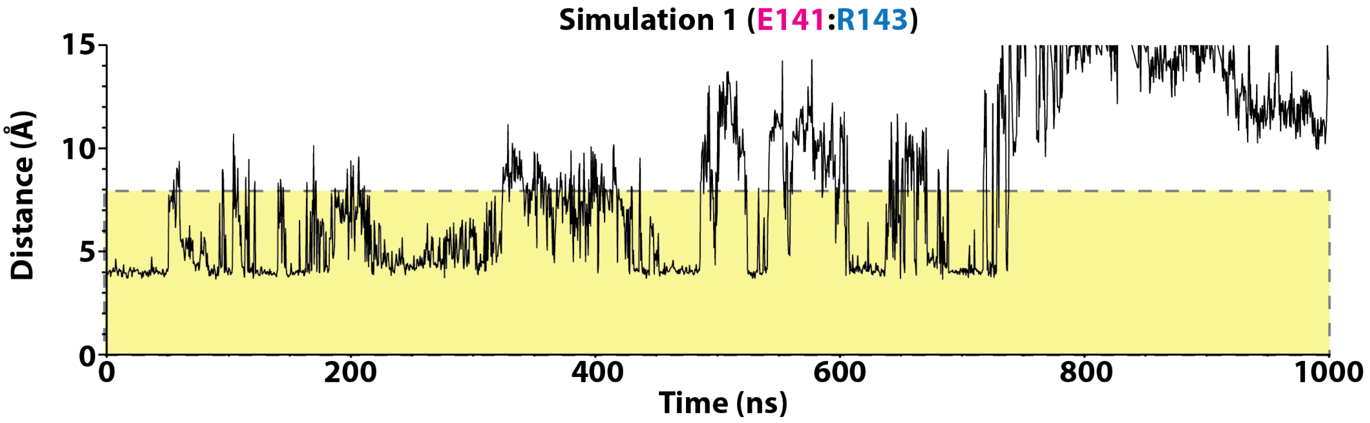
Simulation 1 (E141:R143)
Distance (Å)
Time (ns)
0
5
10
15
0
200
400
600
800
1000

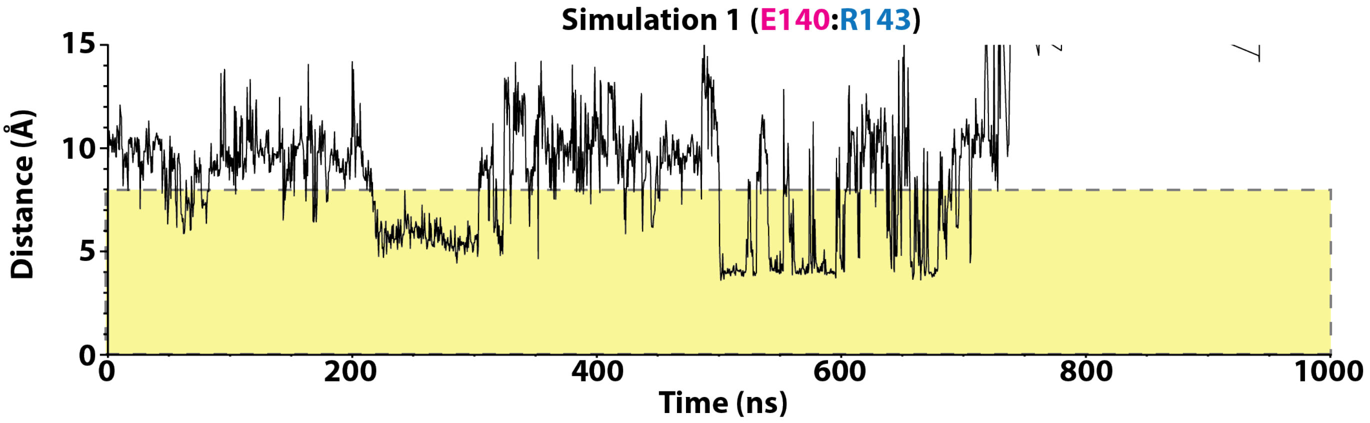
Simulation 1 (E140:R143)
Distance (Å)
Time (ns)
0
5
10
15
0
200
400
600
800
1000

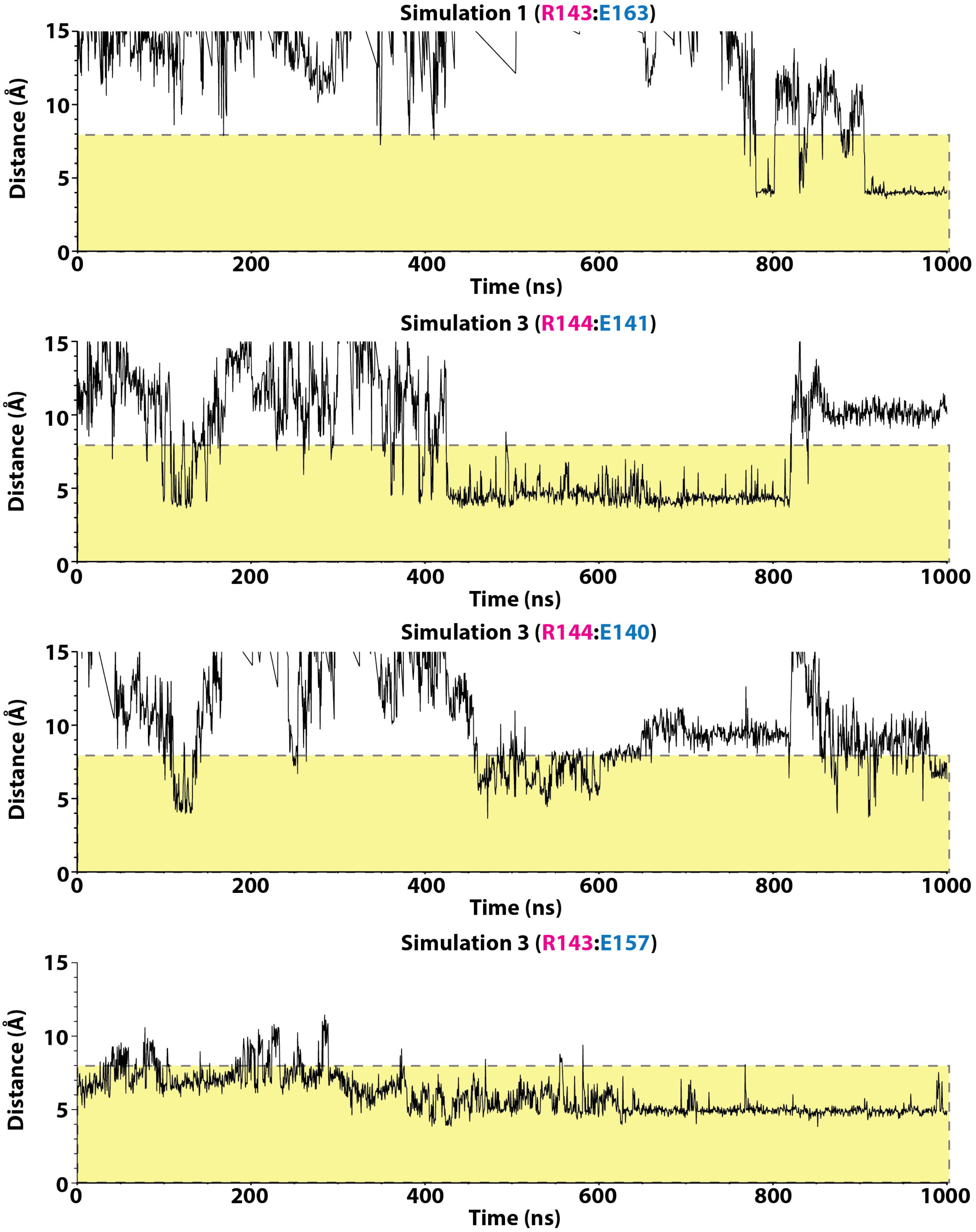
Simulation 1 (R143:E163)
Simulation 3 (R144:E141)
Simulation 3 (R144:E140)
Simulation 3 (R143:E157)
Distance (Å)
Time (ns)
0
5
10
15
200
400
600
800
1000

Simulation 3 (E157:R144)

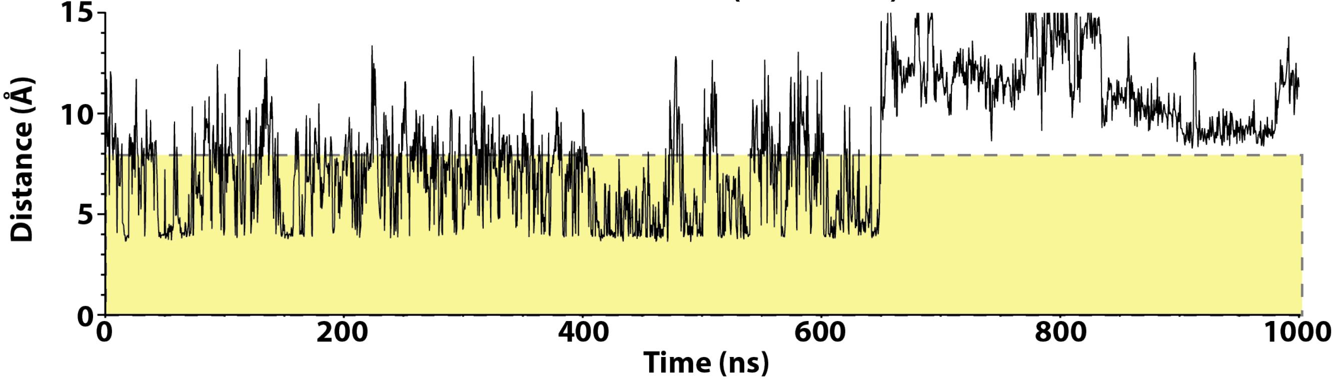

Simulation 3 (E155:R144)

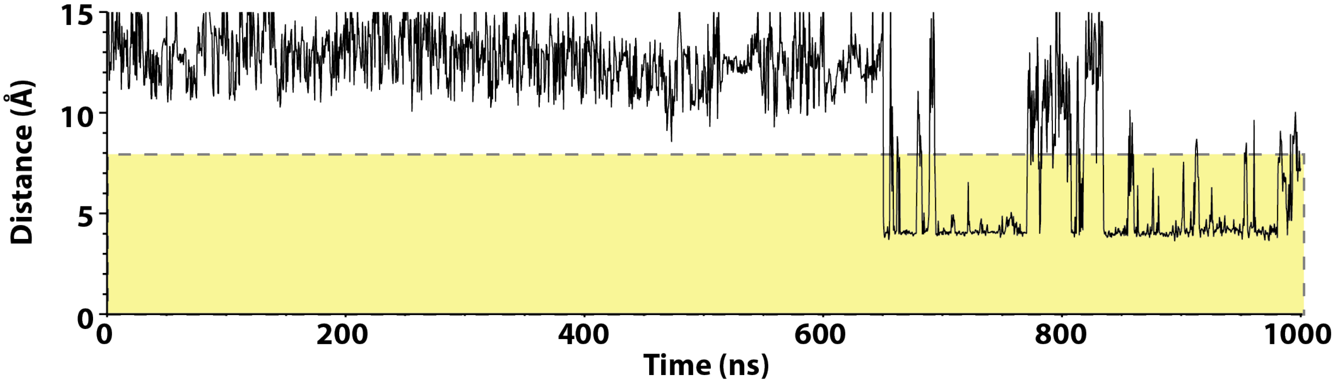

Simulation 3 (D151:R144)

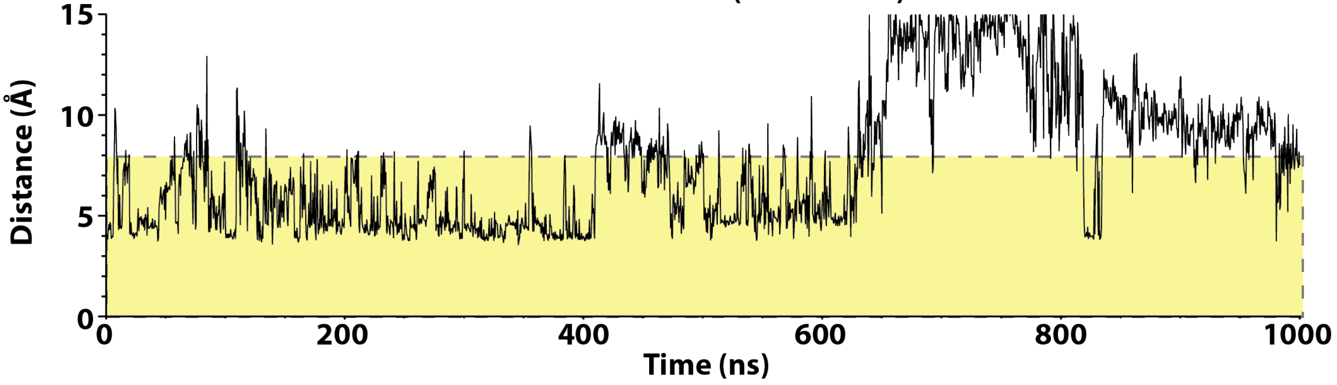

Simulation 2 (E163:R144)

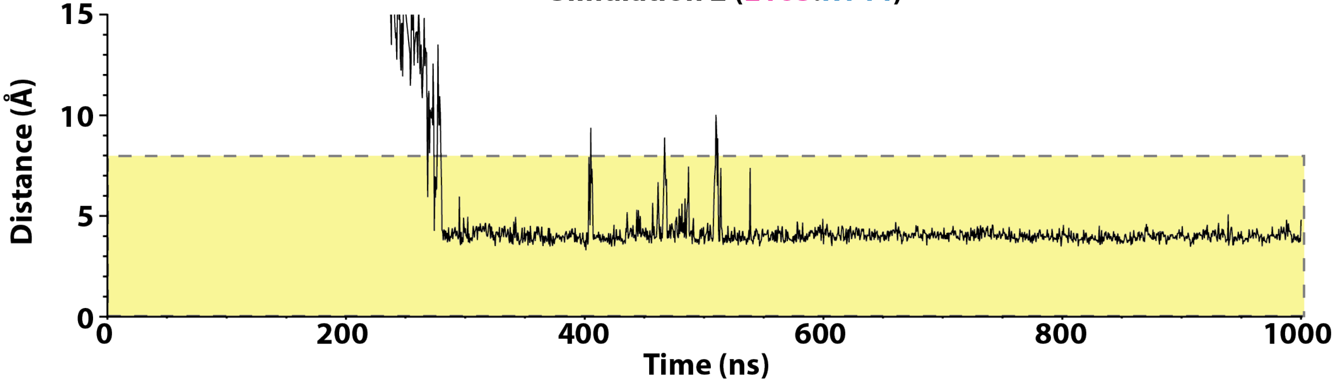

Simulation 2 (E163:R143)

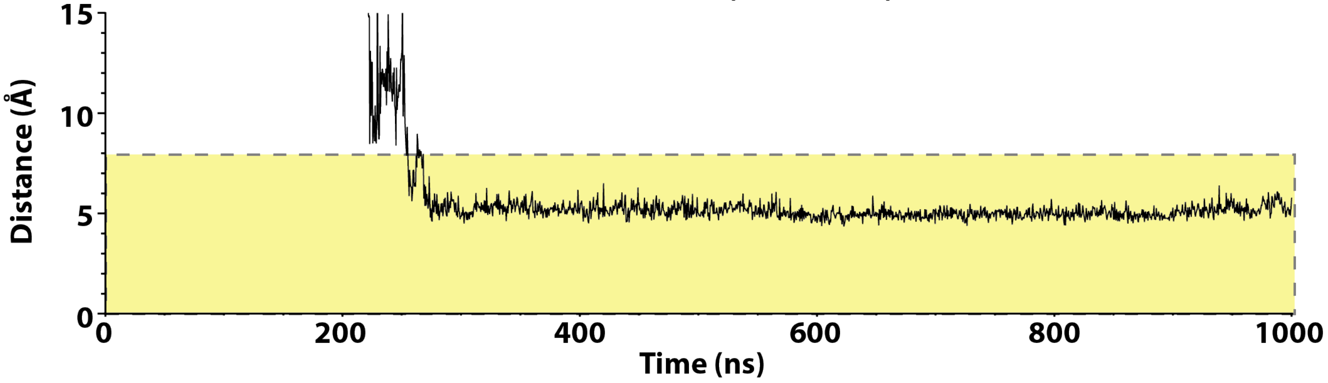

Simulation 2 (E162:R144)

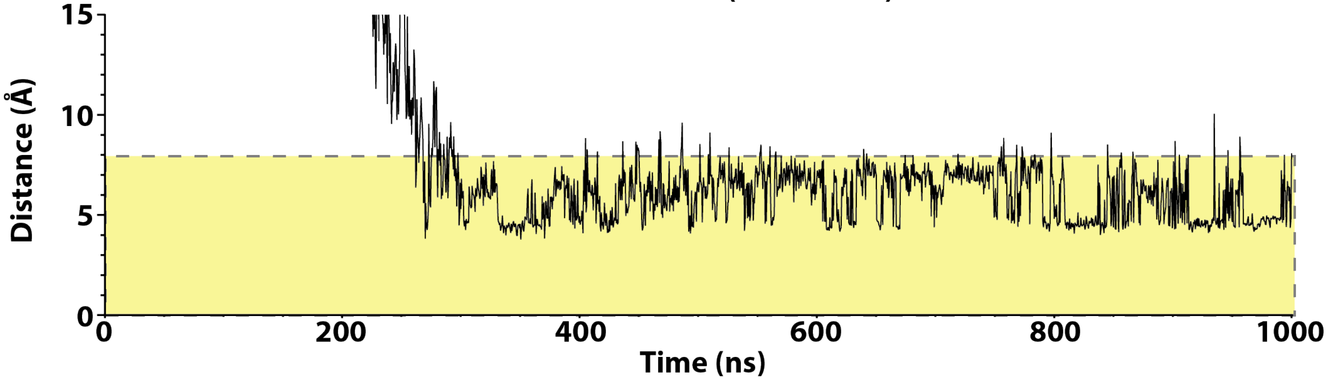

Simulation 2 (E162:R143)

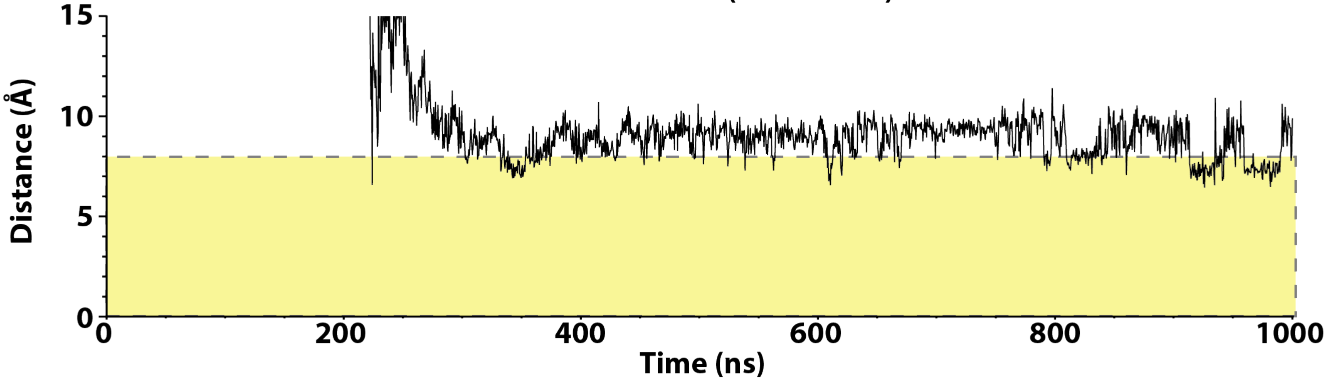

Figure S18. Overlaps between the dimer-mate of the Skp1 monomer and adjacent SCF components**.** Backbone atom overlays of SCF components (from a Cul1/Rbx1/Skp1/F-box(Skp2); PDB: 1LDK) and the Skp1 homodimer (PDB: 6V88). The representative SCF complex contains Skp1 (green), FBP(Skp2, magenta), and Cullin1 (blue). The Skp1 homodimer contains two subunits with one (light grey) overlaying well with the SCF bound form of Skp1 and the other (dark grey) sterically clashing with both Skp2 and Cul1. Four poses are represented (A-D).

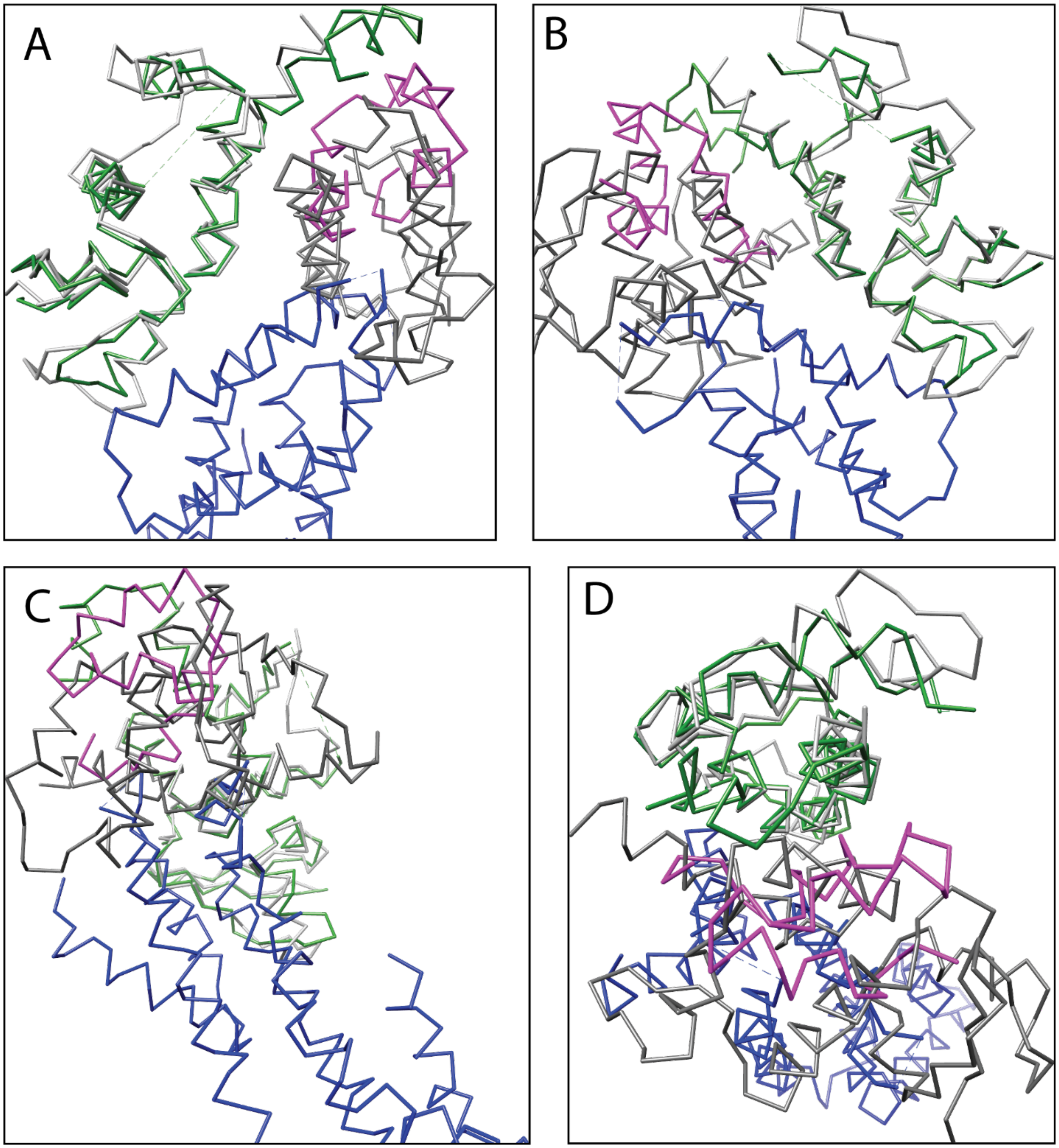

Figure S19. Engineering TgSkp1 deletions. (A) Primer pairs used to delete the C-Terminal Region (CTR), the internal loop, or both (starting with the internal loop) of TgSkp1 (UniProt A0A0F7UNE6). Clones were screened by colony PCR utilizing flanking primers annealing with the pNIC28 plasmid backbone. (B) Sequence of the pNIC28-TgSkp1-FL parental plasmid utilized for deletions. The coding region of the original plasmid is underlined in black with amino acids above in bold black. The TEV-cleavage motif is highlighted in yellow with the cut site in teal. Primers are shown in red with annealing residues capitalized. The resulting deletions are indicated below each site in blue. Residues are numbered relative to the native start site (Met).

A.

Primer	Sequence
TgSkp1_ΔCTR-F	5'-gatcaaagccTGACAGTAAAGGTGGATACGGATCCG
TgSkp1_ΔCTR-R	5'-tttactgtcaGGCTTTGATCATTGTCGCCACTTTC
TgSkp1_ΔLoop-F	5'-aacaacccaggtggttccggaCTTGCTGAAGTGGTCAGCGAGTG
TgSkp1_ΔLoop-R	5'-ttcagcaagtccggaaccaccTGGGTTGTTGTGGTGATGTTCAC
pNIC28_Screening-F	5'-CGGGGCCTGCCACCATACCCACG
pNIC28_Screening-R	5'-GTGGTGGTGGTGGTGCTCGAGTGCGG

B.

>pNIC28-HisTEVTgSkp1-FL

```
GCCACGGGGCCTGCCACCATACCCACGCCGAAACAAGCGCTCATGAGCCCGAAGTGGCGA
 5'-CGGGGCCTGCCACCATACCCACG                                      pNIC28_Screening-F

GCCCGATCTTCCCCATCGGTGATGTCGGCGATATAGGCGCCAGCAACCGCACCTGTGGCG
CCGGTGATGCCGGCCACGATGCGTCCGGCGTAGAGGATCGAGATCTCGATCCCGCGAAAT

                             M  H  H  H  H  H  H  S  S  G  V
TGTTTAACTTTAAGAAGGAGATATACATATGCACCATCATCATCATCATTCTTCTGGTGT

 D  L  G  T  E  N  L  Y  F  Q><S  M  S  K  E  R  M  G  D  A   A9
AGATCTGGGTACCGAGAACCTGTACTTCCAATCCATGTCAAAAGAAAGAATGGGAGATGC  TEV Site

 R  K  V  T  L  V  S  Q  E  G  D  E  F  D  V  D  I  E  V  A   A29
CCGGAAGGTCACTCTCGTCTCGCAAGAGGGAGACGAGTTTGATGTTGACATCGAGGTCGC

 S  M  S  A  L  I  K  T  M  V  E  E  D  S  D  C  Q  E  S  I   I49
CTCCATGAGTGCGTTGATAAAGACAATGGTGGAAGAAGACAGCGACTGCCAAGAGAGCAT

 P  L  P  N  V  D  T  C  I  L  K  K  I  I  E  Y  C  E  H  H   H69
TCCTCTTCCCAACGTGGATACATGCATTCTGAAGAAAATCATCGAGTATTGTGAACATCA
                                                  CACTTGTAGT  TgSkp1_ΔLoop-R

 H  N  N  P  P  E  E  I  P  K  P  L  K  S  S  N  L  A  E  V   V89
CCACAACAACCCACCCGAAGAAATCCCCAAGCCTCTAAAGTCGTCCAACCTTGCTGAAGT
GGTGTTGTTGGGTccaccaaggcctgaacgactt-5'
                          5'-aacaacccaggtggttccggaCTTGCTGAAGT  TgSkp1_ΔLoop-F

 H  N  N  P  -  -  -  -  -  -  -  -  G  G  S  G  L  A  E  V   ΔLoop
CCACAACAACCCA------------------------GGTGGTTCCGGACTTGCTGAAGT  or ΔLoopΔCTR

 V  S  E  W  D  Y  Q  F  I  N  E  N  S  D  Q  K  I  L  F  A   A109
GGTCAGCGAGTGGGACTACCAATTCATCAACGAAAACAGCGACCAGAAGATTTTGTTTGC
GGTCAGCGAGTG

 L  I  L  A  A  N  Y  L  N  I  K  P  L  L  D  L  S  V  A  K   K129
TTTGATTTTGGCGGCTAACTACCTCAACATCAAGCCGCTGCTGGATCTGAGCGTCGCGAA
                                                         CTT  TgSkp1_ΔCTR-R
```

```
 V  A  T  M  I  K  A  K  T  P  E  E  I  R  R  I  F  N  I  V   V149
AGTGGCGACAATGATCAAAGCCAAGACGCCGGAAGAAATCCGACGCATTTTCAACATCGT
TCACCGCTGTTACTAGTTTCGGactgtcattt-5’

 V  A  T  M  I  K  A  -  -  -  -  -  -  -  -  -  -  -  -  -   ΔCTR
AGTGGCGACAATGATCAAAGCC--------------------------------------   or ΔLoopΔCTR

 N  D  F  T  P  E  E  E  A  Q  V  R  E  E  N  K  W  C  E  D   D169
CAATGACTTCACTCCTGAGGAAGAAGCTCAGGTGCGCGAGGAGAACAAGTGGTGCGAAGA
                                                  5’-gatcaa   TgSkp1_ΔCTR-F
 -  -  -  -  -  -  -  -  -  -  -  -  -  -  -  -  -  -  -  -   ΔCTR
------------------------------------------------------------   or ΔLoopΔCTR

 A  *                                                          A170
TGCATGACAGTAAAGGTGGATACGGATCCGAATTCGAGCTCCGTCGACAAGCTTGCGGCC
agccTGACAGTAAAGGTGGATACGGATCCG
                                                          GG   pNIC28_Screening-R

 -  *                                                          ΔCTR or ΔLoopΔCTR
----TGACAGTAAAGGTGGATACGGATCCGAATTCGAGCTCCGTCGACAAGCTTGCGGCC

GCACTCGAGCACCACCACCACCACCACTGAGATCCGGCTGCTAACAAAGCCCGAAAGGAA
CGTGAGCTCGTGGTGGTGGTGGTG-5’
```

Figure S20. Sequences of chemically synthesized variants of TgSkp1 genes. The pNIC28-TgSkp1 plasmid served as the plasmid backbone for gene synthesis products and its sequence (underlined) and translation of the open reading frame (bold) are shown in black. The TEV-cleavage motif is highlighted in yellow with the cut site in teal. The alternative C-terminal regions (CTRs) are represented as different colors. Amino acid residues are numbered relative to the native Met start site.

```
>pNIC28-HisTEVTgSkp1-(PolySer; Scrambled5/Scr-5; Scrambled6/Scr-6)
TAATACGACTCACTATAGGGGAATTGTGAGCGGATAACAATTCCCCTCTAGAAATAATTT

                               M  H  H  H  H  H  H  S  S  G  V
TGTTTAACTTTAAGAAGGAGATATACATATGCACCATCATCATCATCATTCTTCTGGTGT

 D  L  G  T  E  N  L  Y  F  Q><S  M  S  K  E  R  M  G  D  A      A9
AGATCTGGGTACCGAGAACCTGTACTTCCAATCCATGTCAAAAGAAAGAATGGGAGATGC

 R  K  V  T  L  V  S  Q  E  G  D  E  F  D  V  D  I  E  V  A      A29
CCGGAAGGTCACTCTCGTCTCGCAAGAGGGAGACGAGTTTGATGTTGACATCGAGGTCGC

 S  M  S  A  L  I  K  T  M  V  E  E  D  S  D  C  Q  E  S  I      I49
CTCCATGAGTGCGTTGATAAAGACAATGGTGGAAGAAGACAGCGACTGCCAAGAGAGCAT

 P  L  P  N  V  D  T  C  I  L  K  K  I  I  E  Y  C  E  H  H      H69
TCCTCTTCCCAACGTGGATACATGCATTCTGAAGAAAATCATCGAGTATTGTGAACATCA

 H  N  N  P  P  E  E  I  P  K  P  L  K  S  S  N  L  A  E  V      V89
CCACAACAACCCACCCGAAGAAATCCCCAAGCCTCTAAAGTCGTCCAACCTTGCTGAAGT

 V  S  E  W  D  Y  Q  F  I  N  E  N  S  D  Q  K  I  L  F  A      A109
GGTCAGCGAGTGGGACTACCAATTCATCAACGAAAACAGCGACCAGAAGATTTTGTTTGC

 L  I  L  A  A  N  Y  L  N  I  K  P  L  L  D  L  S  V  A  K      K129
TTTGATTTTGGCGGCTAACTACCTCAACATCAAGCCGCTGCTGGACTTAAGCGTTGCTAA

 V  A  T  M  I  K  A  S  S  S  S  S  S  S  S  S  S  S  S  S      S149
AGTTGCGACCATGATTAAGGCTAGCTCCAGCTCATCTAGCTCTTCTAGCAGTTCCAGCTC      polySer
 V  A  T  M  I  K  A  E  E  I  E  P  D  Q  K  E  E  I  D  F      F149
AGTTGCCACTATGATTAAGGCGGAAGAAATTGAACCCGACCAAAAGGAAGAAATCGACTT      Scr-5
 V  A  T  M  I  K  A  E  A  E  K  P  V  A  E  I  P  T  E  R      R149
AGTTGCCACTATGATTAAGGCGGAAGCGGAGAAGCCGGTGGCTGAGATCCCGACCGAGCG      Scr-6

 S  S  S  S  S  S  S  S  S  S  S  S  S  S  S  S  S  S  S  S      S169
CTCCAGCTCCTCTTCTTCCAGCTCCAGCAGCAGCTCATCTAGCTCGTCTTCCAGCTCGTC      polySer
 N  R  A  E  C  T  N  R  P  R  V  I  N  T  W  E  E  K  V  A      A169
CAACCGGGCTGAGTGCACTAACAGACCCCGGGTCATTAACACTTGGGAAGAAAAGGTGGC      Scr-5
 T  V  N  F  I  N  Q  R  E  E  K  R  N  E  C  I  D  D  F  W      W169
CACCGTTAATTTCATCAACCAGCGTGAAGAGAAGCGCAACGAGTGCATTGATGATTTCTG      Scr-6

 S  *                                                             S170
GTCCTAAGGATCCGAATTCGAGCTCCGTCGACAAGCTTGCGGCCGCACTCGAGCACCACC      polySer
 F  *                                                             F170
GTTTTAAGGATCCGAATTCGAGCTCCGTCGACAAGCTTGCGGCCGCACTCGAGCACCACC      Scr-5
 E  *                                                             E170
GGAATAAGGATCCGAATTCGAGCTCCGTCGACAAGCTTGCGGCCGCACTCGAGCACCACC      Scr-6

ACCACCACCACTGAGATCCGGCTGCTAACAAAGCCCGAAAGGAAGCTGAGTTGGCTGCTG
CCACCGCTGAGCAATAACTAGCATAACCCCTTGGGGCCTCTAAACGGGTCTTGAGGGGTT
```
